# Comparative hybridization reveals extensive genome variation in the AIDS-associated pathogen *Cryptococcus neoformans*

**DOI:** 10.1186/gb-2008-9-2-r41

**Published:** 2008-02-22

**Authors:** Guanggan Hu, Iris Liu, Anita Sham, Jason E Stajich, Fred S Dietrich, James W Kronstad

**Affiliations:** 1 Michael Smith Laboratories, The University of British Columbia, 2185 East Mall, Vancouver, British Columbia V6T 1Z4, Canada; 2 Department of Molecular Genetics and Microbiology, Institute for Genome Sciences and Policy, 287 CARL Building, Duke University Medical Center, Durham, NC 27710, USA; 3 Department of Plant and Microbial Biology, 121 Koshland Hall, University of California, Berkeley, CA 94720-3102, USA

## Abstract

**Background:**

Genome variability can have a profound influence on the virulence of pathogenic microbes. The availability of genome sequences for two strains of the AIDS-associated fungal pathogen *Cryptococcus neoformans *presented an opportunity to use comparative genome hybridization (CGH) to examine genome variability between strains of different mating type, molecular subtype, and ploidy.

**Results:**

Initially, CGH was used to compare the approximately 100 kilobase *MAT***a **and *MATα *mating-type regions in serotype A and D strains to establish the relationship between the Log2 ratios of hybridization signals and sequence identity. Subsequently, we compared the genomes of the environmental isolate NIH433 (*MAT***a**) and the clinical isolate NIH12 (*MATα*) with a tiling array of the genome of the laboratory strain JEC21 derived from these strains. In this case, CGH identified putative recombination sites and the origins of specific segments of the JEC21 genome. Similarly, CGH analysis revealed marked variability in the genomes of strains representing the VNI, VNII, and VNB molecular subtypes of the A serotype, including disomy for chromosome 13 in two strains. Additionally, CGH identified differences in chromosome content between three strains with the hybrid AD serotype and revealed that chromosome 1 from the serotype A genome is preferentially retained in all three strains.

**Conclusion:**

The genomes of serotypes A, D, and AD strains exhibit extensive variation that spans the range from small differences (such as regions of divergence, deletion, or amplification) to the unexpected disomy for chromosome 13 in haploid strains and preferential retention of specific chromosomes in naturally occurring diploids.

## Abstract 2

Extensive genome variation in the AIDS-associated pathogen *Cryptococcus neoformans* is revealed through comparative genome hybridization between strains of different mating type, molecular subtype and ploidy.

## Background

The encapsulated, basidiomycetous fungi *Cryptococcus neoformans *and *C. gattii *cause life-threatening meningoencephalitis and pose a significant threat to AIDS patients [1-3]. Four serotypes (A to D) of these fungi are recognized, based on antigenic differences in the capsule polysaccharide, which is one of the major virulence factors. *C. neoformans *isolates have A or D capsular serotypes and mainly infect immunocompromised individuals [4]. Isolates of serotypes B and C were recently re-classified as the separate species *C. gattii*, which infects both immunocompromised and immunocompetent patients [3,5-7]. Strains with hybrid serotypes (AD and BD) have also been identified from both clinical and environmental sources [8-11]. Serotype A strains are the most prevalent clinical isolates and account for the majority of infections in AIDS patients. Serotype D isolates account for fewer cases of cryptococcosis, and some of these infections may actually involve AD hybrid strains [12]. Serotype D strains are global in distribution but are more frequently isolated in Europe [2].

The sexual cycle of *C. neoformans *is well defined and involves a bipolar mating system with two mating types: **a **and α [13]. The mating-type locus (*MAT*) is more than 100 kilobases (kb) in length and contains more than 20 genes [9,14,15]. Recombination is suppressed within *MAT *but is elevated in areas adjacent to the region, and extensive sequence divergence and rearrangements between the *MAT***a **and *MAT*α alleles have been described [14-16]. The majority of strains isolated from clinical and environmental sources are of the α mating type [13], and a serotype D strain of the α mating type is more virulent in mice than a congenic **a **strain [17]. In contrast, the congenic serotype A strains KN99**a **and KN99α exhibited no difference in murine virulence, although the latter strain more efficiently colonizes the central nervous system [12,18]. Further studies revealed that genomic regions outside the mating-type locus contribute to differences in virulence between **a **and α cells [19]. Large-scale genomic comparisons that may reveal sequences contributing to virulence differences between **a **and α strains have thus far been limited to the *MAT *locus [14,15,20].

Cells of *C. neoformans *are generally haploid, but it is possible to obtain relatively stable diploid strains through laboratory crosses and to identify naturally occurring AD hybrids that appear to result from the fusion of serotype A and D strains [9,21-24]. Most of the environmental and clinical AD isolates are diploid or aneuploid and contain alleles from both the serotype A and D genotypes [8]. However, recent studies indicate that some AD strains contain only a single mating-type locus (*MAT***a **or *MAT*α), either because of deletion of one allele or the complete absence of one of the *MAT *chromosomes [9,25,26]. These observations and the documented aneuploidy indicate that the genomes of the hybrid strains are unstable [9]. AD strains can be sterile or self-fertile, and in the latter case can produce filaments, basidia, and basidiospores to yield progeny that generally exhibit poor viability [9,25]. In one instance, the viable progeny from a self-fertile AD hybrid were found to be either diploid or aneuploid [9].

A number of molecular approaches have been used to investigate the genetic structure and epidemiologic relationships of *Cryptococcus *strains [4,8,27-34]. In particular, PCR fingerprinting and amplified fragment length polymorphism (AFLP) analysis revealed four major molecular types of *C. neoformans *[25,29,32,35]: VNI (AFLP1; serotype A), VNII (AFLP1A; serotype A), VNIII (AFLP3; AD hybrid), and VNIV (AFLP2; serotype D). Similarly, four molecular subtypes are found for *C. gattii*: VGI to VGIV [35], or AFLP groups 4 to 7 [8,32]. Multilocus sequence typing (MLST) has also been used in the phylogenetic analysis of a large number of isolates of *C. neoformans *and *C. gattii *[36-40]. This work provides insights into the population structure, geographic distribution, and evolutionary history of the species. For example, Litvintseva and coworkers [38,39] identified a unique group of serotype A isolates from Botswana (molecular subtype VNB) that included a significant proportion of fertile strains with the rare *MAT***a **mating type. Interestingly, those investigators went on to show that AD hybrids possessing the rare *MAT***a **allele clustered phylogenetically with serotype A isolates of the VNB subtype from Botswana. In contrast, AD hybrids with the more common *MAT*α allele clustered with serotype A isolates of the VNI molecular subtype that is found globally [40].

The genomes of *C. neoformans *and *C. gattii *have been characterized in terms of chromosome content and sequence. By karyotype analysis, the genome size of different species and varieties of *Cryptococcus *is estimated at 15 to 27 megabases (Mb) and chromosome number varies between 12 and 14 [31,32,41-45]. Loftus and coworkers [46] described the genome sequences for two serotype D strains, B3501A and JEC21, and these turned out to be about 19 Mb in size. The genome sequences of one serotype A strain (*C. neoformans *strain H99) and two serotype B strains (*C. gattii *strains WM276 and R265) have also been completed.

Comparative genome hybridization (CGH) is a rapid and cost-effective method to assess the presence, absence, or divergence of sequences in uncharacterized genomes by comparison with a reference genome. CGH has been applied to cancer cells, pathogenic and nonpathogenic bacteria and fungi, and other organisms [47-52]. In general, CGH circumvents the need to sequence multiple closely related genomes. For example, comparison of the genomes of two species in the fungal genus *Candida *(namely *C. albicans *and *C. dubliniensis*) confirmed the relatedness of the two species and identified a group of unique *C. albicans *genes that may contribute to virulence [53]. More recent CGH studies in *C. albicans *identified genome instability and revealed associations between aneuploidy, isochromosome formation, and azole resistance [50,51]. In this study, we used CGH to examine the genomes of selected strains of the A, D, and AD serotypes representing all of the molecular subtypes (VNI to VNIV) of *C. neoformans*. Specifically, we employed high-density tiling arrays to identify the global genome differences within and outside of the mating locus between *MAT***a **and *MAT*α strains, to map putative recombination sites in a genome resulting from a well characterized genetic cross, to distinguish the molecular subtypes of serotype A strains, and to identify the origins of chromosomes in selected AD hybrid strains.

## Results and discussion

### CGH detection of sequence divergence at the *MAT *locus

**Figure 1:**
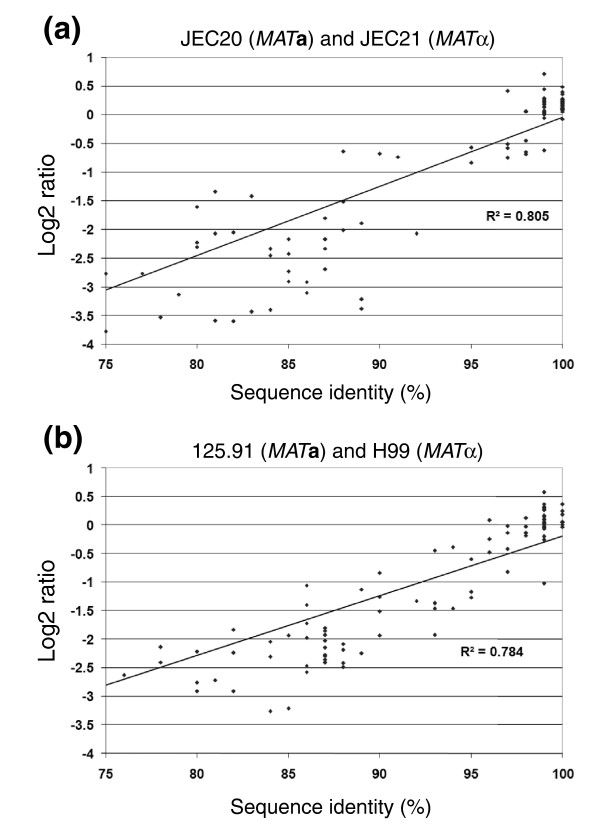
Comparisons of Log2 ratios and sequence identity for the *MAT***a** and *MAT*α loci of serotype A and D strains. Each data point represents the average Log2 ratio and sequence similarity of eight probes from a set of 400 base pair windows within 20 genes at the *MAT *locus. **(a) **Comparison of the *MAT *loci of the serotype D strains JEC20 (*MAT***a**) and JEC21 (*MAT*α). **(b) **Comparison of the *MAT *loci of the serotype A strains 125.91 (*MAT***a**) and H99 (*MAT*α). Note that the *MAT***a **region of JEC20 originated in strain NIH433 and genomic DNA of NIH433 was used for the hybridization experiment.

CGH signal ratios reflect the similarities or differences between reference and test genomes, and can detect the amplification, absence, or divergence of sequences. The CGH approach was applied to *C. neoformans *by first designing high-density tiling arrays of oligonucleotide probes with an average length and spacing of 50 base pairs (bp) and 44 bp, respectively, based on the genomes of strains JEC21 (serotype D) and H99 (serotype A; see Materials and methods, below). To establish a framework for identifying regions of difference in *Cryptococcus *genomes, CGH data collected with the tiling arrays was initially calibrated by comparing the Log2 ratios of the fluorescence intensity with the corresponding sequence identity for previously sequenced mating-type (*MAT*) regions of the test and reference genomes (Figure 1). The sequences of the approximately 100 kb *MAT *loci of representative serotype A (H99 and 125.91) and D (JEC21 and JEC20) strains were available for this analysis [15,46]. The sequences of the *MAT***a **and *MAT*α alleles were obtained from GenBank and the sequence identities for the coding regions of 20 genes in each of these loci were plotted against the corresponding Log2 ratios for the hybridization signals of the probes in the genes. That is, the average of the normalized Log2 ratios from every eight probes (spanning about 400 bp) covering each of the 20 *MAT *genes were compared with the corresponding genomic sequences for the *MAT***a **versus *MAT*α alleles. A correlation was found between sequence identity in the *MAT *regions and the Log2 ratios from hybridization signals for each of the comparisons (r^2 ^= 0.78 for the serotype A strains H99 and 125.91, and r^2 ^= 0.81 for the serotype D strains JEC21 and JEC20; Figure 1). The Log2 ratios for the hybridization signals ranged from 0.585 to -4.374, and from 0.971 to -4.382 in *MAT *regions of the serotype A and D strains, respectively. Based on these comparisons, Log2 ratios between -3.77 and 0.49 corresponded to sequence identities in the range of about 75% to 100% (Figure 1). We confirmed that these calibration values held true for randomly selected regions outside the *MAT *locus by comparing the identity between seven sequenced regions and hybridization probes (198 probes) against the Log2 ratio for those individual probes with the related strains NIH433 and NIH12 (described below, and data not shown).

In general, our observed correlations between the Log2 ratio and sequence identity are similar to CGH results for other organisms [53-56]. For example, Log2 ratios between -4.0 and 0.5 corresponded to sequence identities (probe/target identity) between 81% and 100% in *Campylobacter jejuni *[56]. Furthermore, CGH data for *Chlamydia trachomatis *showed a linear relationship between Log2 ratio and sequence identity between 75% and 99% [57]. In general, a Log2 ratio of ±1.0 is used in many CGH experiments to identify divergent (or deleted) and duplicated genes, representing a conservative threshold for divergent gene detection [55,56,58]. For our analysis, the observed correlation between sequence identity and Log2 ratio allowed us to predict whether specific regions in the genomes were divergent, deleted, and/or amplified. The use of these data for whole genome comparisons for serotype A, D, and AD strains are described below.

### Comparison of the *MAT*a and *MAT*α genomes of serotype D progenitor strains

Initially, we used CGH to compare the genomes of two serotype D progenitor strains, NIH433 (*MAT***a**) and NIH12 (*MAT*α), with the genome of the derived strain JEC21 (*MAT*α). JEC21 is commonly used in laboratory experiments and the genome of this strain has the best annotation of the sequenced cryptococcal genomes [46]. The strain was obtained from a series of back crosses (starting with NIH433 and NIH12) and was the *MAT*α representative of a congenic strain pair (with JEC20) used to examine the role of mating type in virulence [17,59]. NIH433 is an environmental isolate from pigeon droppings in Denmark, and NIH12 is a clinical isolate from a patient with osteomyelitis. The cross of NIH12 and NIH433 yielded two F_1 _strains B3501 (*MAT*α) and B3502 (*MAT***a**), and a cross of these strains yielded JEC20 (B-4476) [17,59]. JEC20 was subsequently used as the parent in a series of backcrosses to generate JEC21. Thus, approximately 50% of the overall genetic background of JEC21 should be derived from each of the NIH12 and NIH433 genomes.

**Figure 2:**
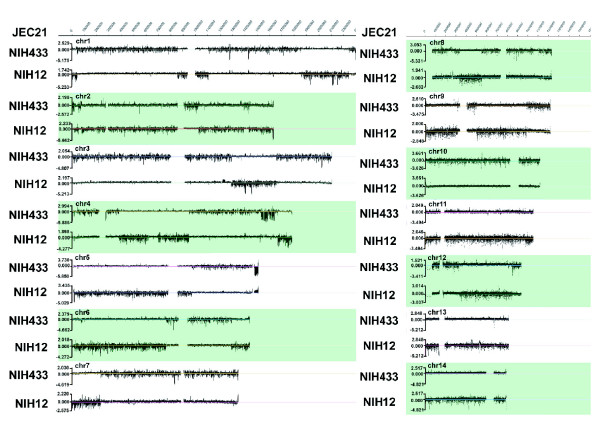
Genome hybridization to compare the progenitor strains NIH12 and NIH433 to the reference strain JEC21. Regions with higher variability in Log2 ratios in the test genomes are more divergent from the JEC21 sequence; regions with Log2 ratios close to zero have greater similarity (Additional data file 1). A reciprocal pattern of similar and divergent segments is found upon hybridization of genomes of NIH12 and NIH433 to the JEC21 array. The scale of chromosome coordinates for the JEC21 genome is indicated at the top of the figure, and gaps in the chromosomes represent putative centromeric regions [46]. The borders of segments are probable sites of recombination events that occurred during the mating of NIH12 and NIH433, and the subsequent backcrossing to obtain JEC21 (Additional data file 2) [59].

**Figure 3:**
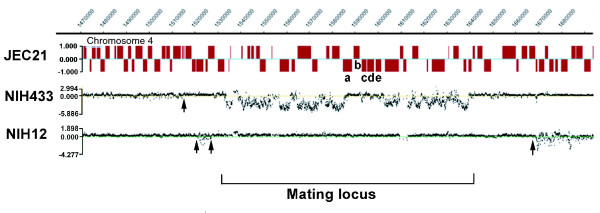
Sequence divergence and putative recombination sites at the *MAT *locus in serotype D strains. The chromosomal coordinates are shown at the top and the annotated genes in the region are indicated as boxes. Putative recombination sites are marked with arrows. Specific genes that are known to have high sequence similarity between the *MAT***a **and *MAT*α alleles [15], and that exhibit a corresponding Log2 ratio close to zero are labelled with letters: a, *RPO41 *(XM_570485.1; The Institute for Genomic Research [TIGR] locus tag CND05820); b, *BSP2 *(XM_570482.1; TIGR locus tag CND05830); c, *LPD1 *(XM_570114.1; TIGR locus tag CND05840); d, *CID1 *(XM_570548.1; TIGR locus tag CND05850); and e, *GEF1 *(XM_570546.1; TIGR locus tag CND05860). The gap in the hybridization signal for NIH12 and NIH433 centered on position 1,610,000 resulted from the presence of repeated sequences in this region that reduced probe density [15].

Initially, we hybridized the JEC21 array with DNA from the *MAT***a **strain NIH433 to compare the genomes of strains of opposite mating type. As expected, CGH showed that the *MAT***a **locus was divergent from the *MAT*α locus of JEC21 with Log2 ratios ranging from -5.27 to 1.24 for probes in the region. However, the analysis revealed an unexpected pattern of regions with either similar or divergent hybridization signals along 10 of the 14 chromosomes (Figure 2), in addition to the divergence observed at the *MAT *locus (Figures 1 and 3). Interestingly, each of these regions accounted for approximately 50% of the JEC21 genome, with regions of similarity accounting for about 8.73 Mb (50.6%) and divergent regions representing about 8.53 Mb (49.4%). Note that the JEC21 array did not contain the centromere sequences (or the rDNA cluster) and therefore covered about 17.3 Mb of the approximately 19 Mb genome [46] (Additional data file 1). These regions may represent the segments of the JEC21 genome originating from either NIH433 or NIH12, and the borders of the regions are likely to be sites of recombination events that occurred in the original cross. This idea was tested by hybridizing DNA from the *MAT*α parent NIH12 to the JEC21 array, and this analysis revealed a reciprocal pattern of similar and divergent regions as compared with the results with NIH433 (Figure 2). The averages and standard deviations (SDs) of Log2 ratios of the hybridization intensity of these regions along all chromosomes were consistent with the visual observations, in which regions of divergence generally have a higher SD than regions of similarity (Additional data file 1). This measure of divergence is illustrated quite clearly by signals from the *MAT *locus. That is, the SD of the *MAT***a **locus of NIH433 is 1.778 (average Log2 ratio of -1.632) upon comparison with the *MAT*α locus of JEC21. In contrast, the SD for the homologous *MAT*α locus of NIH12 is 0.229 (average Log2 ratio 0.090).

The putative recombination sites were observed in 10 of the 14 chromosomes and the number of these sites ranged from one on chromosome 7 to seven on chromosome 2 (Figure 2). Based on the current annotation, these sites occur in both intragenic and intergenic regions (Additional data file 2). Chromosomes 10, 11, 13, and 14 did not exhibit variability in Log2 ratios and different segments could not be distinguished. The SD of the hybridization ratios indicated that all of chromosome 10 may have originated from strain NIH12, with little or no contribution from NIH433. On the other hand, chromosomes 11, 13, and 14 are remarkably similar to NIH433 but divergent from NIH12, although one of the telomeres on chromosome 14 exhibited variability (Figure 2). Overall, these findings indicated that CGH with the JEC21 tiling array could detect sequence polymorphisms between strains of the same serotype and that these could be used to track recombination history. For *C. neoformans *and other fungal pathogens, the detection of recombination sites may have utility for mapping quantitative traits that contribute to virulence. Similar approaches have been used to examine breakpoints in the chromosomes of tumors and to characterize genetic diversity in *Saccharomyces cerevisiae *[47,60].

The CGH analysis also allowed an examination of the *MAT *regions of the serotype D strains NIH12 and NIH433 in comparison with that of JEC21. Specifically, we observed that the flanking regions of the *MAT *locus on chromosome 4 contained putative sites of recombination presumably from the cross of NIH12 and NIH433 (Figure 3) [59]. The observed sites are consistent with the previous identification of regions flanking the mating locus that are apparent hotspots of recombination [16]. In agreement, CGH revealed two potential recombination sites in a region of about 6 kb (chromosome 4: 1,525,471 to 1,531,510 and 1,514,930 to 1,520,952), as well as a third more distant site on the left side, and one site in a region approximately 27 kb to the right of the *MAT *locus (chromosome 4: 1,639,910-1,667,015; Figure 3). Within the mating-type locus, two divergently transcribed genes, *RPO41 *and *BSP2 *(chromosome 4: 1,585,174 to 1,591,759), were presumed to be involved in gene conversion because they are 99% identical between the two mating-type alleles in phylogenetic analyses [15,16]. Consistent with this finding, CGH yielded a Log2 ratio near zero, indicating high sequence similarity (-0.089 and 0.069 for NIH433 and NIH12, respectively; Figure 3). Three neighboring genes, *LPD1*, *CID1*, and *GEF1 *(chromosome 4: 1,592,529 to 1,602,117), share similar properties with *RPO41 *and *BSP2*, and Fraser and coworkers [15] described this area as a species-specific, syntenic region in the *MAT *locus.

Examination of the CGH data outside the *MAT *locus identified several candidate regions of difference between JEC21 and the progenitor strains NIH12 and NIH433. For example, two regions appeared to be present in single copy in NIH433 but duplicated in NIH12. One of these regions of approximately 20 kb (chromosome coordinates 1,218,400 to 1,239,200; average Log2 ratio of -0.062 in NIH433 and 0.997 in NIH12) is present on chromosome 3 and contains genes encoding several putative functions (PM-scl autoantigen, nicotinate-nucleotide diphosphorylase [carboxylating], phosphate transporter, and kynureninase). The other region of approximately 13 kb on chromosome 13 (chromosome coordinates 508,400-521,200) encodes putative functions including a metal transporter, a carboxylesterase, a (R, R)-butanediol dehydrogenase, and a formaldehyde dehydrogenase (glutathione; average Log2 ratio of 0.016 in NIH433 and 01.284 in NIH12). Additionally, an approximately 29 kb segment near the right telomere of chromosome 5 (encoding mostly hypothetical proteins) was highly divergent in NIH433 (average Log2 ratio of -3.232, SD of 1.496) and had an average Log2 ratio of 0.829 (SD of 0.382) in NIH12 (Figure 2). This region is part of an approximately 40 kb so-called 'identity island' in the genomes of the serotype A and D strains H99 and JEC21 [61]. The sequences of the region share 98.5% identity between the two genomes - a level that is approximately 10% higher than the average identity across the entire genomes. Kavanaugh and coworkers [61] found that the approximately 40 kb identity island was the apparent result of a nonreciprocal transfer event from a serotype A genome to a serotype D genome about 2 million years ago. They also surveyed 12 serotype D strains and found that ten (including NIH12) had the identity island that originated from the serotype A sequence and that two strains, NIH430 and NIH433, retained the original serotype D version of the sequence. Therefore, the analysis reported here for NIH12 and NIH433 matches the findings of Kavanaugh and coworkers [61], and thus demonstrates the utility of the CGH approach for detecting these types of sequence anomalies in *C. neoformans *genomes.

NIH433 and NIH12 are both virulent, but NIH433 requires more time than NIH12 to cause equal mortality when injected intravenously into mice [59]. In addition to the *MAT *locus, differences in genetic background were proposed to be important contributors to the virulence of these strains [59]. In this context, the differences observed for chromosomes 3, 5, and 13 or undetected polymorphisms (single nucleotide changes) could potentially influence virulence. For example, one region on chromosome 3 (2,022,000 to 2,024,000; Log2 ratio -3.161) contains a gene for a predicted O-acetyltransferase (CNC06920) that is deleted in NIH433, as confirmed by PCR (data not shown). O-acetyl substituents are found on the polysaccharide capsule that is the major virulence determinant of *C. neoformans*, and a mutant defective in O-acetylation was found to be hypervirulent [62,63].

### Genomic differences between serotype A strains representing three molecular subtypes

Serotype A strains of *C. neoformans *are responsible for the majority of clinical cases of cryptococcosis, particularly in AIDS patients, and three molecular subtypes (VNI, VNII, and VNB) have been identified by MLST and AFLP analyses [2,29,38,39]. Given that CGH detected variation within the serotype D strains, we next considered whether the same approach would detect genomic differences in serotype A strains of opposite mating type and different molecular subtypes. The majority of serotype A isolates have the VNI molecular subtype and the *MAT*α mating type. *MAT***a **strains are rare among clinical and environmental isolates compared with the prevalence of the *MAT*α mating type. In fact, *MAT***a **strains of serotype A were thought to be extinct or to exist as a vestigial, nonfunctional form until two clinical strains were identified: 125.91 from Tanzania [64] and IUM 96-2828 from Italy [65]. Strain 125.91 (VNI) was found to mate with a subset of *MAT*α serotype A strains, but was unable to mate with the reference strain H99 [12,64]. Recently, detailed MLST and AFLP analyses of a large population of serotype A strains from a global collection identified *MAT***a **isolates among the VNB molecular subtype that is uniquely present in Botswana; these isolates included strain Bt63, which can mate with H99 [38,39]. With the emerging view of the serotype A population in mind, we examined the genomes of two *MAT***a **strains (125.91 and Bt63), a VNII strain (WM626 [*MAT*α]), and a VNI strain (CNB7779 [*MAT*α]) reported to have a small genome [32]. For this analysis, we employed the tiling array based on the sequence of the VNI strain H99 and the corresponding chromosome numbers from the genome sequence assembly [61].

**Figure 4:**
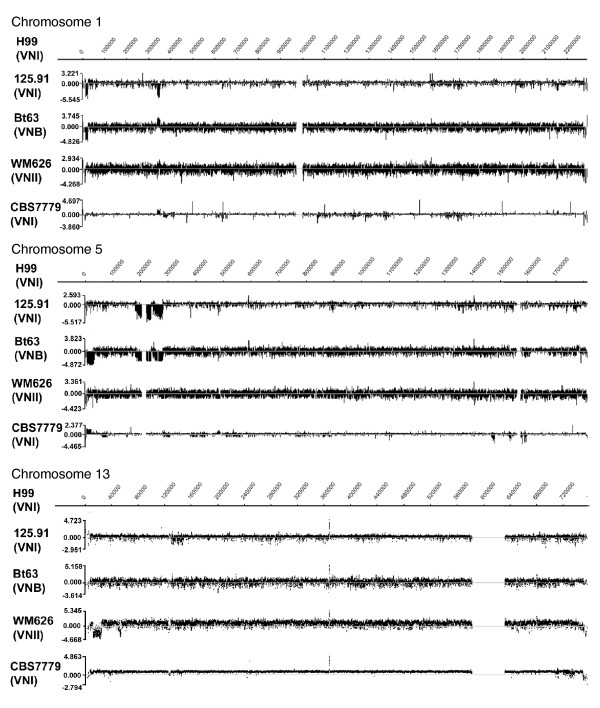
Variation in chromosomes 1, 5, and 13 for four serotype A strains. The DNA from the strains was hybridized to the array from the genome of strain H99, and gaps in the chromosomes represent the positions of repetitive sequences that represent putative centromeres or repeated elements in the *MAT *locus. The spikes in the Log2 ratios (for instance, for chromosome 1 of CBS7779) represent individual probes with high Log2 ratios (3 to 4); the sequences of these probes are present in single copy in the H99 genome but may be part of repetitive sequences in the other strains. For all of the chromosomes in all four strains, the hybridization data are shown in Additional data file 10, the Log2 ratios are listed in Table 1 and the regions of difference are listed in Additional data file 3.

Our analysis of strains 125.91 and Bt63 revealed that, as expected, the Log2 ratios of hybridization signals in the *MAT *locus region were highly variable and ranged from -4.345 to 0.445, and from -4.24 to 0.488, respectively. This indicates extensive sequence divergence between these *MAT***a **regions and the *MAT*α locus of H99 (Figures 1 and 4, and Additional data file 3). Note that the *MAT *locus is on chromosome 5 in the serotype A strain H99 used for the analysis of the *MAT *regions of the serotype A strains 125.91 and Bt63; *MAT *is on chromosome 4 in strain JEC21 (serotype D). The average Log2 ratios were -2.200 (SD 1.319) and -2.204 (SD 1.401) for 125.91 and Bt63, respectively. These results mirror the comparisons of the *MAT***a **and *MAT*α alleles for the serotype D strains presented above (Figures 1 to 3). Outside the *MAT *locus, the relative extent of divergence in hybridization signals between Bt63 and H99 (molecular subtypes VNB and VNI, respectively) versus 125.91 and H99 (both VNI) suggested that a higher level of genome variability exists between, versus within, molecular subtypes (Table 1). A large number of regions of difference were detected for both 125.91 and Bt63 relative to H99 (Additional data files 3 and 4). Several regions were also different between the two *MAT***a** strains. These include the region from 339,600 to 350,000 on chromosome 1 that appears to be absent from or highly divergent in 125.91 but potentially amplified in Bt63 (Additional data file 4) and an approximately 1.2 kb deletion (1,443,600 to 1,445,200) on chromosome 3 in Bt63 that was confirmed by PCR (Log2 ratio of -2.868 in the deleted region; data not shown). In addition, Bt63 appears to have a duplication of a region (1,776,000 to 1,786,400) on chromosome 5 that contains genes encoding putative myoinositol transporters. This observation is interesting because Xue and coworkers [66] recently showed that inositol stimulates mating in *C. neoformans*, and it is known that Bt63 mates more robustly with H99 than does 125.91 [64].

**Table 1:** Comparison of Log2 ratios and standard deviations for all 14 chromosomes of four serotype A strains

Chr	BT63 (VNB)		125.91 (VNI)		CBS7779 (VNI)^a^		WM626 (VNII)^a^	
	
	Average Log2 ratio	SD	Average Log2 ratio	SD	Average Log2 ratio	SD	Average Log2 ratio	SD
1	0.007	0.674	-0.019	0.438	-0.011	0.212	-0.014	0.634
2	0.013	0.634	0.007	0.312	-0.013	0.159	-0.019	0.637
3	0.034	0.600	0.020	0.272	-0.018	0.190	-0.011	0.660
4	-0.051	0.733	-0.002	0.329	-0.018	0.170	-0.105	0.778
5	-0.093	0.900	-0.097	0.623	-0.005	0.212	0.006	0.622
6	0.019	0.632	0.023	0.290	-0.035	0.263	-0.042	0.687
7	0.013	0.686	0.016	0.338	-0.029	0.258	-0.022	0.715
8	-0.048	0.773	0.020	0.323	-0.014	0.183	-0.032	0.724
9	0.031	0.625	0.018	0.292	-0.036	0.246	-0.026	0.643
10	0.050	0.612	0.033	0.310	-0.081	0.522	0.034	0.611
11	0.013	0.653	0.012	0.289	-0.019	0.212	-0.047	0.726
12	0.019	0.668	0.075	0.441	0.005	0.232	-0.085	0.827
13	0.025	0.627	-0.026	0.345	0.562	0.214	0.721	0.884
14	0.026	0.622	-0.006	0.328	-0.027	0.232	-0.060	0.685

^a^The log2 ratios for chromosome (Chr) 13 in these strains indicates a copy number greater than one. SD, standard deviation.

The genome variability between VNI and VNB strains revealed by CGH prompted us to compare the genome of the VNII strain WM626 with the H99 genome. VNII strains may represent up to 20% of the serotype A population worldwide, and WM626 is a clinical isolate from Sydney, Australia [29]. The results indicated that the WM626 genome is quite divergent from the H99 genome (Figure 4, Table 1, and Additional data file 3), suggesting considerable variation between the VNI and VNII subtypes (although a larger survey is needed). Compared with the results for 125.91 and Bt63, the CGH data for WM626 revealed a large number of highly divergent or deleted regions on 12 of the 14 chromosomes relative to the corresponding locations in Bt63 (Additional data file 4).

We also used CGH to examine the genome content of the clinical strain CBS7779 (VNI) from Argentina that was reported to have a small genome size as estimated by electrophoretic karyotype analysis [32]. Specifically, the genome size for CBS7779 was estimated to be 15 Mb, which is considerably smaller than the approximately 19 Mb genomes of the sequenced strains. We initially confirmed the published karyotype of CBS7779 (data not shown), and subsequent CGH analysis of the genome of this strain with H99 revealed Log2 ratios near zero for all of the chromosomes, suggesting a close relationship between the two strains (Figure 4 and Table 1). The similarities in the genomes were particularly evident in contrast to the results for the hybridizations with Bt63 and WM626 (Figure 4). These results suggested that CBS7779 shared most, if not all, of its genome with H99, and missing sequences that would account for the smaller estimated genome size were not found. This outcome may reflect the challenge in using electrophoretic karyotyping to measure genome size, particularly for strains that exhibit chromosome length polymorphisms that may result in co-migrating chromosomes. It is also possible that the genomes of these strains may contain different amounts of repetitive DNA. The comparison of the CBS7779 and H99 genomes did reveal a relatively small number of short regions of divergence. Two larger differences for CBS7779 relative to H99 included an apparent deleted or divergent region of 17 kb on chromosome 10 and an amplified region of 20 kb on chromosome 5 (Additional data file 4).

The evaluation of the serotype A strains also allowed a more detailed look at the 'identity island' discussed above. This region is on the left end of chromosome 5, based the assembled H99 genome sequence, and Kavanaugh and coworkers [61] found that several genes (The Institute for Genomic Research [TIGR] IDs: CNE05310, CNE05340, and CNE05350) in this region were present in H99 and 125.91, but not Bt63. CGH confirmed these findings and further revealed that other genes within the region (CNE05250, CNE05290, CNE05320, CNE05330, CNE05300, and CNE00020) were present in 125.91 (VNI) but absent in Bt63 (VNB; Additional data file 4). Interestingly, several genes within the region (CNE05310, CNE05340, CNE05350, CNE05320, and CNE05330) appeared to be duplicated in CBS7779 (VNI; Additional data file 4). These genes were also present in the VNII strain WM626, although a region of about 6 kb on the telomere proximal side appeared to be lost. For another region of high sequence identity between H99 (chromosome 10) and JEC21 (chromosome 13) [61], CGH revealed that three consecutive genes, namely CNM02580, CNM02590 and CNM02600, were conserved in all of the strains (H99, Bt63, 125.91, and CBS7779). However, CNM02570, which encodes a putative receptor protein near to the telomere, was present in H99, Bt63, CBS7779, and WM626, but not in 125.91. Kavanaugh and coworkers [61] found that repetitive elements were associated with the identity island on chromosome 5 and suggested that one of these (Cnl1) may have participated in translocation of the island in the serotype D strain. It is possible that these elements also contribute to the variability in these and other regions observed by CGH.

The CGH analysis also revealed an anomaly in the hybridization signals for chromosome 13 in strains CBS7779 and WM626, such that the Log2 ratio was above zero (0.562 and 0.721, respectively) across the entire chromosome (Table 1, Figure 4, and Additional data file 3). The simplest explanation for this is that chromosome 13 in these strains is present in more than one copy in some or all of the cells. Alternatively, duplicated chromosome 13 segments could reside elsewhere in the genome, but this is less likely, given that the entire chromosome exhibited an elevated Log2 ratio. Quantitative real-time PCR with three loci on chromosome 13 in strains H99, WM626, and CBS7779 supported the conclusion of an increased copy number in the latter two strains (Additional data files 5 and 6). Furthermore, replacement of the *APT1 *gene on chromosome 13 with a neomycin marker confirmed the presence of two copies of the gene in WM626, compared with the single copy found upon transformation of strain H99 (Additional data file 7). However, integration at the *APT1 *gene in strain CBS7779 yielded transformants with only the neomycin marker replacement of the gene. We hypothesize that instability at chromosome 13 in this strain may have resulted in the loss of one copy during the transformation process (see Additional data file 8). This idea is supported by quantitative real-time PCR data that confirmed a difference in copy number for chromosome 13 before and after transformation of the strain (Additional data files 5 and 6). Strains H99, WM626, and CBS7779 also exhibit phenotypic differences (for instance, in melanin formation) that could potentially result from the difference in chromosome content or the regions of divergence detected by CGH (Additional data file 9).

Other studies have documented genome anomalies for *C. neoformans*, including segmental duplications for serotype D strains [67] and chromosome length polymorphisms [32,68]. The possibility of elevated copy number for specific chromosomes in *C. neoformans *suggests that there is potentially greater genome variability among isolates than was previously appreciated. Chromosome copy number variation may have implications for differences in phenotypic properties between isolates. For example, variability in virulence has been observed for clinical isolates of serotype A and among A, D, and AD strains [69,70]. Additionally, *C. neoformans *exhibits phenotypic switching that influences the expression of virulence traits, interactions with the host immune system, the outcome of chronic infection and symptom development (specifically, intracranial pressure) [71]. It is possible that switching could result from changes in chromosome copy number, perhaps through a positive or negative influence on gene expression. In this regard, Torres and coworkers [72] recently showed that the gain of extra chromosomes in *S. cerevisiae *had a major influence on cellular processes, including gene expression, proliferation, metabolism, and protein turnover.

Taken together, the CGH data with selected serotype A strains revealed extensive variability, in agreement with the classification of the strains into different molecular subtypes. Specifically, variation in the Log2 ratios and SDs for each chromosome supports the grouping of H99 with the other VNI strains 125.91 and CBS7779 (lower SDs) and indicates divergence from the VNII strain WM626 and the VNB strain Bt63 (higher SDs; Table 1). Although we examined a small number of strains that may not be completely representative, the variable regions may define signature differences that could facilitate further characterization of molecular subtypes and epidemiologic studies. A wide variety of hypothetical genes and genes with predicted functions were present in the regions of difference, but no clear pattern of variation was observed, with the exception that variability was often associated with repetitive elements and/or present at subtelomeric regions (Additional data file 4). As described above, repeated sequences such as those associated with mobile genetic elements may contribute to genome instability and examples have been noted for *C. neoformans *[61]. For the telomeric and subtelomeric regions, the observed variability probably reflects rapid structural evolution of these regions in *C. neoformans*, similar to that observed at telomeric or subtelomeric regions of *S. cerevisiae*, *A. fumigatus*, *Magnaporthe grisea*, and many other organisms [47,73-77]. For example, CGH experiments with the genome of *Aspergillus fumigatus *strain Af293 as a reference revealed 2,557 genes that are absent or diverged in two additional strains of *A. fumigatus *and three closely related species, namely *A. clavatus*, *Neosartorya fischeri*, and *N. fennelliae *[75]. These absent or divergent genes exhibited a bias toward subtelomeric locations [74]. The identification of similar variable segments in *C. neoformans *may guide future analyses to assess whether genome variation contributes to the differences in virulence between serotype A strains [70]. Of course, the approach reported here would not detect regions that are present in the test genomes but not in H99.

### The chromosome complements of serotype AD hybrid strains

Clinical and environmental isolates of *C. neoformans *are normally haploid, and the diploid phase is a transient part of the sexual phase of the life cycle. Some clinical and environmental isolates possess a hybrid AD serotype and are presumed to result from natural fusions between A and D parental strains [78]. Fluorescence-activated cell sorting and PCR analyses also revealed that AD strains are diploid or aneuploid (>1*n *but <2*n*) [9,26]. We used the tiling arrays for the reference genomes of H99 (serotype A) and JEC21 (serotype D) to determine the utility of the CGH approach for characterizing hybrid strains. Three hybrid strains (KW5, CDC228, and CDC304) were chosen because they had previously been characterized with respect to virulence and they exhibited serotype-specific differences at some loci, including genes at the *MAT *locus [9].

**Figure 5:**
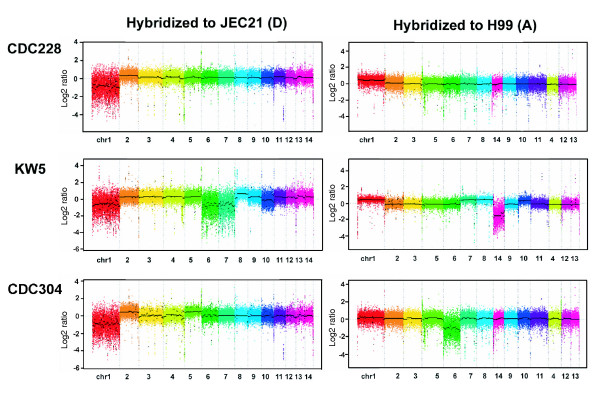
Hybridization analysis of three AD hybrid strains. The chromosome (chr) numbers listed at the bottom of each panel follow those of the reference genomes on the tiling arrays; for example, the chromosome numbers from JEC21 are used for the hybridization of DNA from each strain to the JEC21 array. Note that the JEC21 and H99 genomes are largely co-linear, but some of the homologous chromosomes have been assigned different numbers in the current genome assemblies [61]. Most of the chromosomes of the AD hybrid strains are represented by copies from both A and D genomes. However, the hybridization signals indicate that chromosome 1 is only represented by sequences from a serotype A genome in all three strains. Similarly, chromosomes 5, 6, and 7 (and chromosome 14 in KW5) are represented by sequences from only one of the serotypes (either A or D). The average Log2 ratios and standard deviations for all of the chromosomes are listed in Additional data file 6, and PCR-RFLP confirmation for selected chromosomes is shown in Figure 6.

Hybridization of DNA from the AD strains to the JEC21 and H99 arrays suggested that most chromosomes (chromosomes 2 to 4 and 9 to 14 [numbers based on the JEC21 genome]) were represented by copies from both the A and D genomes because the average Log2 ratios were close to zero (Figure 5; Additional data file 10). This observation was supported by the finding that other chromosomes of the A and D genomes were not equally represented in the three AD hybrid strains (Figure 5 and Additional data file 10). Specifically, Log2 ratios for chromosome 1 in the AD strains ranged from -1 to -4 upon hybridization to the JEC21 array, suggesting the absence of sequences of chromosome 1 originating from a D genome, whereas the ratios for hybridization to the H99 array were 0.5 to 1, suggesting the presence of one or two copies of chromosome 1 from the parental A genome. Note that the VN molecular subtype of the parental strains for the hybrids is not known, and the variation in the Log2 ratios may reflect sequence divergence between H99 and the serotype A parent. The average Log2 ratios for each chromosome of the three strains are listed in Additional data file 10. Overall, the results indicated that chromosome 1 of KW5, CDC208, and CDC304 originated from an A genome, suggesting that the D genome version of chromosome 1 may have been lost. Similarly, based on the Log2 ratios, chromosomes 6 and 7 of KW5 may have only a serotype A version, chromosome 8 of KW5 appears to be from a D genome, and chromosome 5 of CDC304 may have only a serotype D version.

**Figure 6:**
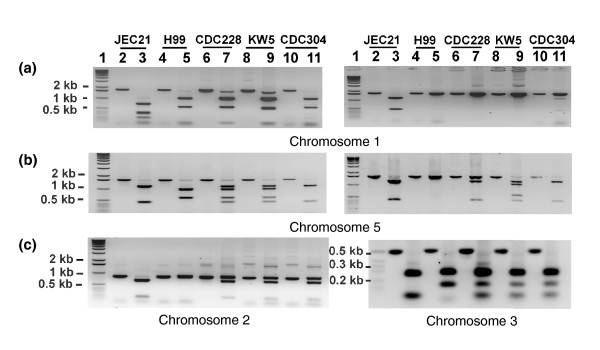
PCR-RFLP confirmation of the presence of serotype-specific chromosomes in three AD hybrid strains. Agarose gels are shown in which lane 1 for each contains size markers (1 kilobase [kb] ladder); lanes 2, 4, 6, 8, and 10 contain undigested PCR fragments; and the remaining lanes contain the same fragments after restriction enzyme digestion. **(a) **Digestion of PCR fragments (primers CNA01230 F/R) from chromosome (chr) 1 with *Ava*I (left panel) or *Stu*I (right panel). **(b) **Digestion of PCR fragments (primers CNE04380 F/R) from chromosome 5 with *Taq*I (left panel) or *Hind*III (right panel). **(c) **Digestion of a PCR fragment (primers CNB01970 F/R) from chromosome 2 with *Nde*I (left panel) and a fragment (primers acidphos F/R) from chromosome 3 with *Taq*I (right panel).

The predictions about the presence of specific chromosomes were tested by PCR-RFLP analysis of selected regions. Specifically, tests were performed with chromosome 5 as a representative chromosome only from serotype D in CDC304 and with chromosome 1 as representative of a chromosome only from serotype A in all three strains. For comparison, chromosomes 2 and 3 were included as examples of chromosomes that were present from both serotype A and D parents (Figure 5). Initially, PCR-RFLP analysis of a region in the JEC21 gene CNE04380 on chromosome 5 that was conserved between the A and D genomes confirmed that KW5 and CDC228 have both a serotype A and serotype D copy of chromosome 5, whereas CD304 only has the serotype D sequence of this region (Figure 6). Digestion with a different enzyme confirmed these results and revealed that the serotype A copy in KW5 has a different RFLP pattern from that in CDC228, most likely due to restriction site polymorphisms. Parallel PCR-RFLP analyses for chromosomes 2 and 3 confirmed the CGH prediction that the AD hybrids contained these chromosomes from both the A and D genomes.

Chromosome 1 in the AD hybrids was also examined by PCR-RFLP analysis of a segment of the conserved JEC21 gene CNA01230 (Figure 6). This analysis initially indicated that all three strains had only a serotype A version of chromosome 1, although closer examination revealed faint additional bands from strain CDC304 that could result from incomplete digestion, sequence polymorphisms at the site, or the presence of the serotype D version of chromosome 1. Restriction digests with additional enzymes suggested that CDC304 might have a serotype D version of chromosome 1 present at a low level (Figure 6). One possibility was that strain CDC304 contained a mixed population of cells in which the majority had a serotype A copy of chromosome 1 and a minority also contained a copy from serotype D. To test this hypothesis, we isolated and analyzed four different colonies of CDC304 and found that the cells in three colonies had only a serotype A version of chromosome 1. The other colony contained cells in which the serotype A and D versions of chromosome 1 were present in approximately equal abundance (data not shown). We therefore hypothesize that most cells in strain CDC304 have lost the serotype D copy of chromosome 1, but that a minor population retains this chromosome. Thus, CDC304 may still be in the process of losing chromosomes from the original A and D parents, and this observation is consistent with the genome instability observed for AD hybrid strains [9].

The AD hybrid strains of *Cryptococcus *examined in this study showed that chromosomes of both A and D serotypes are not equally represented in each AD hybrid strain. That is, specific chromosomes were represented by sequences from only one serotype. Although, we have not determined the copy number for chromosomes that are represented by a single serotype sequence, the possibility exists that these chromosomes are present in two copies because the average Log2 ratios were about 0.4. It is notable that all three AD strains preferentially contained chromosome 1 sequences from the serotype A parental strain but not from the D strain. To strengthen the conclusion that chromosome 1 from serotype A is preferentially retained in hybrids, we obtained 16 additional AD hybrid strains and examined the origin of chromosome 1 using the RFLP-PCR method (Additional data file 11). We found that 11 possessed chromosome 1 sequences only from serotype A and the other five had copies of chromosome 1 from both A and D parents. These results agree with those of Nakamura and coworkers [79] and Okabayashi and colleagues [80], who used *CAP59 *gene sequences from chromosome 1 to examine phylogenetic relationships between serotypes. They found that the three AD hybrid strains that they tested grouped with serotype A strains, suggesting that these strains also had only the serotype A allele of *CAP59*. Similarly, Xu and coworkers [24] found evidence for loss of heterozygosity in AD hybrid strains using MSLT analysis with four genes and identified strains that cluster with serotype A strains as well as others that lacked consistent grouping with one serotype.

The emerging picture of frequent loss of heterozygosity in hybrid strains and the retention of specific chromosomes raises questions about the mechanisms of loss and retention. It is possible that chromosome 1 of serotype A carries a gene or genes that confer a selective advantage relative to the serotype D version of the chromosome, and/or that the latter chromosome has a disadvantageous combination of genes. In this case, hybrids that spontaneously lose chromosome 1 from serotype A might be at a selective disadvantage. It is also possible that chromosome 1 from the serotype D parent has an inherent defect in replication or transmission, such that it is preferentially lost or that incompatibility exists for some chromosomes. The mechanisms underlying these patterns of chromosome content, and possible relevance for virulence, remain to be investigated. It is interesting, however, that the AD strains are clinical isolates, which raises the possibility that retention of chromosome 1 from the serotype A genome (serotype A strains are known to be more virulent) contributes a selective advantage in the mammalian host environment.

A set of genes or regions in the AD hybrids have been amplified and compared previously [9]. We examined the CGH data for these sequences, and the Log2 ratios are consistent with the interpretations of chromosome content (data not shown). The three AD strains have also been tested previously for virulence in comparison with strain H99 [9]. These assays indicated that the AD hybrids are less virulent than H99 and differ from each other. Specifically, infected mice succumbed to H99 by day 25, to KW5 by day 100, and to CDC228 or CDC304 by day 150. It is known that serotype A strains are generally more virulent that serotype D strains, although strain variation exists in both groups [69,70]. Differences in chromosomal content could potentially influence virulence through the contributions of specific alleles, such that a greater content of the A genome (as found in KW5) may result in enhanced virulence. Of course, other explanations are possible, including the accumulation of mutations in key virulence traits in the less virulent AD strains and epigenetic phenomena. Barchiesi and coworkers [69] suggested that the presence of the serotype A, *MAT*α mating type allele in either haploid or diploid (aneuploid) strains is correlated with virulence, whereas the *MAT***a **in serotype A or *MAT*α allele in serotype D is associated with moderate or no virulence. In this regard, Lengeler and colleagues [9] showed that KW5 is heterozygous for the mating-type locus with the serotype D *MAT***a **and the serotype A *MAT*α loci present. In contrast, the CDC228 and CDC304 strains have the *MAT*α locus from a serotype D parent and appear to have the *MAT***a **locus from a serotype A parent. Therefore, the contributions of mating type to virulence are unclear, with contributions likely both from specific alleles and variable chromosome complements in the AD hybrids.

## Conclusion

The CGH data presented here corroborated the aneuploidy of AD hybrids and identified preferential chromosome retention in some strains. Coupled with the discovery of copy number differences for chromosome 13 in two serotype A strains, these results identified an unexpected level of genome variation in *C. neoformans*. This extensive genome variation is in contrast to previous reports of extensive clonality in a large number of isolates representing the environmental population [81]. This may partly reflect one of the advantages of CGH over AFLP analysis, in that the latter method is typically unable to identify chromosome or segmental duplication. Many fungi have a remarkable tolerance to variability in chromosome content, and this may be advantageous for adaptation to different environmental niches. For example, aneuploidy is common in *C. albicans*, and aneuploidy and isochromosome formation can contribute to drug resistance [50,51]. Additionally, changes in chromosome copy number can influence virulence [82]. Given these findings, one can envisage more detailed CGH experiments to determine whether genome variation contributes to the phenotypic and virulence differences between strains, mutants, and switch variants. Variation may also occur during passage through an animal or during antifungal drug treatment. In combination with physical mapping [83] and sequencing, CGH will also allow detailed characterization of emerging strains of clinical significance and novel populations such as the unusual VNB isolates that appear to be restricted to Botswana [39]. In this light, we are also using the sequenced genomes of *C. gattii *strains and CGH to investigate genome variability in strains from the outbreak that is ongoing on Vancouver Island [7,84]. The combined view of genome variability in *C. neoformans *and *C. gattii *may thus provide insights into the mechanisms of genome microevolution in these pathogens.

## Materials and methods

### Strains, genomic DNA extraction and array hybridization

Additional data file 12 lists the strains used in this study. The strains were maintained on yeast extract, peptone, dextrose medium (YPD; Difco, Sparks, MD, USA) and the genomic DNA for hybridization experiments was isolated as previously described [85]. The genome sequences of strain JEC21 [46] and strain H99 were obtained from public databases [86,87]. The assembled sequences were used by NimbleGen Systems, Inc. (Madison, WI, USA) [88] to design and manufacture high-density oligonucleotide genomic arrays. The design for the arrays was the same for each genome, with oligonucleotide probes that cover all 14 chromosomes for each genome tiled at an average interval spacing of 44 bp on one strand. The average length of the probes was 50 bp (range 45 to 85 bp) and the average melting temperature (Tm) of the probes on each array was 76°C. The number of oligonucleotide probes for each genome were as follows: 386,279 for H99 and 380,236 for JEC21. The probes for each array were designed to uniquely match a single sequence in the genome, and highly repetitive centromeric regions and the rDNA repeat cluster were not included. In H99, a region of the mating locus (chromosome 5: 203,813 to 220,976) was considered repetitive and not included on the array. The comparative standard operation procedures of NimbleGen Systems, Inc. were followed for hybridization (42°C), array scanning, and data acquisition, as described previously [60]. Genomic DNA from the test strains was labelled with Cy3 and that of reference strains with Cy5. The data were expressed as Log2 ratios of Cy3/Cy5 fluorescence intensity. The arrays are available from NimbleGen Systems, Inc.

### Data analysis

The data were extracted from scanned array images using NimbleScan 2.0 software (NimbleGen Systems, Inc.) and were provided to us as an initial DNA segmentation analysis of the normalized data. This analysis included a window averaging step, in which the probes that fell into a defined base pair window size were averaged using the Tukey biweight mean. The adjacent windows were averaged to reduce the size of the dataset and the noise in the data. In the present study, the data were analyzed using 400, 800, and 2,000 bp windows for averaging; these sizes represent 10, 20, and 50 times the length of an average probe. In most cases, a 400 bp window was used for the analysis. The segmentation of the averaged log2 ratio data was determined based on a circular binary segmentation algorithm [89]. We also examined the data for each individual probe in the analyses shown in Figure 1 and Additional data file 4. The sample key for the raw hybridization data is provided in Additional data file 13 and the actual data files are available on our website [90].

The CGH results were viewed and analyzed as GFF files with SignalMap (NimbleGen Systems, Inc.) using 400, 800, and 2,000 bp windows for averaging. The data for the *MAT *loci (Figure 1) were analyzed using Log2 ratios from segmentations based on a 400 bp window and the nucleotide sequence identity for the corresponding region of available homologous sequences from 20 genes in the loci. The sequences of the *MAT *loci were obtained from GenBank, and sequence identity was determined from alignments with the BioEdit Sequence Alignment Editor [91]. The accession numbers for the sequences are as follows: serotype A strains H99 (*MAT*α, AF542529) and 125.91 (*MAT***a**, AF542528), and serotype D strains JEC21 (*MAT*α, AF542531) and JEC20 (*MAT***a**, AF542530). The relationship between Log2 ratio values and sequence identity was examined by linear regression analysis in Excel.

### Verification of CGH results by PCR amplification and RFLP analysis

Selected regions identified by CGH as candidates for deleted, duplicated, or divergent sequences were examined by PCR amplification and sequence analysis. PCR amplification from genomic DNA was performed as described by Hu and Kronstad [85], with primers designed from the sequences flanking each predicted region of difference, so that the amplified fragment spanned the region. The primers were obtained from Invitrogen (Burlington, Ontario, Canada) and their sequences are listed in Additional data file 14. PCR-amplified DNA fragments were either sequenced using the dideoxy chain-termination method or digested with selected restriction enzymes in the case of AD hybrids. Primers for the analysis of AD hybrid strains were designed to target highly conserved regions in the genome to ensure that the primers would bind to both the serotype A and D alleles in these strains. These conserved regions were identified by aligning the sequences from H99 (representing serotype A) and JEC21 (representing serotype D) with BioEdit [91].

## Abbreviations

AFLP, amplified fragment length polymorphism; bp, base pairs; CGH, comparative genome hybridization; kb, kilobases; Mb, megabases; MLST, multilocus sequence typing; PCR, polymerase chain reaction; RT, reverse transcription; SD, standard deviation; TIGR, The Institute for Genomic Research.

## Authors' contributions

GH, IL and JWK conceived and designed the study. FSD, GH, JWK, IL, and JES analyzed the data and wrote the paper. AS analyzed the CGH data for the *MAT *loci.

## Additional data files

The following additional data are available. Additional data file 1 is a table of Log2 ratios of divergent and conserved segments of the JEC21 genome relative to the genomes of the progenitor strains NIH12 and NIH433. Additional data file 2 is a table of the regions of putative recombination sites across all of the chromosomes in the JEC21 genome. Additional data file 3 is a figure of the variation in the genomes of strains representing the three molecular subtypes within the A serotype of *C. neoformans*. Additional data file 4 is a table of regions of difference in the genomes of four serotype A strains compared with the sequenced genome of strain H99. Additional data file 5 is a table of the quantitative RT-PCR analysis of gene copy number relative to the H99 genome. Additional data file 6 is a table of the quantitative RT-PCR analysis of gene copy number relative to the JEC21 genome. Additional data file 7 is a figure showing the replacement of a gene on chromosome 13 to examine copy number. Additional data file 8 describes the genomic hybridization analysis of transformants carrying a replacement at the *APT1 *gene on chromosome 13 in strains H99, CBS7779, and WM626; this file also presents an estimation of relative copy number by quantitative real-time PCR of selected markers on chromosome 13 in strains H99, JEC21, CBS7779, and WM626. Additional data file 9 is a figure showing the phenotypic differences between strains H99, CBS7779, and WM626. Additional data file 10 is a table of the average Log2 ratios of the chromosomes in three AD hybrid strains upon hybridization to the tiling arrays of the JEC21 and H99 genomes. Additional data file 11 is a figure showing an RFLP-PCR analysis of the origin of chromosome 1 in 16 AD hybrid strains. Additional data file 12 is a table list of *Cryptococcus neoformans *and *C. gattii *strains. Additional data file 13 is a sample key for the CGH data. Additional data file 14 is a table list of primer sequences. Additional data file 15 contains the figure legends for the figures presented in Additional data files 3, 7, 9 and 11.

## Supplementary Material

Additional data file 1 Presented is a table of Log2 ratios of divergent and conserved segments of the JEC21 genome relative to the genomes of the progenitor strains NIH12 and NIH433. Click here for file

Additional data file 2 Presented is a table of the regions of putative recombination sites across all of the chromosomes in the JEC21 genome. Click here for file

Additional data file 3 Presented is a figure of the variation in the genomes of strains representing the three molecular subtypes within the A serotype of *C. neoformans*. Click here for file

Additional data file 4 Presented is a table of regions of difference in the genomes of four serotype A strains compared with the sequenced genome of strain H99. Click here for file

Additional data file 5 Presented is a table of the quantitative RT-PCR analysis of gene copy number relative to the H99 genome. Click here for file

Additional data file 6 Presented is a table of the quantitative RT-PCR analysis of gene copy number relative to the JEC21 genome. Click here for file

Additional data file 7 Presented is a figure showing the replacement of a gene on chromosome 13 to examine copy number. Click here for file

Additional data file 8 Presented is a document describing the genomic hybridization analysis of transformants carrying a replacement at the *APT1 *gene on chromosome 13 in strains H99, CBS7779, and WM626. This file also presents an estimation of relative copy number by quantitative real-time PCR of selected markers on chromosome 13 in strains H99, JEC21, CBS7779, and WM626. Click here for file

Additional data file 9 Presented is a figure showing the phenotypic differences between strains H99, CBS7779, and WM626. Click here for file

Additional data file 10 Presented is a table of the average Log2 ratios of the chromosomes in three AD hybrid strains upon hybridization to the tiling arrays of the JEC21 and H99 genomes. Click here for file

Additional data file 11 Presented is a figure showing an RFLP-PCR analysis of the origin of chromosome 1 in 16 AD hybrid strains. Click here for file

Additional data file 12 Presented is a tabulated list of *Cryptococcus neoformans *and *C. gattii *strains. Click here for file

Additional data file 13 Presented is a sample key for the CGH data. Click here for file

Additional data file 14 Presented is a tabulated list of primer sequences. Click here for file

Additional data file 15 Presented are the figure legends for the figures presented in Additional data files 3, 7, 9 and 11. Click here for file
