# Supplementary material for: Comparative hybridization reveals extensive genome variation in the AIDS-associated pathogen Cryptococcus neoformans
Source: Genome Biol. 2008 Feb 22;9(2):R41. doi: 10.1186/gb-2008-9-2-r41 (PMC2374700; doi:10.1186/gb-2008-9-2-r41)
Supplement: Additional data file 1 — Presented is a table of Log2 ratios of divergent and conserved segments of the JEC21 genome relative to the genomes of the progenitor strains NIH12 and NIH433. [file gb-2008-9-2-r41-S1.doc]

| **Additional data file 1. Log2 ratios of divergent and conserved segments of the JEC21 genome relative to the genomes of the progenitor strains NIH12 and NIH433.** | | | | | | |
| --- | --- | --- | --- | --- | --- | --- |
| **Chr.** | **Coordinates** | **Divergent strain** | **Avg. Log2 (NIH433)** | **SD of NIH433** | **Avg. Log2 (NIH12)** | **SD of NIH12** |
| 1 | 1 - 43532 | NIH12 | -0.032 | 0.367 | -0.593 | 0.837 |
| 1 | 43576 - 856009 | NIH433 | 0.001 | 0.381 | 0.07 | 0.181 |
| 1 | 856053 - 1104183 | NIH12 | 0.073 | 0.176 | -0.015 | 0.42 |
| 1 | 1104402 - 1856406 | NIH433 | -0.015 | 0.484 | 0.098 | 0.207 |
| 1 | 1856450 - 2299466 | NIH12 | 0.04 | 0.337 | -0.121 | 0.608 |
| 2 | 1 - 14866 | NIH433 | 0.066 | 0.504 | -0.205 | 0.386 |
| 2 | 14910 - 59776 | NIH12 | -0.11 | 0.362 | -0.307 | 0.441 |
| 2 | 59820 - 79744 | NIH433 | -0.027 | 0.402 | -0.109 | 0.367 |
| 2 | 79788 - 905375 | NIH12 | 0.051 | 0.19 | -0.01 | 0.412 |
| 2 | 905419 - 1023729 | NIH433 | 0.017 | 0.343 | 0.074 | 0.164 |
| 2 | 1023773 - 1388827 | NIH12 | 0.07 | 0.182 | -0.021 | 0.422 |
| 2 | 1388871 - 1433328 | NIH433 | -0.009 | 0.356 | 0.067 | 0.208 |
| 2 | 1433372 - 1632054 | NIH12 | 0.02 | 0.194 | -0.219 | 0.658 |
| 3 | 1 - 1218953 | NIH433 | -0.003 | 0.378 | 0.08 | 0.187 |
| 3 | 1218997 - 1656795 | NIH12 | 0.071 | 0.257 | -0.037 | 0.652 |
| 3 | 1656839 - 2105433 | NIH433 | -0.049 | 0.466 | 0.016 | 0.201 |
| 4 | 1 - 381958 | NIH433 | -0.156 | 0.766 | 0.028 | 0.202 |
| 4 | 382002 - 943451 | NIH12 | 0.066 | 0.178 | 0.015 | 0.414 |
| 4 | 943495 - 1668854 | NIH433 | -0.281 | 1.023 | 0.094 | 0.202 |
| 4 | 1668898 - 1782959 | NIH12 | -0.042 | 0.236 | -0.412 | 0.75 |
| 5 | 1 - 969309 | NIH12 | 0.059 | 0.179 | -0.062 | 0.489 |
| 5 | 969423 - 1507393 | NIH433 | -0.176 | 0.904 | 0.109 | 0.28 |
| 6 | 1 - 75987 | NIH12 | -0.034 | 0.209 | -0.273 | 0.433 |
| 6 | 76020 - 1291564 | NIH433 | 0.044 | 0.288 | 0.014 | 0.382 |
| 6 | 1291702 - 1431588 | NIH12 | 0.006 | 0.197 | -0.127 | 0.406 |
| 6 | 1431632 - 1438769 | NIH433 | 0.178 | 0.745 | 0.142 | 0.526 |
| 7 | 1 - 230738 | NIH12 | 0.014 | 0.199 | -0.072 | 0.403 |
| 7 | 230827 - 1346622 | NIH433 | -0.005 | 0.421 | 0.077 | 0.2 |
| 8 | 1 - 318879 | NIH433 | 0.058 | 0.435 | 0.024 | 0.21 |
| 8 | 318923 - 529999 | NIH12 | 0.045 | 0.175 | -0.073 | 0.42 |
| 8 | 530208 - 1194145 | NIH433 | -0.018 | 0.448 | 0.043 | 0.199 |
| 9 | 1 - 300354 | NIH12 | 0.033 | 0.193 | -0.108 | 0.46 |
| 9 | 300419 - 487358 | NIH433 | 0.02 | 0.456 | 0.097 | 0.209 |
| 9 | 487446 - 954505 | NIH12 | 0.047 | 0.174 | -0.009 | 0.369 |
| 9 | 954549 - 1168552 | NIH433 | -0.068 | 0.412 | -0.02 | 0.217 |
| 10 | whole chromosome | NIH433 | -0.014 | 0.397 | 0.055 | 0.2 |
| 11 | whole chromosome | NIH12 | 0.036 | 0.191 | -0.083 | 0.455 |
| 12 | 1-63228 | NIH12 | -0.945 | 0.347 | -0.597 | 0.520 |
| 12 | 63228-318097 | NIH433 | -0.026 | 0.379 | 0.078 | 0.211 |
| 12 | 318889-820236 | NIH12 | 0.051 | 0.184 | -0.0251 | 0.417 |
| 12 | 821737-900462 | NIH433 | -0.163 | 0.428 | -0.148 | 0.210 |
| 13 | whole chromosome | NIH12 | 0.042 | 0.206 | -0.087 | 0.584 |
| 14 | whole chromosome | NIH12 | 0.055 | 0.209 | -0.063 | 0.474 |

Cells marked in yellow indicate elevated standard deviations (SD) indicating sequence divergence.
