# Supplementary material for: Comparative hybridization reveals extensive genome variation in the AIDS-associated pathogen Cryptococcus neoformans
Source: Genome Biol. 2008 Feb 22;9(2):R41. doi: 10.1186/gb-2008-9-2-r41 (PMC2374700; doi:10.1186/gb-2008-9-2-r41)
Supplement: Additional data file 2 — Presented is a table of the regions of putative recombination sites across all of the chromosomes in the JEC21 genome. [file gb-2008-9-2-r41-S2.doc]

| **Additional data file 2. Regions of putative recombination sites across all of the chromosomes in the JEC21 genome** | | |
| --- | --- | --- |
| **Chr.** | **Coordinates** | **GenBank accession and predicted function of gene at site or genes flanking the site** |
| 1 | 41544-42229 | IGS^a^ between XM_566442 and XM_566443 |
|  | 854226-854402 | IGS between transcriptional activator gcn5 (XM_566649) and hst3 protein (XM_566762) |
|  | 1102595-1103156 | IGS between ER organization and biogenesis-related protein (XM_566769) and choline-phosphate cytidylyltransferase (XM_566767) |
|  | 1856357 - 1858480 | XM_566911 CAP 64 gene product, related |
|  | 2242820-2244212 | IGS between XM_567193 and XM_567194 |
| 2 | 18945-19362 | IGS between XM_568753 and exonuclease XM_568754 |
|  | 53942-55579 | IGS between XM_568759 and XM_568760, 1-aminocyclopropane-1-carboxylate deaminase |
|  | 78213-78563 | XM_568769 nuclear pore protein seh1 |
|  | 1022498-1025605 | XM_568915 hypothetical protein |
|  | 1388816 - 1389906 | XM_568980 40s ribosomal protein s3ae-a (s1-a), putative |
|  | 1433334 - 1434459 | XM_569252 structural constituent of ribosome |
| 3 | 1290459 - 1292331 | XM_569673 di-, tri-valent inorganic cation transporter, putative |
|  | 1653913-1655187 | IGS between acid phosphatase XM_569758 and XM_569760 |
| 4 | 381797 - 382321 | XM_570426 glutathione transferase, putative |
|  | 946121-948190 | XM_570561 chitin deacetylase-like mannoprotein MP98 |
|  | 1514930-1515427 | XM_570648 hypothetical protein |
|  | 1520952-1521916 | XM_570500 conserved hypothetical protein |
|  | 1525471-1527411 | XM_570478 conserved hypothetical protein |
|  | 1531510-1533619 | XM_570688 and XM_570126, pheromone alpha |
|  | 1639410-1639910 | XM_570536 oxidoreductase |
|  | 1667015-1668986 | XM_570098 hypothetical protein |
| 5 | 969333 - 970610 | XM_570908 expressed protein |
| 6 | 760035 - 762572 | XM_571362 ubiquitin carboxyl-terminal hydrolase 14, putative |
|  | 1291574 - 1293168 | XM_571697 pyruvate dehydrogenase e1 component alpha subunit, mitochondrial precursor, putative |
| 7 | 230803 - 231836 | XM_571814 structural constituent of ribosome, putative |
| 8 | 320224-320523 | XM_572454 2-nitropropane dioxygenase |
|  | 530139 - 531563 | XM_572387 hypothetical protein |
| 9 | 300685-301936 | XM_572691 hypothetical protein |
|  | 478313-479884 | XM_572964 vacuole protein |
|  | 954506 - 956980 | XM_572888 signal transducer, putative |
| 12 | 318537-319505 | XM_568014 phospholipid:diacylglycerol acyltransferase |
|  | 821149-822340 | IGS between XM_568174, conservative hypothetical protein and XM_568175, cytoplasm protein |

^a^ IGS indicates the intergenic space between the flanking genes indicated by accession numbers.
