# Supplementary figures and images for: Comparative hybridization reveals extensive genome variation in the AIDS-associated pathogen Cryptococcus neoformans
Source: Genome Biol. 2008 Feb 22;9(2):R41. doi: 10.1186/gb-2008-9-2-r41 (PMC2374700; doi:10.1186/gb-2008-9-2-r41)

## Slide 1
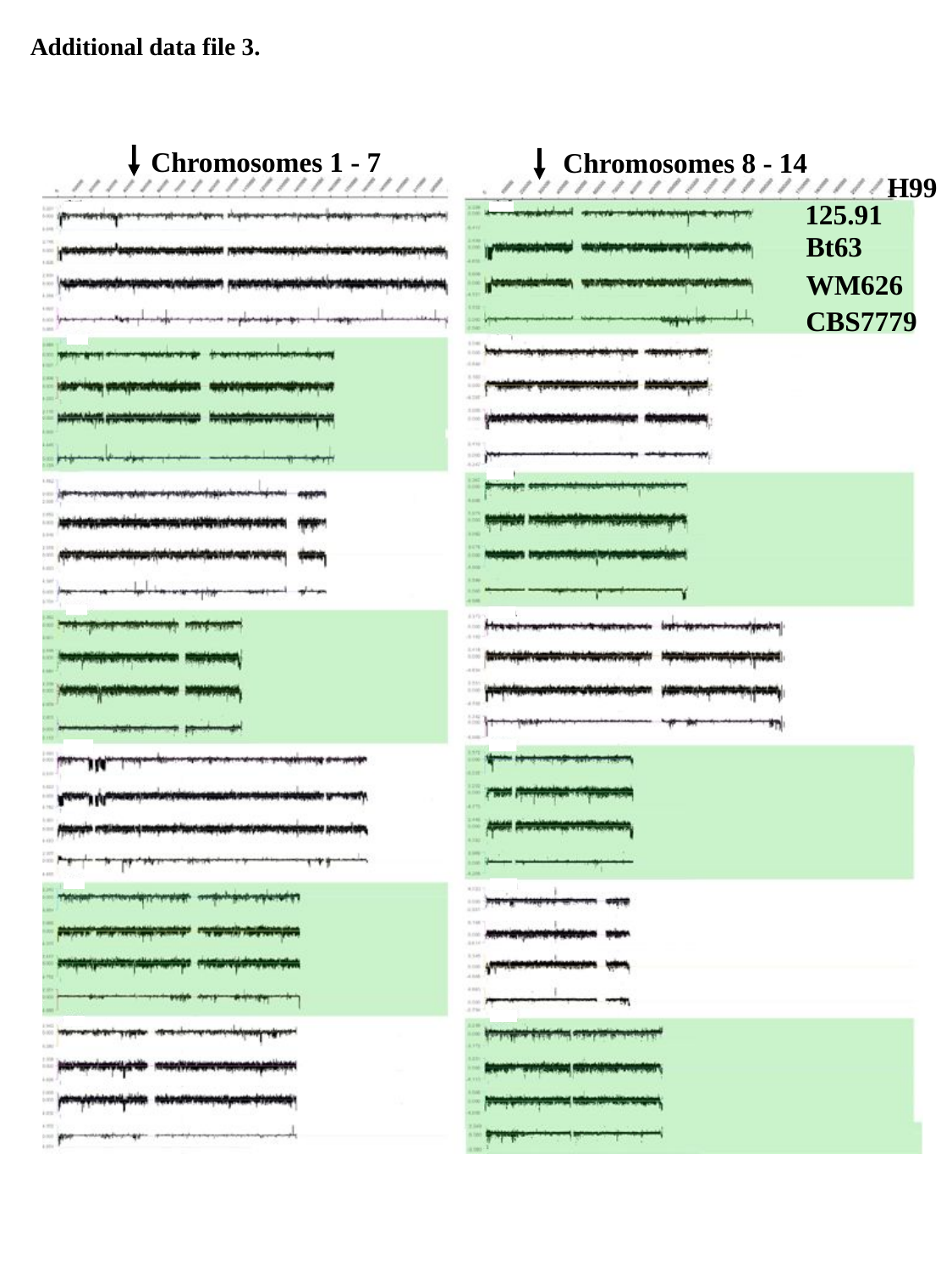

Additional data file 3.
Chromosomes 1 - 7
Chromosomes 8 - 14
H99
125.91
Bt63
WM626
CBS7779

Supplement: Additional data file 3 — Presented is a figure of the variation in the genomes of strains representing the three molecular subtypes within the A serotype of C. neoformans. [file gb-2008-9-2-r41-S3.ppt]

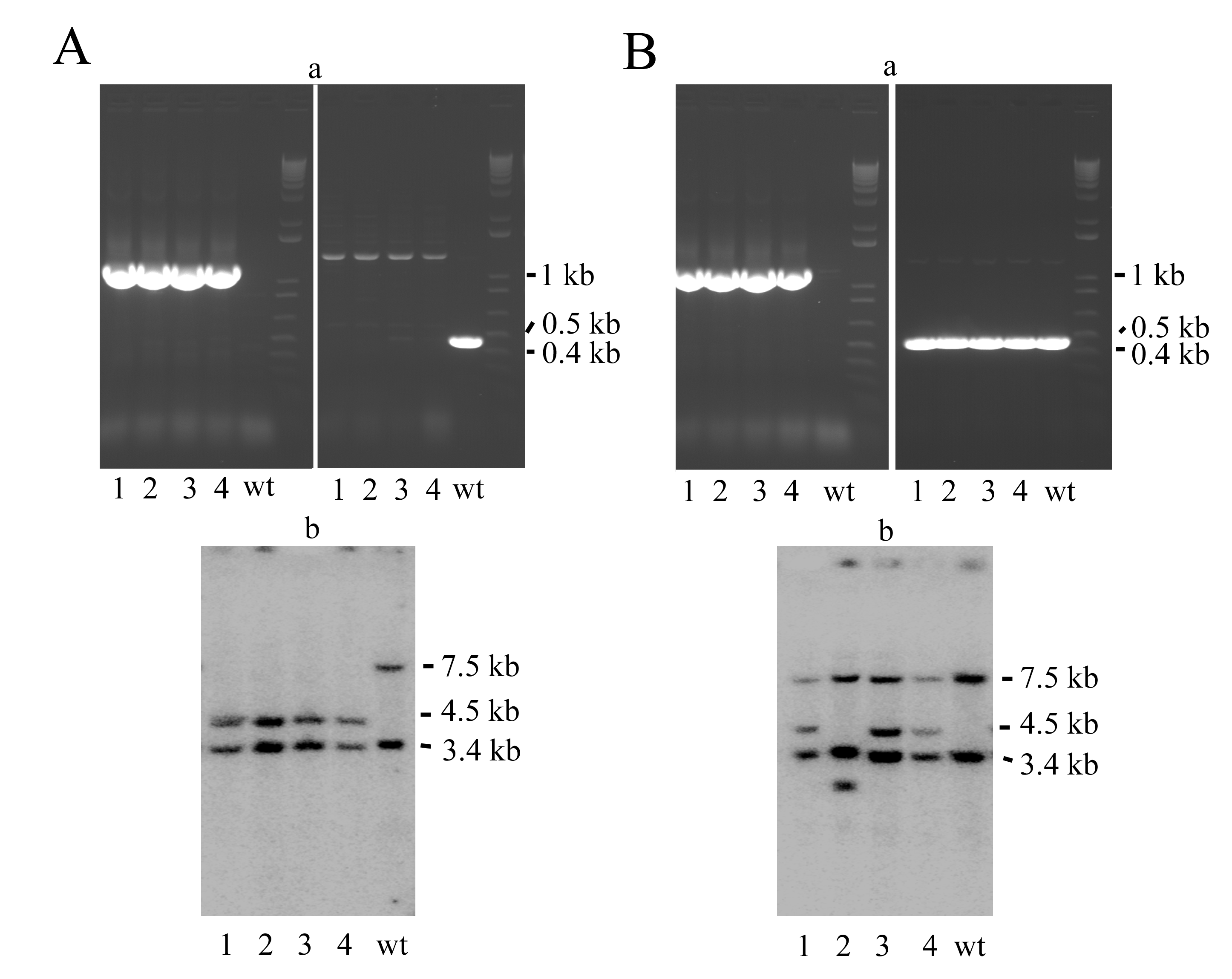

Supplement: Additional data file 7 — Presented is a figure showing the replacement of a gene on chromosome 13 to examine copy number. [file gb-2008-9-2-r41-S7.doc]

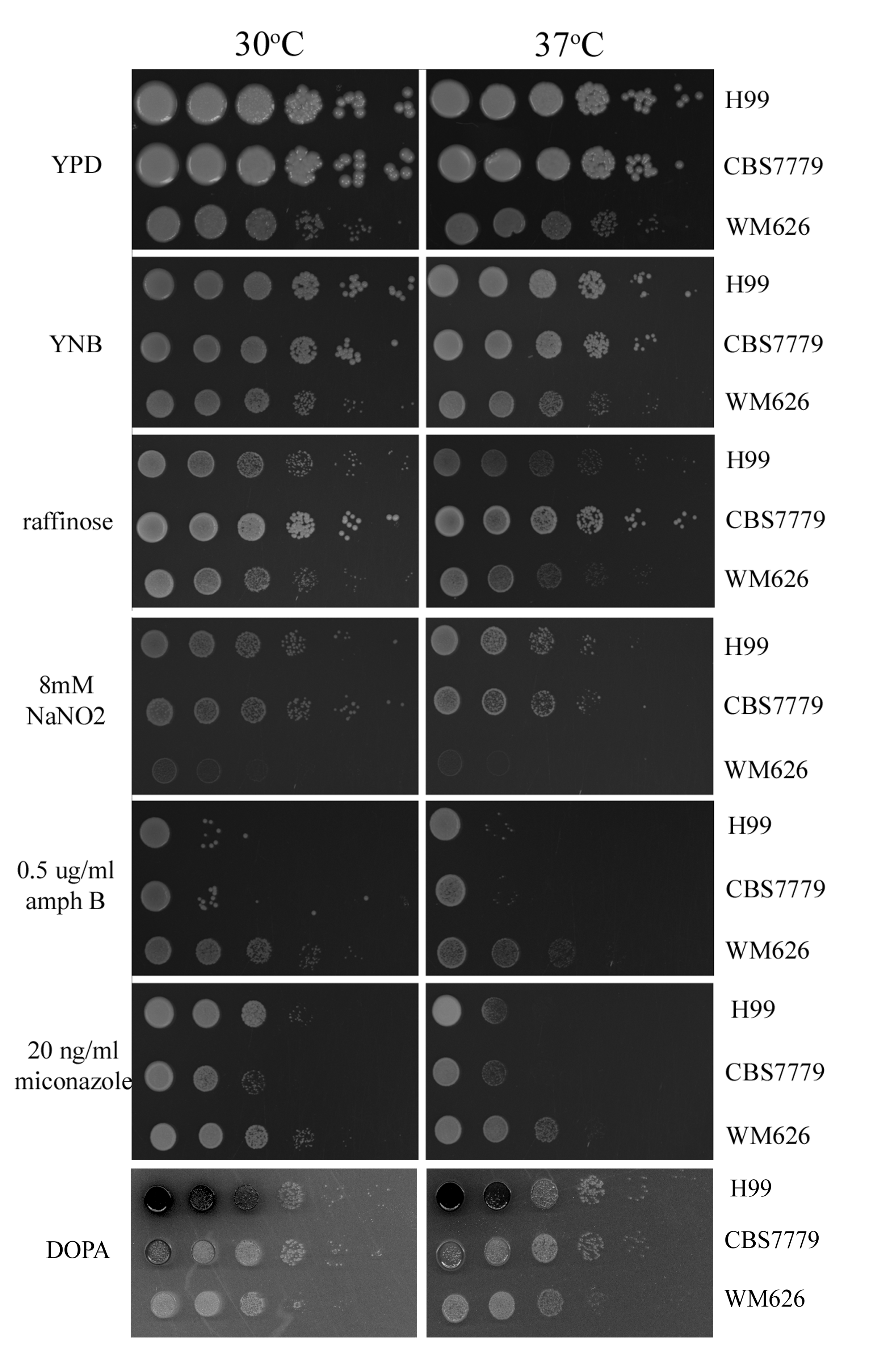

Supplement: Additional data file 9 — Presented is a figure showing the phenotypic differences between strains H99, CBS7779, and WM626. [file gb-2008-9-2-r41-S9.doc]

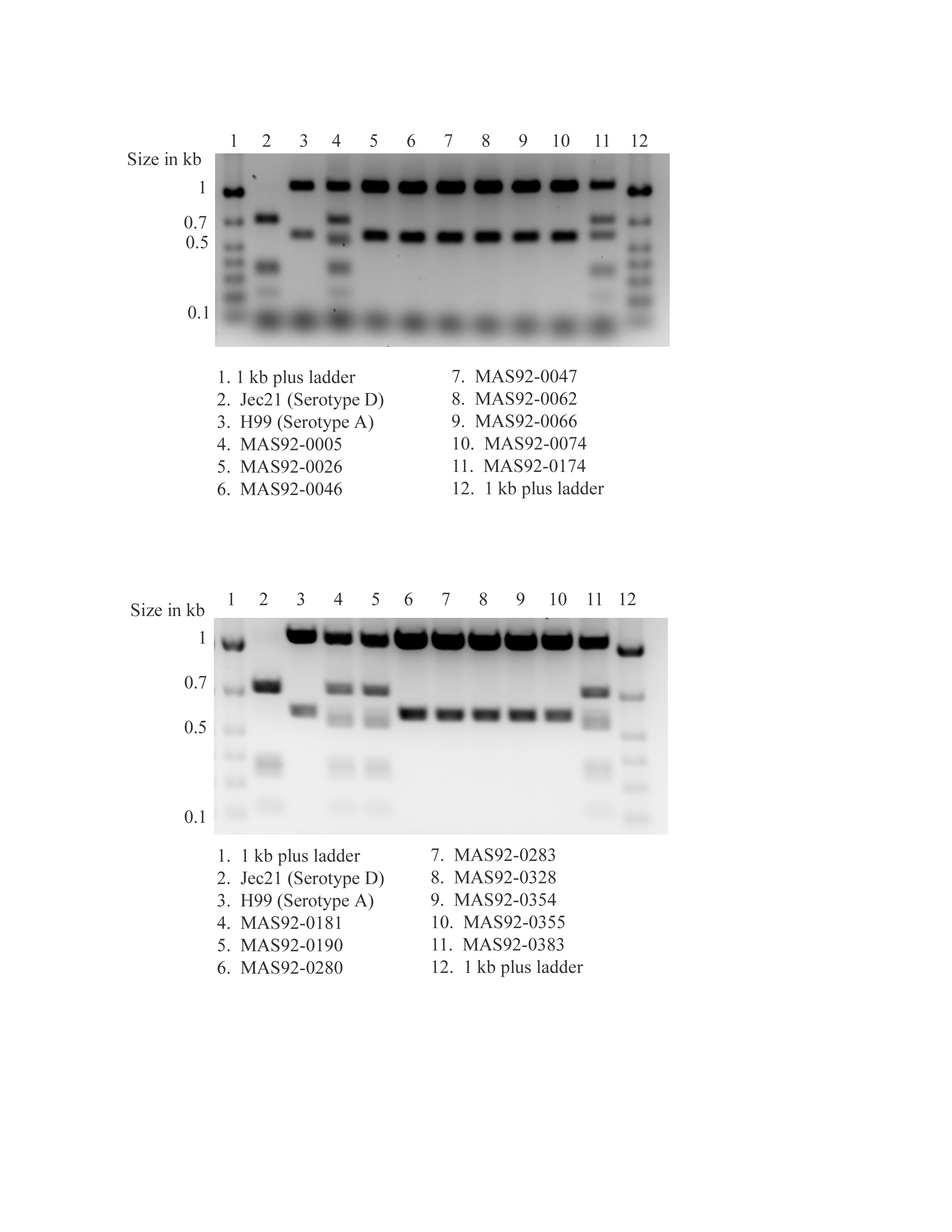

Supplement: Additional data file 11 — Presented is a figure showing an RFLP-PCR analysis of the origin of chromosome 1 in 16 AD hybrid strains. [file gb-2008-9-2-r41-S11.doc]
