# Supplementary material for: Comparative hybridization reveals extensive genome variation in the AIDS-associated pathogen Cryptococcus neoformans
Source: Genome Biol. 2008 Feb 22;9(2):R41. doi: 10.1186/gb-2008-9-2-r41 (PMC2374700; doi:10.1186/gb-2008-9-2-r41)
Supplement: Additional data file 4 — Presented is a table of regions of difference in the genomes of four serotype A strains compared with the sequenced genome of strain H99. [file gb-2008-9-2-r41-S4.doc]

| **Additional data file 4. Regions of difference in the genomes of four serotype A strains compared with the sequenced genome of strain H99.** | | | | | | | | | | | | | | | | | | |
| --- | --- | --- | --- | --- | --- | --- | --- | --- | --- | --- | --- | --- | --- | --- | --- | --- | --- | --- |
|  | | | | | | | **Average divergent Log2 ratio/strain** | | | | **Lowest Log2 ratio for region/strain** | | | | **Highest Log2 ratio for region/strain** | | | |
| **Chr** | **Coord.- segment^a^** | **Glean number^b^** | **GenBank ID^c^** | **E-value** | **Coord. -Gene^d^** | **Predicted function^e^** | Bt63 | **125.91** | **CBS**  **7779** | **WM**  **626** | **Bt63** | **125.91** | **CBS**  **7779** | **WM**  **626** | **Bt63** | **125.91** | **CBS**  **7779** | **WM**  **626** |
| 1 | 0 - 2000 | GLEAN_05258 | XP_001259191 | 7.00E-06 | 168 - 5406 | Helicase, putative  [Neosartorya fischeri]. | -2.392 | -0.887 | 1.073 | 1.176 | -4.326 | -1.689 | 0.752 | 0.298 | 1.389 | 0.534 | 1.633 | 2.445 |
|  | 12800 - 24800 | GLEAN_05259 | XP_747706 | 4.00E-04 | 12830 - 13578 | Amidohydrolase, putative  [Aspergillus fumigatus] | -3.11 | -2.89 |  |  | -4.826 | -5.545 |  |  | 0.555 | 0.903 |  |  |
|  | 12800 - 24800 | GLEAN_05255 | XP_567265 | 6.00E-58 | 14115 - 16370 | Drug transporter | -3.11 | -2.89 |  |  | -4.826 | -5.545 |  |  | 0.555 | 0.903 |  |  |
|  | 12800 - 24800 | GLEAN_05254 | BAE57378 | 0.023 | 16723 - 19388 | Unnamed protein product  [Aspergillus oryzae]. | -3.11 | -2.89 |  |  | -4.826 | -5.545 |  |  | 0.555 | 0.903 |  |  |
|  | 12800 - 24800 | GLEAN_05253 | XP_001261011 | 2.00E-177 | 20906 - 22642 | TPR domain protein  [Neosartorya fischeri] | -3.11 | -2.89 |  |  | -4.826 | -5.545 |  |  | 0.555 | 0.903 |  |  |
|  | 12800 - 24800 | GLEAN_05252 | XP_777999 | 2.00E-120 | 24790 - 26105 | Hypothetical protein | -3.11 | -2.89 |  |  | -4.826 | -5.545 |  |  | 0.555 | 0.903 |  |  |
|  | 339300 - 350700 | GLEAN_05190 | XP_568354 | 6.00E-65 | 341653 - 343375 | Hypothetical protein |  |  | 1.000 |  |  |  | -0.094 |  |  |  | 1.754 |  |
|  | 339300 - 350700 | GLEAN_05318 | EAU93688 | 1.00E-18 | 345835 - 349644 | Predicted protein [Coprinopsis cinerea]. |  |  | 1.000 |  |  |  | -0.094 |  |  |  | 1.754 |  |
|  | 339600 - 350000 | GLEAN_05190 | XP_568354 | 6.00E-65 | 341653 - 343375 | Hypothetical protein | 1.857 | -3.038 |  |  | -0.645 | -5.127 |  |  | 3.076 | -0.331 |  |  |
|  | 339600 - 350000 | GLEAN_05318 | EAU93688 | 1.00E-18 | 345835 - 349644 | Predicted protein [Coprinopsis cinerea]. | 1.857 | -3.038 |  |  | -0.645 | -5.127 |  |  | 3.076 | -0.331 |  |  |
|  | 344300 - 350200 | GLEAN_05318 | EAU93688 | 1.00E-18 | 345835 - 349644 | Predicted protein [Coprinopsis cinerea]. |  |  |  | 1.027 |  |  |  | -0.983 |  |  |  | 2.411 |
|  | 446300 - 450300 | GLEAN_05173 | XP_778147 | 3.00E-179 | 447026 - 448723 | Hypothetical protein |  |  |  | -2.261 |  |  |  | -4.192 |  |  |  | 0.82 |
|  | 999200 - 1002800 | GLEAN_05066 | XP_771810 | 9.00E-08 | 999727 - 1000137 | Hypothetical protein | -1.433 |  |  |  | -2.44 |  |  |  | -0.329 |  |  |  |
|  | 999200 - 1002800 | GLEAN_05065 | XP_567971 | 2.00E-83 | 1000224 - 1001851 | Retrotransposon nucleocapsid protein | -1.433 |  |  |  | -2.44 |  |  |  | -0.329 |  |  |  |
|  | 1001300 - 1002900 | GLEAN_05065 | XP_567971 | 2.00E-83 | 1000224 - 1001851 | Retrotransposon nucleocapsid protein |  |  |  | -1.677 |  |  |  | -3.516 |  |  |  | 0.414 |
|  | 1001300 - 1002900 | GLEAN_05460 | XP_771825 | 3.00E-85 | 1001982 - 1002620 | Hypothetical protein |  |  |  | -1.677 |  |  |  | -3.516 |  |  |  | 0.414 |
|  | 1375600 - 1376800 | GLEAN_04987 | XP_751881 | 6.00E-162 | 1354814 - 1358607 | Sodium P-type ATPase, putative [Aspergillus fumigatus]. | -2.194 |  |  |  | -4.328 |  |  |  | -0.621 |  |  |  |
|  | 1931000 - 1932300 | GLEAN_04878 | XP_567034 | 2.00E-55 | 1930978 - 1931772 | Hypothetical protein |  |  |  | -1.913 |  |  |  | -3.979 |  |  |  | -0.285 |
|  | 2150700 - 2159600 | GLEAN_04838 | XP_755943 | 3.00E-61 | 2146948 - 2150760 | Nuclear cohesin complex subunit (Psc3), putative [Aspergillus fumigatus]. |  |  |  | -1.683 |  |  |  | -3.197 |  |  |  | -0.003 |
|  | 2150700 - 2159600 | GLEAN_05671 | XP_566921 | 6.00E-60 | 2152581 - 2154878 | Hypothetical protein |  |  |  | -1.683 |  |  |  | -3.197 |  |  |  | -0.003 |
|  | 2150700 - 2159600 | GLEAN_04837 | XP_777765 | 0.00E+00 | 2155124 - 2159016 | Hypothetical protein |  |  |  | -1.683 |  |  |  | -3.197 |  |  |  | -0.003 |
|  | 2152600 - 2159000 | GLEAN_05617 | XP_566908 | 0.00E+00 | 2152581 - 2154878 | Hypothetical protein |  | -1.936 |  |  |  | -2.937 |  |  |  | -0.985 |  |  |
|  | 2152600 - 2159000 | GLEAN_04837 | XP_777765 | 0.00E+00 | 2155124 - 2159016 | Hypothetical protein |  | -1.936 |  |  |  | -2.937 |  |  |  | -0.985 |  |  |
|  | 2273900 - 2289800 | GLEAN_05695 | XP_776539 | 5.00E-27 | 2275865 - 2276751 | Hypothetical protein |  |  |  | -2.514 |  |  |  | -4.268 |  |  |  | -0.021 |
|  | 2273900 - 2289800 | GLEAN_05696 | XP_001421492 | 6.30E-01 | 2277032 - 2277691 | Predicted protein [Ostreococcus lucimarinus]. |  |  |  | -2.514 |  |  |  | -4.268 |  |  |  | -0.021 |
|  | 2273900 - 2289800 | GLEAN_05697 | XP_776387 | 1.00E-62 | 2279601 - 2282091 | Hypothetical protein |  |  |  | -2.514 |  |  |  | -4.268 |  |  |  | -0.021 |
|  | 2273900 - 2289800 | GLEAN_04816 | XP_568729 | 4.00E-84 | 2287656 - 2288399 | Retrotransposable element slacs 132 kda protein |  |  |  | -2.514 |  |  |  | -4.268 |  |  |  | -0.021 |
|  |  |  |  |  |  |  |  |  |  |  |  |  |  |  |  |  |  |  |
| 2 | 59000 - 60000 | GLEAN_02795 | XP_776539 | 1.00E-32 | 57196 - 59316 | Hypothetical protein |  |  |  | -0.644 |  |  |  | -1.861 |  |  |  | 0.084 |
|  | 88400 - 89700 | GLEAN_02825 | XP_568772 | 0.00E+00 | 84279 - 88794 | Gamma DNA-directed DNA polymerase |  |  |  | -1.269 |  |  |  | -2.638 |  |  |  | -0.151 |
|  | 88400 - 89700 | GLEAN_02791 | XP_568774 | 7.00E-25 | 89661 - 90954 | ATP synthase delta chain, mitochondrial precursor |  |  |  | -1.269 |  |  |  | -2.638 |  |  |  | -0.151 |
|  | 102000 - 102800 | GLEAN_02827 | XP_777007 | 9.00E-82 | 98930 - 103013 | Hypothetical protein |  | -1.931 |  |  |  | -4.031 |  |  |  | 0.659 |  |  |
|  | 150400 - 155600 | GLEAN_02839 | XP_776539 | 4.00E-31 | 150656 - 152503 | Hypothetical protein |  |  |  | -0.692 |  |  |  | -1.791 |  |  |  | 0.437 |
|  | 150400 - 155600 | GLEAN_02840 | XP_755620 | 8.00E-82 | 153876 - 155916 | Small nucleolar ribonucleoprotein complex subunit, putative [Aspergillus fumigatus]. |  |  |  | -0.692 |  |  |  | -1.791 |  |  |  | 0.437 |
|  | 219200 - 221000 | GLEAN_02851 | AAW41778 | 0.00E+00 | 219651 - 222324 | Chitin synthase regulator 3 |  |  |  | -2.273 |  |  |  | -3.834 |  |  |  | 0.068 |
|  | 260600 - 263900 | GLEAN_02861 | XP_772444 | 8.00E-73 | 260648 - 261960 | Hypothetical protein | -2.871 |  |  |  | -4.233 |  |  |  | -0.744 |  |  |  |
|  | 260600 - 263900 | GLEAN_02764 | EAU81015 | 3.40E-01 | 262148 - 263310 | Predicted protein [Coprinopsis cinerea]. | -2.871 |  |  |  | -4.233 |  |  |  | -0.744 |  |  |  |
|  | 267000 - 283800 | GLEAN_02863 | XP_776539 | 1.00E-31 | 268199 - 270025 | Hypothetical protein |  |  |  | -0.987 |  |  |  | -2.123 |  |  |  | -0.012 |
|  | 267000 - 283800 | GLEAN_02864 | XP_001275564 | 2.00E-38 | 271382 - 272566 | Small nucleolar ribonucleoprotein complex subunit, putative [Aspergillus clavatus]. |  |  |  | -0.987 |  |  |  | -2.123 |  |  |  | -0.012 |
|  | 267000 - 283800 | GLEAN_02865 | XP_001028745 | 4.00E-65 | 272970 - 275425 | Hypothetical protein [Tetrahymena thermophila]. |  |  |  | -0.987 |  |  |  | -2.123 |  |  |  | -0.012 |
|  | 267000 - 283800 | GLEAN_02761 | XP_001267666 | 3.00E-19 | 274227 - 274500 | Hypothetical protein [Neosartorya fischeri]. |  |  |  | -0.987 |  |  |  | -2.123 |  |  |  | -0.012 |
|  | 267000 - 283800 | GLEAN_02866 | NP_690845 | 1.00E-15 | 275643 - 275900 | Mitochondrial protein [Saccharomyces cerevisiae]. |  |  |  | -0.987 |  |  |  | -2.123 |  |  |  | -0.012 |
|  | 267000 - 283800 | GLEAN_02867 | EAT89175 | 5.00E-13 | 276346 - 276498 | Hypothetical protein [Phaeosphaeria nodorum]. |  |  |  | -0.987 |  |  |  | -2.123 |  |  |  | -0.012 |
|  | 267000 - 283800 | GLEAN_02868 | XP_001214108 | 7.00E-04 | 276583 - 276678 | Hypothetical protein [Aspergillus terreus]. |  |  |  | -0.987 |  |  |  | -2.123 |  |  |  | -0.012 |
|  | 267000 - 283800 | GLEAN_02869 | AAK13589 | 3.00E-15 | 277188 - 277878 | rRNA intron-encoded homing endonuclease [Oryza sativa]. |  |  |  | -0.987 |  |  |  | -2.123 |  |  |  | -0.012 |
|  | 267000 - 283800 | GLEAN_02870 | XP_778898 | 3.80E+00 | 278649 - 278891 | Hypothetical protein [Giardia lamblia]. |  |  |  | -0.987 |  |  |  | -2.123 |  |  |  | -0.012 |
|  | 267000 - 283800 | GLEAN_02759 | NP_013263 | 6.00E-01 | 280981 - 281144 | Putative protein [Saccharomyces cerevisiae]. |  |  |  | -0.987 |  |  |  | -2.123 |  |  |  | -0.012 |
|  | 267000 - 283800 | GLEAN_02871 | XP_665229 | 2.00E-38 | 281245 - 281900 | Senescence-associated protein [Cryptosporidium hominis]. |  |  |  | -0.987 |  |  |  | -2.123 |  |  |  | -0.012 |
|  | 267000 - 283800 | GLEAN_02758 | XP_001267666 | 3.00E-19 | 282502 - 282775 | Hypothetical protein [Neosartorya fischeri]. |  |  |  | -0.987 |  |  |  | -2.123 |  |  |  | -0.012 |
|  | 398700 - 402800 | GLEAN_02737 | XP_777205 | 2.00E-137 | 398261 - 399525 | Hypothetical protein | -0.888 |  |  |  | -3.066 |  |  |  | 0.634 |  |  |  |
|  | 398700 - 402800 | GLEAN_02893 | XP_568809 | 5.00E-65 | 400430 - 401114 | DNA-directed RNA polymerase | -0.888 |  |  |  | -3.066 |  |  |  | 0.634 |  |  |  |
|  | 419200 - 420400 | GLEAN_02897 | EAU92024 | 2.00E-09 | 419600 - 420387 | Predicted protein [Coprinopsis cinerea]. | -1.504 |  |  |  | -3.411 |  |  |  | 0.405 |  |  |  |
|  | 1406700 - 1408200 | GLEAN_03083 | XP_001274539 | 7.00E-04 | 1406896 - 1407932 | Stress response protein Rds1, putative [Aspergillus clavatus]. |  |  |  | -1.69 |  |  |  | -3.376 |  |  |  | -0.218 |
|  | 1408400 - 1409500 | GLEAN_03084 | XP_568991 | 6.00E-50 | 1409939 - 1410687 | Protein-methionine-R-oxide reductase | -2.521 |  |  |  | -3.619 |  |  |  | -1.262 |  |  |  |
|  | 1518400 - 1529300 | GLEAN_02513 | NP_268911 | 2.50E-02 | 1518456 - 1518773 | Hypothetical protein [Streptococcus phage 370.1]. |  |  |  | -1.796 |  |  |  | -4.5 |  |  |  | 1.489 |
|  | 1518400 - 1529300 | GLEAN_02512 | XP_566846 | 4.00E-89 | 1519702 - 1521218 | Transposable element- crypton-Cn1 |  |  |  | -1.796 |  |  |  | -4.5 |  |  |  | 1.489 |
|  | 1518400 - 1529300 | GLEAN_03106 | XP_777460 | 2.00E-75 | 1522304 - 1523255 | Hypothetical protein |  |  |  | -1.796 |  |  |  | -4.5 |  |  |  | 1.489 |
|  | 1518400 - 1529300 | GLEAN_03107 | XP_566922 | 2.00E-108 | 1524291 - 1525830 | Hypothetical protein |  |  |  | -1.796 |  |  |  | -4.5 |  |  |  | 1.489 |
|  | 1518400 - 1529300 | GLEAN_02511 | XP_566921 | 9.00E-05 | 1526263 - 1526669 | Hypothetical protein |  |  |  | -1.796 |  |  |  | -4.5 |  |  |  | 1.489 |
|  | 1518400 - 1529300 | GLEAN_02510 | XP_775648 | 3.00E-11 | 1527916 - 1528519 | Hypothetical protein |  |  |  | -1.796 |  |  |  | -4.5 |  |  |  | 1.489 |
|  | 1524800 - 1529200 | GLEAN_03107 | XP_566922 | 2.00E-108 | 1524291 - 1525830 | Hypothetical protein | -1.378 |  |  |  | -4.066 |  |  |  | 1.643 |  |  |  |
|  | 1524800 - 1529200 | GLEAN_02511 | XP_566921 | 9.00E-05 | 1526263 - 1526669 | Hypothetical protein | -1.378 |  |  |  | -4.066 |  |  |  | 1.643 |  |  |  |
|  | 1524800 - 1529200 | GLEAN_02510 | XP_775648 | 3.00E-11 | 1527916 - 1528519 | Hypothetical protein | -1.378 |  |  |  | -4.066 |  |  |  | 1.643 |  |  |  |
|  |  |  |  |  |  |  |  |  |  |  |  |  |  |  |  |  |  |  |
| 3 | 448700 - 450300 | GLEAN_04435 | YP_951772 | 8.00E-06 | 448806 - 449781 | Carboxymuconolactone decarboxylase [Mycobacterium vanbaalenii]. |  |  |  | -2.155 |  |  |  | -4.069 |  |  |  | -0.029 |
|  | 654000 - 655000 | GLEAN_04651 | XP_570011 | 4.00E-51 | 654633 - 655234 | UDP-glucose:sterol glucosyltransferase | -1.155 |  |  |  | -2.573 |  |  |  | 0.146 |  |  |  |
|  | 846400 - 847800 | GLEAN_04350 |  |  |  | Hypothetical protein |  |  | -1.136 |  |  |  | -3.704 |  |  |  | 0.156 |  |
|  | 859600 - 863400 | GLEAN_04348 | XP_776539 | 2.00E-34 | 860509 - 862619 | Hypothetical protein |  |  |  | -1.688 |  |  |  | -3.125 |  |  |  | -0.902 |
|  | 1230000 - 1230900 | GLEAN_04278 | XP_567785 | 3.00E-79 | 1229698 - 1230607 | Hypothetical protein |  | 1.251 |  |  |  | 0.592 |  |  |  | 1.886 |  |  |
|  | 1230000 - 1230900 | GLEAN_04757 | XP_758505 | 2.00E-25 | 1230749 - 1233656 | Hypothetical protein UM02358.1 [Ustilago maydis]. |  | 1.251 |  |  |  | 0.592 |  |  |  | 1.886 |  |  |
|  | 1334800 - 1340800 | GLEAN_04775 | XP_567626 | 2.00E-178 | 1334064 - 1335083 | Hypothetical protein | -0.775 |  |  |  | -2.276 |  |  |  | 1.294 |  |  |  |
|  | 1334800 - 1340800 | GLEAN_04776 | XP_567626 | 2.00E-20 | 1335789 - 1336219 | Hypothetical protein | -0.775 |  |  |  | -2.276 |  |  |  | 1.294 |  |  |  |
|  | 1334800 - 1340800 | GLEAN_04777 | AAM81269 | 2.00E-15 | 1337863 - 1339980 | NADH dehydrogenase subunit 4L [Cryptococcus neoformans var. grubii]. | -0.775 |  |  |  | -2.276 |  |  |  | 1.294 |  |  |  |
|  | 1336000 - 1340500 | GLEAN_04776 | XP_567626 | 2.00E-20 | 1335789 - 1336219 | Hypothetical protein |  |  |  | -1.837 |  |  |  | -4.503 |  |  |  | 1.054 |
|  | 1336000 - 1340500 | GLEAN_04777 | AAM81269 | 2.00E-15 | 1337863 - 1339980 | NADH dehydrogenase subunit 4L [Cryptococcus neoformans var. grubii]. |  |  |  | -1.837 |  |  |  | -4.503 |  |  |  | 1.054 |
|  | 1420400 - 1422800 | GLEAN_04794 | EAU92919 | 8.00E-58 | 1419521 - 1421658 | Hypothetical protein [Coprinopsis cinerea]. | 0.751 |  |  |  | 0.801 |  |  |  | 1.607 |  |  |  |
|  | 1420400 - 1422800 | GLEAN_04244 | EAU81955 | 4.00E-46 | 1422354 - 1426421 | Hypothetical protein [Coprinopsis cinerea]. | 0.751 |  |  |  | 0.801 |  |  |  | 1.607 |  |  |  |
|  | 1433300 - 1446600 | GLEAN_04242 | AAL35341 | 0.00E+00 | 1431670 - 1433335 | Sodium-hydrogen antiporter | -0.478 |  |  |  | -3.916 |  |  |  | 2.16 |  |  |  |
|  | 1433300 - 1446600 | GLEAN_04241 | XP_567607 | 1.00E-163 | 1434628 - 1437056 | Membrane transporter | -0.478 |  |  |  | -3.916 |  |  |  | 2.16 |  |  |  |
|  | 1433300 - 1446600 | GLEAN_04240 | NP_595254 | 2.00E-26 | 1439204 - 1440114 | Methyltransferase [Schizosaccharomyces pombe]. | -0.478 |  |  |  | -3.916 |  |  |  | 2.16 |  |  |  |
|  | 1433300 - 1446600 | GLEAN_04797 | XP_567605 | 5.00E-140 | 1440427 - 1442012 | Autophagy-related protein | -0.478 |  |  |  | -3.916 |  |  |  | 2.16 |  |  |  |
|  | 1433300 - 1446600 | GLEAN_04798 | XP_772605 | 5.00E-136 | 1442238 - 1443480 | Hypothetical protein | -0.478 |  |  |  | -3.916 |  |  |  | 2.16 |  |  |  |
|  | 1433300 - 1446600 | GLEAN_04239 | XP_567603 | 4.00E-08 | 1443932 - 1444264 | Hypothetical protein | -0.478 |  |  |  | -3.916 |  |  |  | 2.16 |  |  |  |
|  | 1433300 - 1446600 | GLEAN_04238 | XP_567603 | 7.00E-15 | 1444887 - 1445696 | Hypothetical protein | -0.478 |  |  |  | -3.916 |  |  |  | 2.16 |  |  |  |
|  | 1433300 - 1446600 | GLEAN_04237 | XP_772605 | 5.00E-136 | 1446737 - 1449052 | Hypothetical protein | -0.478 |  |  |  | -3.916 |  |  |  | 2.16 |  |  |  |
|  | 1433300 - 1446600 | GLEAN_04236 | XP_567601 | 5.00E-62 | 1450056 - 1451939 | Hypothetical protein | -0.478 |  |  |  | -3.916 |  |  |  | 2.16 |  |  |  |
|  | 1433300 - 1434900 | GLEAN_04242 | AAL35341 | 0.00E+00 | 1431670 - 1433335 | Sodium-hydrogen antiporter |  |  |  | -1.605 |  |  |  | -3.444 |  |  |  | 0.101 |
|  | 1433300 - 1434900 | GLEAN_04241 | XP_567607 | 1.00E-163 | 1434628 - 1437056 | Membrane transporter |  |  |  | -1.605 |  |  |  | -3.444 |  |  |  | 0.101 |
|  | 1565500 - 1571900 | GLEAN_04217 | XP_001258626 | 2.00E-115 | 1558846 - 1561544 | C6 finger domain protein, putative [Neosartorya fischeri]. |  |  |  | -3.193 |  |  |  | -4.823 |  |  |  | -0.445 |
|  | 1565500 - 1571900 | GLEAN_04216 | XP_748341 | 2.00E-176 | 1562223 - 1564413 | Hexose transporter protein [Aspergillus fumigatus]. |  |  |  | -3.193 |  |  |  | -4.823 |  |  |  | -0.445 |
|  | 1565500 - 1571900 | GLEAN_04215 | XP_001261612 | 2.00E-51 | 1566524 - 1567137 | Isochorismatase family hydrolase, putative [Neosartorya fischeri]. |  |  |  | -3.193 |  |  |  | -4.823 |  |  |  | -0.445 |
|  | 1565500 - 1571900 | GLEAN_04214 | AAG59831 | 3.00E-84 | 1568423 - 1571892 | Beta-glucosidase [Volvariella volvacea]. |  |  |  | -3.193 |  |  |  | -4.823 |  |  |  | -0.445 |
|  |  |  |  |  |  |  |  |  |  |  |  |  |  |  |  |  |  |  |
| 4 | 200 - 1100 | NO GENE |  |  |  |  |  |  | 1.123 |  |  |  | 0.882 |  |  |  | 1.697 |  |
|  | 142800 - 145200 | GLEAN_00688 | XP_572600 | 4.00E-130 | 142196 - 144358 | Cytosine-purine permease | -2.737 |  |  |  | -4.348 |  |  |  | 0.233 |  |  |  |
|  | 187200 - 188900 | GLEAN_00678 | XP_773452 | 1.00E-84 | 186294 - 188701 | Hypothetical protein |  | -2.323 |  |  |  | -3.601 |  |  |  |  |  |  |
|  | 233500 - 240200 | GLEAN_00670 | XP_568131 | 0.00E+00 | 235727 - 237978 | GabA permease |  |  |  | -2.297 |  |  |  | -4.659 |  |  |  | -0.037 |
|  | 233500 - 240200 | GLEAN_00669 | XP_001259379 | 4.00E-06 | 238465 - 239705 | Polyketide synthase, putative [Neosartorya fischeri]. |  |  |  | -2.297 |  |  |  | -4.659 |  |  |  | -0.037 |
|  | 233500 - 240200 | GLEAN_00759 | XP_773471 | 0.00E+00 | 240125 - 242641 | Hypothetical protein |  |  |  | -2.297 |  |  |  | -4.659 |  |  |  | -0.037 |
|  | 249200 - 255200 | GLEAN_00762 | XP_568124 | 0.00E+00 | 251303 - 253135 | Amine oxidase |  |  |  | -2.297 |  |  |  | -4.659 |  |  |  | -0.037 |
|  | 249200 - 255200 | GLEAN_00665 | XP_568123 | 9.00E-106 | 253643 - 254756 | Mango esterase |  |  |  | -2.297 |  |  |  | -4.659 |  |  |  | -0.037 |
|  | 382800 - 385200 | NO GENE | NO GENE |  |  |  | -1.899 |  |  |  | -3.502 |  |  |  | 0.728 | -0.046 |  |  |
|  | 436000 - 436900 | NO GENE | NO GENE |  |  |  | -2.842 |  |  |  | -4.537 |  |  |  | -0.216 |  |  |  |
|  | 646400 - 647900 | GLEAN_00841 | XP_567996 | 0.00E+00 | 647320 - 649005 | Hypothetical protein |  |  |  | -0.716 |  |  |  | -2.004 |  |  |  | 0.501 |
|  | 754600 - 756000 | GLEAN_00573 | XP_749615 | 6.00E-08 | 753095 - 754838 | BSD domain protein [Aspergillus fumigatus]. |  |  | -0.705 |  |  |  | -2.112 |  |  |  | -0.051 |  |
|  | 754600 - 756000 | GLEAN_00867 | XP_567968 | 0.00E+00 | 755150 - 757860 | Protein threonine/tyrosine kinase |  |  | -0.705 |  |  |  | -2.112 |  |  |  | -0.051 |  |
|  | 829000 - 831300 | GLEAN_00560 | XP_567737 | 1.00E-68 | 829807 - 830546 | Hypothetical protein |  |  |  | -1.132 |  |  |  | -3.538 |  |  |  | 0.548 |
|  | 884800 - 886200 | GLEAN_00550 | XP_572526 | 3.00E-73 | 884707 - 886512 | Membrane protein |  |  | 0.599 |  |  |  | 0.103 |  |  |  | 1.54 |  |
|  | 992300 - 994400 | GLEAN_00912 | XP_568378 | 3.00E-04 | 992540 - 993955 | Hypothetical protein |  |  |  | -2.776 |  |  |  | -3.889 |  |  |  | 0.485 |
|  | 1058000 - 1073700 | GLEAN_00516 | XP_001274983 | 3.00E-04 | 1058296 - 1059831 | Allantoate permease [Aspergillus clavatus]. | -2.449 |  |  |  | -4.684 |  |  |  | 0.285 |  |  |  |
|  | 1058000 - 1073700 | GLEAN_00923 | XP_567775 | 9.00E-10 | 1061847 - 1063580 | Beta-fructofuranosidase | -2.449 |  |  |  | -4.684 |  |  |  | 0.285 |  |  |  |
|  | 1058000 - 1073700 | GLEAN_00515 | XP_001222740 | 4.20E+00 | 1063965 - 1065856 | Hypothetical protein [Chaetomium globosum]. | -2.449 |  |  |  | -4.684 |  |  |  | 0.285 |  |  |  |
|  | 1058000 - 1073700 | GLEAN_00924 | XP_772566 | 6.00E-37 | 1067407 - 1070812 | Hypothetical protein | -2.449 |  |  |  | -4.684 |  |  |  | 0.285 |  |  |  |
|  | 1058000 - 1073700 | GLEAN_00514 | XP_001259059 | 5.00E-61 | 1071378 - 1072715 | NAD-binding Rossmann fold oxidoreductase family protein [Neosartorya fischeri]. | -2.449 |  |  |  | -4.684 |  |  |  | 0.285 |  |  |  |
|  | 1058000 - 1073700 | GLEAN_00925 | XP_571460 | 0.00E+00 | 1073621 - 1075767 | Trehalose transporter | -2.449 |  |  |  | -4.684 |  |  |  | 0.285 |  |  |  |
|  | 1066700 - 1080400 | GLEAN_00924 | XP_772566 | 6.00E-37 | 1067407 - 1070812 | Hypothetical protein |  |  |  | -2.514 |  |  |  | -4.426 |  |  |  | -0.075 |
|  | 1066700 - 1080400 | GLEAN_00514 | XP_001259059 | 5.00E-61 | 1071378 - 1072715 | NAD-binding Rossmann fold oxidoreductase family protein [Neosartorya fischeri]. |  |  |  | -2.514 |  |  |  | -4.426 |  |  |  | -0.075 |
|  | 1066700 - 1080400 | GLEAN_00925 | XP_571460 | 0.00E+00 | 1073621 - 1075767 | Trehalose transporter |  |  |  | -2.514 |  |  |  | -4.426 |  |  |  | -0.075 |
|  | 1066700 - 1080400 | GLEAN_00926 | XP_775062 | 1.00E-51 | 1078797 - 1079543 | Hypothetical protein |  |  |  | -2.514 |  |  |  | -4.426 |  |  |  | -0.075 |
|  |  |  |  |  |  |  |  |  |  |  |  |  |  |  |  |  |  |  |
| 5 | 0 - 5200 | GLEAN_02155 | NO GENE |  |  |  |  |  |  | -2.179 |  |  |  | -3.865 |  |  |  | 0.254 |
|  | 50 - 800 | GLEAN_02155 | NO GENE |  |  |  |  |  | -0.75 |  |  |  | -1.479 |  |  |  | -0.104 |  |
|  | 4200 - 22000 | GLEAN_02155 | NO GENE |  |  |  |  |  | 1.104 |  |  |  | 0.014 |  |  |  | 1.594 |  |
|  | 4200 - 22000 | GLEAN_02156 | XP_572149 | 4.00E-40 | 6685 - 6955 | Hypothetical protein |  |  | 1.104 |  |  |  | 0.014 |  |  |  | 1.594 |  |
|  | 4200 - 22000 | GLEAN_02154 | XP_775060 | 1.00E-165 | 7285 - 8614 | Hypothetical protein |  |  | 1.104 |  |  |  | 0.014 |  |  |  | 1.594 |  |
|  | 4200 - 22000 | GLEAN_02157 | XP_753692 | 3.00E-73 | 9145 - 10906 | MFS allantoate transporter, putative [Aspergillus fumigatus]. |  |  | 1.104 |  |  |  | 0.014 |  |  |  | 1.594 |  |
|  | 4200 - 22000 | GLEAN_02153 | XP_001264611 | 9.00E-29 | 11829 - 12746 | HpcH/HpaI aldolase/citrate lyase family protein [Neosartorya fischeri]. |  |  | 1.104 |  |  |  | 0.014 |  |  |  | 1.594 |  |
|  | 4200 - 22000 | GLEAN_02152 | XP_751307 | 3.00E-163 | 14554 - 16504 | 5-oxo-L-prolinase, putative [Aspergillus fumigatus]. |  |  | 1.104 |  |  |  | 0.014 |  |  |  | 1.594 |  |
|  | 4200 - 22000 | GLEAN_02158 | CAD70763 | 0.00E+00 | 16982 - 19485 | 5-oxoprolinase |  |  | 1.104 |  |  |  | 0.014 |  |  |  | 1.594 |  |
|  | 4200 - 22000 | GLEAN_02159 | EAU83894 | 7.00E-29 | 21439 - 24277 | Hypothetical protein [Coprinopsis cinerea]. |  |  | 1.104 |  |  |  | 0.014 |  |  |  | 1.594 |  |
|  | 6700 - 32800 | GLEAN_02156 | XP_572149 | 4.00E-40 | 6685 - 6955 | Hypothetical protein | -3.035 |  |  |  | -4.782 |  |  |  | 1.11 |  |  |  |
|  | 6700 - 32800 | GLEAN_02154 | XP_775060 | 1.00E-165 | 7285 - 8614 | Hypothetical protein | -3.035 |  |  |  | -4.782 |  |  |  | 1.11 |  |  |  |
|  | 6700 - 32800 | GLEAN_02157 | XP_753692 | 3.00E-73 | 9145 - 10906 | MFS allantoate transporter, putative [Aspergillus fumigatus]. | -3.035 |  |  |  | -4.782 |  |  |  | 1.11 |  |  |  |
|  | 6700 - 32800 | GLEAN_02153 | XP_001264611 | 9.00E-29 | 11829 - 12746 | HpcH/HpaI aldolase/citrate lyase family protein [Neosartorya fischeri]. | -3.035 |  |  |  | -4.782 |  |  |  | 1.11 |  |  |  |
|  | 6700 - 32800 | GLEAN_02152 | XP_751307 | 3.00E-163 | 14554 - 16504 | 5-oxo-L-prolinase, putative [Aspergillus fumigatus]. | -3.035 |  |  |  | -4.782 |  |  |  | 1.11 |  |  |  |
|  | 6700 - 32800 | GLEAN_02158 | CAD70763 | 0.00E+00 | 16982 - 19485 | 5-oxoprolinase | -3.035 |  |  |  | -4.782 |  |  |  | 1.11 |  |  |  |
|  | 6700 - 32800 | GLEAN_02159 | EAU83894 | 7.00E-29 | 21439 - 24277 | Hypothetical protein [Coprinopsis cinerea]. | -3.035 |  |  |  | -4.782 |  |  |  | 1.11 |  |  |  |
|  | 6700 - 32800 | GLEAN_02150 | XP_748173 | 5.00E-23 | 24811 - 25964 | DUF1445 domain protein [Aspergillus fumigatus]. | -3.035 |  |  |  | -4.782 |  |  |  | 1.11 |  |  |  |
|  | 6700 - 32800 | GLEAN_02160 | XP_570691 | 0.00E+00 | 27696 - 29485 | Hypothetical protein | -3.035 |  |  |  | -4.782 |  |  |  | 1.11 |  |  |  |
|  | 6700 - 32800 | GLEAN_02149 | XP_570692 | 5.00E-130 | 30264 - 32039 | Enolase 1 | -3.035 |  |  |  | -4.782 |  |  |  | 1.11 |  |  |  |
|  | 17700 - 20800 | GLEAN_02158 | XP_571053 | 0.00E+00 | 16982 - 19485 | 5-oxoprolinase |  |  |  | 0.696 |  |  |  | -1.158 |  |  |  | 1.725 |
|  | 27400 - 29500 | GLEAN_02160 | XP_570691 | 0.00E+00 | 27696 - 29485 | Hypothetical protein |  |  |  | -2.452 |  |  |  | -4.32 |  |  |  | 0.296 |
|  | 94100 - 96600 | GLEAN_02177 | XP_776197 | 1.00E-131 | 95790 - 98444 | Hypothetical protein |  |  |  | -3.135 |  |  |  | -4.423 |  |  |  | -0.445 |
|  | 126800 - 128700 | NO GENE | NO GENE |  |  |  |  |  |  | -2.124 |  |  |  | -3.576 |  |  |  | -0.04 |
|  | 126800 - 128700 | GLEAN_02183 | XP_570389 | 1.00E-144 | 128556 - 131058 | Glucosidase |  |  |  | -2.124 |  |  |  | -3.576 |  |  |  | -0.04 |
|  | 184800 - 204000 | GLEAN_02192 | AAN75167 | 7.00E-170 | 183859 - 185218 | FAO1 [Cryptococcus neoformans var. grubii]. | -2.324 | -2.371 |  |  | -4.656 | -4.865 |  |  | 1.015 |  |  |  |
|  | 184800 - 204000 | GLEAN_02193 | XP_570106 | 6.00E-157 | 185988 - 187349 | Sexual development regulator | -2.324 | -2.371 |  |  | -4.656 | -4.865 |  |  | 1.015 | 0.693 |  |  |
|  | 184800 - 204000 | GLEAN_02194 | XP_773197 | 9.00E-69 | 187761 - 188578 | Hypothetical protein | -2.324 | -2.371 |  |  | -4.656 | -4.865 |  |  | 1.015 | 0.693 |  |  |
|  | 184800 - 204000 | GLEAN_02126 | AAY25038 | 6.00E-71 | 189844 - 192096 | CAP1 [Cryptococcus gattii]. | -2.324 | -2.371 |  |  | -4.656 | -4.865 |  |  | 1.015 | 0.693 |  |  |
|  | 184800 - 204000 | GLEAN_02124 | XP_755038 | 1.00E-152 | 194439 - 200633 | Phospholipase D1 (PLD1), putative [Aspergillus fumigatus]. | -2.324 | -2.371 |  |  | -4.656 | -4.865 |  |  | 1.015 | 0.693 |  |  |
|  | 222400 - 238400 | GLEAN_02119 | AAS92522 | 8.00E-04 | 222500 - 222616 | MF alpha; hypothetical protein [Cryptococcus gattii]. | -3.317 | -3.069 |  |  | -4.766 | -5.517 |  |  | 0.282 | 0.693 |  |  |
|  | 222400 - 238400 | GLEAN_02119 | AAK55608 | 8.00E-04 | 222500 - 222616 | Pheromone alpha | -3.317 | -3.069 |  |  | -4.766 | -5.517 |  |  | 0.282 | -0.06 |  |  |
|  | 222400 - 238400 | GLEAN_02200 | AAS92522 | 2.00E-03 | 222931 - 223047 | MF alpha; hypothetical protein [Cryptococcus gattii]. | -3.317 | -3.069 |  |  | -4.766 | -5.517 |  |  | 0.282 | -0.06 |  |  |
|  | 222400 - 238400 | GLEAN_02201 | XP_001273891 | 1.00E-109 | 224303 - 230136 | Class V myosin (Myo4), putative [Aspergillus clavatus]. | -3.317 | -3.069 |  |  | -4.766 | -5.517 |  |  | 0.282 | -0.06 |  |  |
|  | 222400 - 238400 | GLEAN_02118 | AAN75615 | 6.00E-134 | 230585 - 232996 | STE20 | -3.317 | -3.069 |  |  | -4.766 | -5.517 |  |  | 0.282 | -0.06 |  |  |
|  | 247300 - 288200 | GLEAN_02116 | XP_001265776 | 2.00E-143 | 245788 - 247860 | Dihydrolipoamide dehydrogenase [Neosartorya fischeri]. | -2.167 | -1.88 |  |  | -4.607 | -4.973 |  |  | 1.011 | 1.447 |  |  |
|  | 247300 - 288200 | GLEAN_02115 | EAU87643 | 9.00E-51 | 248166 - 251116 | Hypothetical protein [Coprinopsis cinerea]. | -2.167 | -1.88 |  |  | -4.607 | -4.973 |  |  | 1.011 | 1.447 |  |  |
|  | 247300 - 288200 | GLEAN_02114 | XP_570546 | 7.00E-155 | 252101 - 255269 | Hypothetical protein | -2.167 | -1.88 |  |  | -4.607 | -4.973 |  |  | 1.011 | 1.447 |  |  |
|  | 247300 - 288200 | GLEAN_02204 | XP_748000 | 6.00E-99 | 256901 - 262890 | PHD transcription factor (Rum1), putative [Aspergillus fumigatus]. | -2.167 | -1.88 |  |  | -4.607 | -4.973 |  |  | 1.011 | 1.447 |  |  |
|  | 247300 - 288200 | GLEAN_02205 | AAV98454 | 6.00E-164 | 263567 - 266323 | BSP1 [Cryptococcus neoformans var. grubii]. | -2.167 | -1.88 |  |  | -4.607 | -4.973 |  |  | 1.011 | 1.447 |  |  |
|  | 380400 - 382100 | GLEAN_02227 | XP_776063 | 5.00E-88 | 380101 - 380994 | Hypothetical protein |  | -1.965 |  |  |  | -4.368 |  |  |  | 1.447 |  |  |
|  | 380400 - 382100 | GLEAN_02228 | XP_570055 | 2.00E-63 | 381223 - 382308 | Hypothetical protein |  | -1.965 |  |  |  | -4.368 |  |  |  | 0.092 |  |  |
|  | 380300 - 382300 | GLEAN_02227 | XP_776063 | 5.00E-88 | 380101 - 380994 | Hypothetical protein |  |  | -1.669 |  |  |  | -4.465 |  |  |  | 0.027 |  |
|  | 380300 - 382300 | GLEAN_02228 | XP_570055 | 2.00E-63 | 381223 - 382308 | Hypothetical protein |  |  | -1.669 |  |  |  | -4.465 |  |  |  | 0.027 |  |
|  | 478600 - 488000 | GLEAN_02072 | XP_771818 | 5.00E-67 | 476013 - 478809 | Hypothetical protein |  |  |  | -1.349 |  |  |  | -3.573 |  |  |  | 1.139 |
|  | 478600 - 488000 | GLEAN_02071 | XP_569289 | 4.00E-51 | 479169 - 479992 | Hypothetical protein |  |  |  | -1.349 |  |  |  | -3.573 |  |  |  | 1.139 |
|  | 478600 - 488000 | GLEAN_02252 | XP_566921 | 6.00E-60 | 481074 - 483301 | Hypothetical protein |  |  |  | -1.349 |  |  |  | -3.573 |  |  |  | 1.139 |
|  | 478600 - 488000 | GLEAN_02070 | XP_777765 | 0.00E+00 | 483547 - 487439 | Hypothetical protein |  |  |  | -1.349 |  |  |  | -3.573 |  |  |  | 1.139 |
|  | 478700 - 488500 | GLEAN_02072 | XP_771818 | 5.00E-67 | 476013 - 478809 | Hypothetical protein |  |  | 0.435 |  |  |  | 0.023 |  |  |  | 1.072 |  |
|  | 478700 - 488500 | GLEAN_02071 | XP_569289 | 4.00E-51 | 479169 - 479992 | Hypothetical protein |  |  | 0.435 |  |  |  | 0.023 |  |  |  | 1.072 |  |
|  | 478700 - 488500 | GLEAN_02252 | XP_566921 | 6.00E-60 | 481074 - 483301 | Hypothetical protein |  |  | 0.435 |  |  |  | 0.023 |  |  |  | 1.072 |  |
|  | 478700 - 488500 | GLEAN_02070 | XP_777765 | 0.00E+00 | 483547 - 487439 | Hypothetical protein |  |  | 0.435 |  |  |  | 0.023 |  |  |  | 1.072 |  |
|  | 480500 - 487300 | GLEAN_02252 | XP_566921 | 6.00E-60 | 481074 - 483301 | Hypothetical protein | -2.058 | -2.045 |  |  | -3.563 | -3.707 |  |  | -0.409 | 0.092 |  |  |
|  | 480500 - 487300 | GLEAN_02070 | XP_777765 | 0.00E+00 | 483547 - 487439 | Hypothetical protein | -2.058 | -2.045 |  |  | -3.563 | -3.707 |  |  | -0.409 | 0.864 |  |  |
|  | 589800 - 591500 | GLEAN_02271 | AAZ28943 | 3.00E-24 | 587866 - 588468 | Polyprotein [Phanerochaete chrysosporium]. | 1.591 |  |  |  | 0.441 |  |  |  | 3.284 |  |  |  |
|  | 589800 - 591500 | GLEAN_02272 | XP_776289 | 1.00E-15 | 588520 - 589247 | Hypothetical protein | 1.591 |  |  |  | 0.441 |  |  |  | 3.284 |  |  |  |
|  | 1776000 - 1786400 | GLEAN_01807 | XP_001258991 | 4.00E-153 | 1775551 - 1780923 | ATP-binding cassette transporter [Neosartorya fischeri]. | 1.025 |  |  |  | -0.553 |  |  |  | 1.952 |  |  |  |
|  | 1776000 - 1786400 | GLEAN_01806 | XP_570359 | 6.00E-165 | 1782265 - 1784468 | Myo-inositol transporter | 1.025 |  |  |  | -0.553 |  |  |  | 1.952 |  |  |  |
|  | 1790000 - 1792900 | GLEAN_01804 | DAA05956 | 8.00E-98 | 1790719 - 1791427 | putative O-acetyl transferase [Cryptococcus neoformans var. grubii]. | -2.585 |  |  |  | -4.05 |  |  |  | 0.274 |  |  |  |
|  | 1809200 - 1814500 | GLEAN_02489 | 1E3W_A | 2.00E-06 | 1808147 - 1809297 | Chain A, Rat Brain 3-Hydroxyacyl-CoA Dehydrogenase |  |  |  | -1.541 |  |  |  | -2.701 |  |  |  | 0.12 |
|  | 1809200 - 1814500 | GLEAN_02490 | XP_776539 | 2.00E-31 | 1811119 - 1813224 | Hypothetical protein |  |  |  | -1.541 |  |  |  | -2.701 |  |  |  | 0.12 |
|  |  |  |  |  |  |  |  |  |  |  |  |  |  |  |  |  |  |  |
| 6 | 2100 - 3800 | GLEAN_05969 | XP_572787 | 2.00E-156 | 3409 - 5246 | Fungal specific transcription factor | -2.189 |  |  |  | -4.017 |  |  |  | 0.356 |  |  |  |
|  | 499200 - 501200 | NO GENE | NO GENE |  |  |  | -1.534 |  |  |  | -3.854 |  |  |  | 0.779 |  |  |  |
|  | 730000 - 733000 | GLEAN_05833 | AAZ28942 | 1.00E-08 | 730825 - 731331 | Polyprotein [Phanerochaete chrysosporium]. | -0.855 |  |  |  | -2.568 |  |  |  | 1.497 |  |  |  |
|  | 988000 - 991200 | GLEAN_05782 | XP_570931 | 4.00E-06 | 987694 - 990861 | Exonuclease II |  | 0.566 |  |  |  | 0.074 |  |  |  | 1.476 |  |  |
|  | 1049100 - 1051600 | GLEAN_05773 | NP_508554 | 3.10E+00 | 1049803 - 1050555 | Prion-like-(Q/N-rich)-domain-bearing protein family member (pqn-37) [Caenorhabditis elegans]. | -0.979 |  |  |  | -2.474 |  |  |  | 0.867 | 1.476 |  |  |
|  | 1060400 - 1062800 | GLEAN_05771 | XP_753874 | 1.30E+00 | 1061163 - 1062348 | Inositol 5-phosphatase, putative [Aspergillus fumigatus]. | -0.979 |  |  |  | -2.474 |  |  |  | 0.867 |  |  |  |
|  | 1174800 - 1179200 | GLEAN_06199 | XP_001274925 | 3.00E-07 | 1174200 - 1174846 | MGMT family protein [Aspergillus clavatus]. | -0.782 |  |  |  | -2.599 |  |  |  | 1.233 |  |  |  |
|  | 1174800 - 1179200 | GLEAN_05747 | XP_571089 | 4.00E-149 | 1175332 - 1176294 | Phosphatidylinositol transporter | -0.782 |  |  |  | -2.599 |  |  |  | 1.233 |  |  |  |
|  | 1174800 - 1179200 | GLEAN_05746 | CAE76225 | 9.00E-05 | 1177308 - 1183318 | Related to putative cytoplasmic structural protein [Neurospora crassa]. | -0.782 |  |  |  | -2.599 |  |  |  | 1.233 |  |  |  |
|  | 1313000 - 1315100 | GLEAN_06217 | XP_524999 | 3.90E+00 | 1314349 - 1314725 | prostaglandin-endoperoxide synthase 2 [Pan troglodytes]. | -2.301 |  |  |  | -4.317 |  |  |  | 0.109 |  |  |  |
|  | 1322000 - 1326100 | GLEAN_06220 | XP_775207 | 9.00E-145 | 1320689 - 1322100 | Hypothetical protein | -1.049 |  |  |  | -2.681 |  |  |  | 1.215 |  |  |  |
|  | 1322000 - 1326100 | GLEAN_06221 | XP_775208 | 5.00E-124 | 1322542 - 1324133 | Hypothetical protein | -1.049 |  |  |  | -2.681 |  |  |  | 1.215 |  |  |  |
|  | 1322000 - 1326100 | GLEAN_05716 | XP_001269413 | 4.00E-38 | 1324193 - 1325679 | Dimeric dihydrodiol dehydrogenase, putative [Aspergillus clavatus]. | -1.049 |  |  |  | -2.681 |  |  |  | 1.215 |  |  |  |
|  | 109300 - 112200 | GLEAN_05950 | XP_568595 | 3.00E-08 | 110446 - 111423 | Hypothetical protein |  |  |  | -1.965 |  |  |  | -4.119 |  |  |  | 0.376 |
|  | 201200 - 204000 | GLEAN_06010 | XP_570806 | 2.00E-126 | 199934 - 201582 | Vacuole protein |  |  |  | -0.996 |  |  |  | -2.878 |  |  |  | 0.3 |
|  | 201200 - 204000 | GLEAN_06011 | XP_775357 | 0.00E+00 | 203319 - 205522 | Hypothetical protein |  |  |  | -0.996 |  |  |  | -2.878 |  |  |  | 0.3 |
|  | 212900 - 224600 | GLEAN_06014 | EDK25849 | 2.00E-07 | 211750 - 213126 | 3-carboxymuconate cyclase [Vibrionales bacterium]. |  |  |  | -0.891 |  |  |  | -3.347 |  |  |  | 1.567 |
|  | 212900 - 224600 | GLEAN_06015 | XP_775360 | 0.00E+00 | 213646 - 217692 | Hypothetical protein |  |  |  | -0.891 |  |  |  | -3.347 |  |  |  | 1.567 |
|  | 212900 - 224600 | GLEAN_05932 | XP_570814 | 0.00E+00 | 218037 - 220112 | Flavin-containing monooxygenase |  |  |  | -0.891 |  |  |  | -3.347 |  |  |  | 1.567 |
|  | 212900 - 224600 | GLEAN_06016 | XP_748637 | 7.00E-80 | 222190 - 223649 | NAD binding Rossmann fold oxidoreductase, putative [Aspergillus fumigatus]. |  |  |  | -0.891 |  |  |  | -3.347 |  |  |  | 1.567 |
|  | 446700 - 453700 | GLEAN_05896 | XP_001264569 | 1.00E-24 | 442072 - 446783 | SH3 domain protein, putative [Neosartorya fischeri]. |  |  |  | -2.345 |  |  |  | -4.752 |  |  |  | 0.16 |
|  | 446700 - 453700 | GLEAN_06061 | XP_569289 | 2.00E-09 | 447800 - 448867 | Hypothetical protein |  |  |  | -2.345 |  |  |  | -4.752 |  |  |  | 0.16 |
|  | 446700 - 453700 | GLEAN_05895 | XP_566921 | 9.00E-16 | 450454 - 451207 | Hypothetical protein |  |  |  | -2.345 |  |  |  | -4.752 |  |  |  | 0.16 |
|  | 446700 - 453700 | GLEAN_05894 | ABA98804 | 4.00E-36 | 452045 - 452669 | Retrotransposon protein, putative, Ty1-copia subclass [Oryza sativa] |  |  |  | -2.345 |  |  |  | -4.752 |  |  |  | 0.16 |
|  | 727800 - 733800 | GLEAN_05833 | AAZ28942 | 1.00E-08 | 730825 - 731331 | Polyprotein [Phanerochaete chrysosporium]. |  |  |  | -2.45 |  |  |  | -4.257 |  |  |  | 1.308 |
|  | 727800 - 733800 | GLEAN_05832 | NP_350221 | 3.70E+00 | 731542 - 732501 | Oligopeptide ABC transporter, permease component [Clostridium acetobutylicum]. |  |  |  | -2.45 |  |  |  | -4.257 |  |  |  | 1.308 |
|  | 727800 - 733800 | GLEAN_06118 | XP_001274192 | 5.00E-64 | 733227 - 735245 | Homoserine O-acetyltransferase [Aspergillus clavatus]. |  |  |  | -2.45 |  |  |  | -4.257 |  |  |  | 1.308 |
|  | 1045200 - 1048100 | GLEAN_05775 | XP_570964 | 0.00E+00 | 1041966 - 1046131 | Actin cytoskeleton organization and biogenesis-related protein |  |  |  | -2.045 |  |  |  | -4.343 |  |  |  | 0.108 |
|  | 1045200 - 1048100 | GLEAN_05774 | XP_777918 | 2.00E-42 | 1046465 - 1048420 | Hypothetical protein |  |  |  | -2.045 |  |  |  | -4.343 |  |  |  | 0.108 |
|  | 1415300 - 1420100 | GLEAN_05698 | CAL55333 | 5.00E-76 | 1416143 - 1418177 | Myc-regulated DEAD/H box 18 RNA helicase-like (ISS) [Ostreococcus tauri]. |  |  | -2.961 |  |  |  | -4.886 |  |  |  | 0.166 |  |
|  | 1415300 - 1420100 | GLEAN_06239 | XP_775062 | 7.00E-75 | 1420795 - 1421725 | Hypothetical protein |  |  | -2.961 |  |  |  | -4.886 |  |  |  | 0.166 |  |
|  |  |  |  |  |  |  |  |  |  |  |  |  |  |  |  |  |  |  |
| 7 | 6600 - 10600 | GLEAN_00262 | XP_001268434 | 2.00E-06 | 7512 - 10013 | Ferric-chelate reductase, putative [Aspergillus clavatus]. |  |  |  | -2.724 |  |  |  | -4.379 |  |  |  | 0.093 |
|  | 142700 - 144900 | NO GENE | NO GENE |  |  |  |  |  |  | -2.49 |  |  |  | -4.728 |  |  |  | 0.18 |
|  | 383600 - 395900 | GLEAN_00330 | XP_571655 | 2.00E-117 | 382103 - 384126 | Rtf1 protein |  |  |  | -2.778 |  |  |  | -4.832 |  |  |  | 0.326 |
|  | 383600 - 395900 | GLEAN_00200 | XP_001258574 | 7.00E-18 | 384584 - 385725 | Haloalkanoic acid dehalogenase, putative [Neosartorya fischeri]. |  |  |  | -2.778 |  |  |  | -4.832 |  |  |  | 0.326 |
|  | 383600 - 395900 | GLEAN_00199 | ABF72274 | 3.20E+00 | 386801 - 387565 | HSV-1 UL36-like protein [Gallid herpesvirus 2]. |  |  |  | -2.778 |  |  |  | -4.832 |  |  |  | 0.326 |
|  | 383600 - 395900 | GLEAN_00331 | ZP_01509465 | 4.00E-57 | 388229 - 389873 | Amidohydrolase [Burkholderia phytofirmans]. |  |  |  | -2.778 |  |  |  | -4.832 |  |  |  | 0.326 |
|  | 383600 - 395900 | GLEAN_00198 | XP_001260343 | 1.00E-55 | 390974 - 392935 | MFS allantoate transporter, putative [Neosartorya fischeri]. |  |  |  | -2.778 |  |  |  | -4.832 |  |  |  | 0.326 |
|  | 383600 - 395900 | GLEAN_00332 | YP_833124 | 2.00E-10 | 393793 - 394439 | Hypothetical protein [Arthrobacter sp. FB24]. |  |  |  | -2.778 |  |  |  | -4.832 |  |  |  | 0.326 |
|  | 383600 - 395900 | GLEAN_00333 | XP_571657 | 5.00E-78 | 394832 - 395588 | Hypothetical protein |  |  |  | -2.778 |  |  |  | -4.832 |  |  |  | 0.326 |
|  | 383600 - 395900 | GLEAN_00197 | XP_571632 | 5.00E-175 | 395779 - 397255 | GPI-anchor transamidase [ |  |  |  | -2.778 |  |  |  | -4.832 |  |  |  | 0.326 |
|  | 384000 - 395400 | GLEAN_00330 | XP_571655 | 2.00E-117 | 382103 - 384126 | Rtf1 protein | -3.14 |  |  |  | -4.826 |  |  |  | 0.907 |  |  |  |
|  | 384000 - 395400 | GLEAN_00200 | XP_777454 | 3.00E-31 | 384584 - 385725 | Hypothetical protein | -3.14 |  |  |  | -4.826 |  |  |  | 0.907 |  |  |  |
|  | 384000 - 395400 | GLEAN_00199 | ABF72274 | 3.20E+00 | 386801 - 387565 | HSV-1 UL36-like protein [Gallid herpesvirus 2]. | -3.14 |  |  |  | -4.826 |  |  |  | 0.907 |  |  |  |
|  | 384000 - 395400 | GLEAN_00331 | XP_001217242 | 8.00E-86 | 388229 - 389873 | Conserved hypothetical protein [Aspergillus terreus]. | -3.14 |  |  |  | -4.826 |  |  |  | 0.907 |  |  |  |
|  | 384000 - 395400 | GLEAN_00198 | XP_001260343 | 1.00E-55 | 390974 - 392935 | MFS allantoate transporter, putative [Neosartorya fischeri]. | -3.14 |  |  |  | -4.826 |  |  |  | 0.907 |  |  |  |
|  | 384000 - 395400 | GLEAN_00332 | YP_833124 | 2.00E-10 | 393793 - 394439 | Hypothetical protein [Arthrobacter sp. FB24]. | -3.14 |  |  |  | -4.826 |  |  |  | 0.907 |  |  |  |
|  | 384000 - 395400 | GLEAN_00333 | XP_571657 | 5.00E-78 | 394832 - 395588 | Hypothetical protein | -3.14 |  |  |  | -4.826 |  |  |  | 0.907 |  |  |  |
|  | 425600 - 426800 | GLEAN_00192 | XP_571429 | 2.00E-171 | 425706 - 427378 | Hypothetical protein |  | -0.716 |  |  |  | -2.739 |  |  |  |  |  |  |
|  | 586000 - 586800 | GLEAN_00166 | BAD23582 | 7.00E-44 | 586777 - 588429 | Putative nicotianamine aminotransferase A [Oryza sativa] | -0.92 |  |  |  | -1.511 |  |  |  | 0.043 | 0.329 |  |  |
|  | 673600 - 676000 | GLEAN_00148 | YP_081854 | 3.20E+00 | 672933 - 673705 | Chaperonin GroEL [Bacillus cereus]. |  | 0.471 |  |  |  | -0.354 |  |  |  |  |  |  |
|  | 724700 - 725200 | GLEAN_00399 | P19711 | 6.40E-01 | 724939 - 729221 | Polyprotein protease/helicase |  | 0.944 |  |  |  | 0.277 |  |  |  | 0.939 |  |  |
|  | 982800 - 983600 | GLEAN_00441 | EAU91579 | 9.00E-121 | 979312 - 983768 | Hypothetical protein [Coprinopsis cinerea]. |  |  |  |  |  |  |  |  |  | 1.919 |  |  |
|  | 1002500 - 1012100 | GLEAN_00443 | XP_571285 | 3.00E-151 | 1005135 - 1006425 | Alpha-1,6-mannosyltransferase |  |  |  | -1.918 |  |  |  | -3.467 |  |  |  | 0.417 |
|  | 1002500 - 1012100 | GLEAN_00081 | XP_571582 | 5.00E-40 | 1006673 - 1007113 | Hypothetical protein |  |  |  | -1.918 |  |  |  | -3.467 |  |  |  | 0.417 |
|  | 1002500 - 1012100 | GLEAN_00444 | XP_571557 | 0.00E+00 | 1008850 - 1011921 | GTPase activating protein [ |  |  |  | -1.918 |  |  |  | -3.467 |  |  |  | 0.417 |
|  | 1018900 - 1020800 | GLEAN_00446 | XP_571276 | 0.00E+00 | 1016998 - 1019119 | Cytochrome P450 |  |  |  | -1.044 |  |  |  | -2.34 |  |  |  | 0.401 |
|  | 1018900 - 1020800 | GLEAN_00447 | NP_001040379 | 3.00E-20 | 1020300 - 1022266 | mRNA cap-binding protein eIF4E [Bombyx mori]. |  |  |  | -1.044 |  |  |  | -2.34 |  |  |  | 0.401 |
|  | 1100700 - 1102000 | GLEAN_00467 | XP_571251 | 5.00E-166 | 1105573 - 1108232 | Glycosyl transferase |  |  |  | -1.193 |  |  |  | -2.231 |  |  |  | 0.371 |
|  | 1128000 - 1131000 | GLEAN_00052 | XP_774711 | 3.00E-91 | 1127189 - 1129177 | Hypothetical protein |  |  |  | -0.687 |  |  |  | -1.597 |  |  |  | 0.126 |
|  | 1128000 - 1131000 | GLEAN_00051 | YP_001108517 | 2.00E-15 | 1129581 - 1130772 | Phosphoglycerate mutase family protein [Saccharopolyspora erythraea]. |  |  |  | -0.687 |  |  |  | -1.597 |  |  |  | 0.126 |
|  | 1166600 - 1167900 | NO GENE | NO GENE |  |  |  |  |  |  | -1.148 |  |  |  | -2.874 |  |  |  | 0.212 |
|  | 1188000 - 1196000 | GLEAN_00040 | XP_772401 | 3.00E-138 | 1189465 - 1193489 | Hypothetical protein | -1.613 | -1.866 |  |  | -4.192 | -5.28 |  |  | 0.772 |  |  |  |
|  | 1188000 - 1196000 | GLEAN_00477 | ABA94155 | 2.40E+00 | 1194262 - 1195759 | Expressed protein [Oryza sativa] | -1.613 | -1.866 |  |  | -4.192 | -5.28 |  |  | 0.772 | 0.213 |  |  |
|  | 1189100 - 1192900 | GLEAN_00040 | XP_772401 | 3.00E-138 | 1189465 - 1193489 | Hypothetical protein |  |  |  | 0.868 |  |  |  | -0.156 |  |  |  | 1.852 |
|  | 1262200 - 1280000 | GLEAN_00026 | XP_715482 | 1.00E-176 | 1256635 - 1261872 | RNA polymerase III large subunit [Candida albicans]. |  | -0.612 |  |  |  | -2.656 |  |  |  | 0.213 |  |  |
|  | 1262200 - 1280000 | GLEAN_00025 | XP_568378 | 2.00E-122 | 1262534 - 1263576 | Hypothetical protein |  | -0.612 |  |  |  | -2.656 |  |  |  | 1.513 |  |  |
|  | 1262200 - 1280000 | GLEAN_00489 | XP_772260 | 3.00E-14 | 1265210 - 1265557 | Hypothetical protein |  | -0.612 |  |  |  | -2.656 |  |  |  | 1.513 |  |  |
|  | 1262200 - 1280000 | GLEAN_00024 | XP_774752 | 1.00E-37 | 1266928 - 1267252 | Hypothetical protein |  | -0.612 |  |  |  | -2.656 |  |  |  | 1.513 |  |  |
|  | 1262200 - 1280000 | GLEAN_00023 | XP_571067 | 3.00E-04 | 1268789 - 1268917 | Hypothetical protein |  | -0.612 |  |  |  | -2.656 |  |  |  | 1.513 |  |  |
|  | 1262200 - 1280000 | GLEAN_00490 | XP_001260553 | 4.00E-24 | 1273360 - 1275465 | C2H2 transcription factor (Con7), putative [Neosartorya fischeri]. |  | -0.612 |  |  |  | -2.656 |  |  |  | 1.513 |  |  |
|  | 1262200 - 1280000 | GLEAN_00491 | XP_568378 | 3.00E-129 | 1276325 - 1277391 | Hypothetical protein |  | -0.612 |  |  |  | -2.656 |  |  |  | 1.513 |  |  |
|  | 1367600 - 1369800 | GLEAN_00004 | BAD31398 | 3.20E+00 | 1367766 - 1368428 | Hypothetical protein [Oryza sativa] |  |  |  | -1.702 |  |  |  | -3.171 |  |  |  | 0.064 |
|  | 1396800 - 1399489 | GLEAN_00513 | XP_383545 | 2.00E-146 | 1395135 - 1396994 | Hypothetical protein [Gibberella zeae]. | -2.408 |  |  |  | -1.985 |  |  |  | 1.701 | 1.513 |  |  |
|  |  |  |  |  |  |  |  |  |  |  |  |  |  |  |  |  |  |  |
| 8 | 0 - 14000 | GLEAN_06517 | XP_776387 | 8.00E-59 | 4033 - 6312 | Hypothetical protein | -1.282 |  |  |  | -4.162 |  |  |  | 1.267 |  |  |  |
|  | 0 - 14000 | GLEAN_06516 | XP_776539 | 1.00E-28 | 10046 - 12151 | Hypothetical protein | -1.282 |  |  |  | -4.162 |  |  |  | 1.267 |  |  |  |
|  | 0 - 14000 | GLEAN_06515 | XP_746529 | 4.00E-43 | 13769 - 14323 | L-PSP endoribonuclease family protein, putative [Aspergillus fumigatus]. | -1.282 |  |  |  | -4.162 |  |  |  | 1.267 |  |  |  |
|  | 4000 - 33700 | GLEAN_06517 | XP_776387 | 8.00E-59 | 4033 - 6312 | Hypothetical protein |  |  |  | -2.84 |  |  |  | -4.66 |  |  |  | 0.346 |
|  | 4000 - 33700 | GLEAN_06516 | XP_776539 | 1.00E-28 | 10046 - 12151 | Hypothetical protein |  |  |  | -2.84 |  |  |  | -4.66 |  |  |  | 0.346 |
|  | 4000 - 33700 | GLEAN_06515 | XP_746529 | 4.00E-43 | 13769 - 14323 | L-PSP endoribonuclease family protein, putative [Aspergillus fumigatus]. |  |  |  | -2.84 |  |  |  | -4.66 |  |  |  | 0.346 |
|  | 4000 - 33700 | GLEAN_06518 | CAM37292 | 3.10E+00 | 15320 - 15848 | Hypothetical protein, conserved [Leishmania braziliensis]. |  |  |  | -2.84 |  |  |  | -4.66 |  |  |  | 0.346 |
|  | 4000 - 33700 | GLEAN_06519 | XP_001262470 | 1.00E-11 | 16360 - 18636 | Fungal specific transcription factor, putative [Neosartorya fischeri]. |  |  |  | -2.84 |  |  |  | -4.66 |  |  |  | 0.346 |
|  | 4000 - 33700 | GLEAN_06514 | XP_751072 | 2.00E-37 | 19161 - 21270 | MFS alpha-glucoside transporter, putative [Aspergillus fumigatus]. |  |  |  | -2.84 |  |  |  | -4.66 |  |  |  | 0.346 |
|  | 4000 - 33700 | GLEAN_06520 | ZP_00767178 | 1.00E-16 | 21436 - 21865 | Glycoside hydrolase [Chloroflexus aurantiacus]. |  |  |  | -2.84 |  |  |  | -4.66 |  |  |  | 0.346 |
|  | 4000 - 33700 | GLEAN_06521 | ZP_01531626 | 9.00E-56 | 22016 - 23958 | Glycoside hydrolase [Roseiflexus castenholzii]. |  |  |  | -2.84 |  |  |  | -4.66 |  |  |  | 0.346 |
|  | 4000 - 33700 | GLEAN_06513 | XP_572141 | 1.00E-28 | 24519 - 26490 | Efflux protein EncT |  |  |  | -2.84 |  |  |  | -4.66 |  |  |  | 0.346 |
|  | 4000 - 33700 | GLEAN_06512 | XP_569475 | 2.00E-06 | 28052 - 29426 | Integral membrane protein |  |  |  | -2.84 |  |  |  | -4.66 |  |  |  | 0.346 |
|  | 4000 - 33700 | GLEAN_06511 | XP_777695 | 2.00E-56 | 30227 - 32238 | Hypothetical protein |  |  |  | -2.84 |  |  |  | -4.66 |  |  |  | 0.346 |
|  | 14000 - 36000 | GLEAN_06518 | CAM37292 | 3.10E+00 | 15320 - 15848 | Hypothetical protein, conserved [Leishmania braziliensis]. | -3.562 |  |  |  | -4.852 |  |  |  | -0.243 |  |  |  |
|  | 14000 - 36000 | GLEAN_06519 | XP_001262470 | 1.00E-11 | 16360 - 18636 | Fungal specific transcription factor, putative [Neosartorya fischeri]. | -3.562 |  |  |  | -4.852 |  |  |  | -0.243 |  |  |  |
|  | 14000 - 36000 | GLEAN_06514 | XP_751072 | 2.00E-37 | 19161 - 21270 | MFS alpha-glucoside transporter, putative [Aspergillus fumigatus]. | -3.562 |  |  |  | -4.852 |  |  |  | -0.243 |  |  |  |
|  | 14000 - 36000 | GLEAN_06520 | ZP_00767178 | 1.00E-16 | 21436 - 21865 | Glycoside hydrolase [Chloroflexus aurantiacus]. | -3.562 |  |  |  | -4.852 |  |  |  | -0.243 |  |  |  |
|  | 14000 - 36000 | GLEAN_06521 | ZP_01531626 | 9.00E-56 | 22016 - 23958 | Glycoside hydrolase [Roseiflexus castenholzii]. | -3.562 |  |  |  | -4.852 |  |  |  | -0.243 |  |  |  |
|  | 14000 - 36000 | GLEAN_06513 | XP_572141 | 1.00E-28 | 24519 - 26490 | Efflux protein EncT | -3.562 |  |  |  | -4.852 |  |  |  | -0.243 |  |  |  |
|  | 14000 - 36000 | GLEAN_06512 | XP_681373 | 2.00E-07 | 28052 - 29426 | Hypothetical protein AN8104.2 [Aspergillus nidulans]. | -3.562 |  |  |  | -4.852 |  |  |  | -0.243 |  |  |  |
|  | 14000 - 36000 | GLEAN_06511 | XP_777695 | 2.00E-56 | 30227 - 32238 | Hypothetical protein | -3.562 |  |  |  | -4.852 |  |  |  | -0.243 |  |  |  |
|  | 14000 - 36000 | GLEAN_06522 | XP_773071 | 4.00E-173 | 34189 - 35782 | Hypothetical protein | -3.562 |  |  |  | -4.852 |  |  |  | -0.243 |  |  |  |
|  | 33700 - 37400 | GLEAN_06522 | XP_773071 | 4.00E-173 | 34189 - 35782 | Hypothetical protein |  |  |  | 0.945 |  |  |  | -0.982 |  |  |  | 2.333 |
|  | 33700 - 37400 | GLEAN_06510 | XP_571050 | 6.00E-47 | 37464 - 38143 | Hypothetical protein |  |  |  | 0.945 |  |  |  | -0.982 |  |  |  | 2.333 |
|  | 36000 - 37800 | GLEAN_06510 | XP_571050 | 6.00E-47 | 37464 - 38143 | Hypothetical protein | -1.489 |  |  |  | -2.315 |  |  |  | -0.324 |  |  |  |
|  | 453100 - 458500 | GLEAN_06597 | XP_568316 | 5.00E-36 | 453515 - 454111 | Hypothetical protein |  |  |  | -1.301 |  |  |  | -2.987 |  |  |  | 0.263 |
|  | 453100 - 458500 | GLEAN_06598 | ABA98785 | 9.00E-29 | 454935 - 456752 | Retrotransposon protein [Oryza sativa]. |  |  |  | -1.301 |  |  |  | -2.987 |  |  |  | 0.263 |
|  | 453100 - 458500 | GLEAN_06599 | AAG50698 | 1.00E-40 | 456852 - 457928 | Copia-type polyprotein [Arabidopsis thaliana]. |  |  |  | -1.301 |  |  |  | -2.987 |  |  |  | 0.263 |
|  | 453100 - 458500 | GLEAN_06600 | BAD34493 | 1.00E-32 | 458200 - 458805 | Gag-Pol [Ipomoea batatas]. |  |  |  | -1.301 |  |  |  | -2.987 |  |  |  | 0.263 |
|  | 453600 - 458400 | GLEAN_06597 | XP_568316 | 5.00E-36 | 453515 - 454111 | Hypothetical protein |  |  | -1.399 |  |  |  | -2.987 |  |  |  | 0.263 |  |
|  | 454000 - 457700 | GLEAN_06597 | XP_568316 | 5.00E-36 | 453515 - 454111 | Hypothetical protein | 0.889 | 0.755 |  |  | -0.697 | -1.178 |  |  | 2.459 |  |  |  |
|  | 454000 - 457700 | GLEAN_06598 | ABA98785 | 9.00E-29 | 454935 - 456752 | Retrotransposon protein, Ty1-copia subclass [Oryza sativa] | 0.889 | 0.755 |  |  | -0.697 | -1.178 |  |  | 2.459 | 2.108 |  |  |
|  | 454000 - 457700 | GLEAN_06599 | AAG50698 | 1.00E-40 | 456852 - 457928 | Copia-type polyprotein [Arabidopsis thaliana]. | 0.889 | 0.755 |  |  | -0.697 | -1.178 |  |  | 2.459 | 2.108 |  |  |
|  | 521600 - 522400 | NO GENE | NO GENE |  |  |  | -0.737 |  |  |  | -2.322 |  |  |  | 0.897 | 2.108 |  |  |
|  | 654200 - 655200 | GLEAN_06637 | XP_761106 | 3.00E-123 | 651656 - 654505 | Hypothetical protein [Ustilago maydis]. | -1.819 |  |  |  | -3.227 |  |  |  | 0.428 |  |  |  |
|  | 717500 - 718600 | NO GENE | NO GENE |  |  |  | -1.287 |  |  |  | -2.397 |  |  |  | -0.132 |  |  |  |
|  | 1044400 - 1045600 | GLEAN_06710 | AAO92638 | 2.00E-134 | 1045012 - 1046596 | Putative transposase | -1.122 |  |  |  | -2.444 |  |  |  | 0.588 |  |  |  |
|  | 1049700 - 1051700 | GLEAN_06305 | XP_572141 | 3.00E-61 | 1049445 - 1051172 | Efflux protein EncT |  |  |  | -2.814 |  |  |  | -4.721 |  |  |  | 0.463 |
|  | 1059800 - 1066300 | NO GENE | NO GENE |  |  |  |  |  | -0.97 |  |  |  | -2.09 |  |  |  | 0.075 |  |
|  | 1059800 - 1066300 | GLEAN_06712 | XP_772401 | 9.00E-136 | 1061916 - 1065914 | Hypothetical protein |  |  | -0.97 |  |  |  | -2.09 |  |  |  | 0.075 |  |
|  | 1060500 - 1066300 | NO GENE | NO GENE |  |  |  | -2.218 |  |  |  | -4.349 |  |  |  | 0.665 |  |  |  |
|  | 1060500 - 1066300 | GLEAN_06712 | XP_772401 | 9.00E-136 | 1061916 - 1065914 | Hypothetical protein | -2.218 |  |  |  | -4.349 |  |  |  | 0.665 |  |  |  |
|  | 1059800 - 1066300 | NO GENE | NO GENE |  |  |  |  | -2.495 |  |  |  | -5.417 |  |  |  |  |  |  |
|  | 1059800 - 1066300 | GLEAN_06712 | XP_772401 | 9.00E-136 | 1061916 - 1065914 | Hypothetical protein |  | -2.495 |  |  |  | -5.417 |  |  |  | 0.416 |  |  |
|  |  |  |  |  |  |  |  |  |  |  |  |  |  |  |  |  |  |  |
| 9 | 1300 - 8000 | GLEAN_03978 | XP_772644 | 1.00E-120 | 1159 - 4637 | Hypothetical protein | -1.678 |  |  |  | -4.234 |  |  |  | 0.523 |  |  |  |
|  | 1300 - 8000 | GLEAN_03977 | XP_001210248 | 6.00E-30 | 5739 - 7035 | Conserved hypothetical protein [Aspergillus terreus]. | -1.678 |  |  |  | -4.234 |  |  |  | 0.523 |  |  |  |
|  | 1300 - 4400 | GLEAN_03978 | XP_772644 | 1.00E-120 | 1159 - 4637 | Hypothetical protein |  | 1.386 |  |  |  | -0.71 |  |  |  |  |  |  |
|  | 5000 - 7400 | GLEAN_03977 | XP_001210248 | 6.00E-30 | 5739 - 7035 | Conserved hypothetical protein [Aspergillus terreus]. |  |  |  | -2.576 |  |  |  | -4.139 |  |  |  | -0.013 |
|  | 11000 - 17700 | GLEAN_03976 | XP_747218 | 7.00E-26 | 11549 - 13695 | DUF895 domain membrane protein [Aspergillus fumigatus]. |  |  |  | -2.825 |  |  |  | -4.456 |  |  |  | -0.057 |
|  | 11000 - 17700 | GLEAN_03980 | ZP_01000094 | 4.00E-167 | 14230 - 16090 | Mucin-desulfating sulfatase (N-acetylglucosamine-6-sulfatase) [Oceanicola batsensis]. |  |  |  | -2.825 |  |  |  | -4.456 |  |  |  | -0.057 |
|  | 11000 - 17700 | GLEAN_03975 | XP_567067 | 1.70E+00 | 17638 - 17871 | Hypothetical protein |  |  |  | -2.825 |  |  |  | -4.456 |  |  |  | -0.057 |
|  | 32200 - 34700 | NO GENE | NO GENE |  |  |  |  |  |  | 1.232 |  |  |  | -0.158 |  |  |  | 3.205 |
|  | 32200 - 34700 | GLEAN_03984 | YP_421819 | 9.00E-24 | 33861 - 35408 | Aldo/keto reductase [Magnetospirillum magneticum]. |  |  |  | 1.232 |  |  |  | -0.158 |  |  |  | 3.205 |
|  | 74000 - 76000 | GLEAN_03962 | XP_573019 | 3.00E-87 | 73847 - 74740 | Hypothetical protein |  | 0.385 |  |  |  | -0.473 |  |  |  | 2.187 |  |  |
|  | 74000 - 76000 | GLEAN_03991 | EAU86881 | 3.30E-02 | 75333 - 77198 | Predicted protein [Coprinopsis cinerea]. |  | 0.385 |  |  |  | -0.473 |  |  |  | 1.146 |  |  |
|  | 290800 - 292600 | GLEAN_04033 | EAU91035 | 1.00E-113 | 292512 - 294915 | Hypothetical protein [Coprinopsis cinerea]. | -1.536 |  |  |  | -3.05 |  |  |  | 0.929 | 1.146 |  |  |
|  | 718000 - 718800 | GLEAN_04123 | XP_572808 | 1.00E-34 | 718424 - 719134 | Trafficking-related protein |  | -1.607 |  |  |  | -3.028 |  |  |  |  |  |  |
|  | 1038400 - 1042100 | GLEAN_03774 | XP_774001 | 5.00E-138 | 1040389 - 1043142 | Hypothetical protein | -0.765 |  |  |  | -2.222 |  |  |  | 1.052 | 0.115 |  |  |
|  | 1063600 - 1066100 | GLEAN_03769 | XP_964702 | 2.00E-09 | 1063408 - 1064053 | Hypothetical protein [Neurospora crassa]. | -3.267 |  |  |  | -4.397 |  |  |  | -1.626 |  |  |  |
|  | 1063600 - 1066100 | GLEAN_03768 | XP_964702 | 3.30E-01 | 1064105 - 1065009 | Hypothetical protein [Neurospora crassa]. | -3.267 |  |  |  | -4.397 |  |  |  | -1.626 |  |  |  |
|  | 1081700 - 1082800 | NO GENE | NO GENE |  |  |  | -2.192 |  |  |  | -3.29 |  |  |  | -0.386 |  |  |  |
|  | 1170200 - 1186500 | GLEAN_04209 | XP_772644 | 2.00E-120 | 1173012 - 1174844 | Hypothetical protein |  |  |  | -2.607 |  |  |  | -4.127 |  |  |  | -0.481 |
|  | 1170200 - 1186500 | GLEAN_04210 | XP_001257273 | 2.00E-09 | 1175586 - 1180935 | DEAD/DEAH box helicase, putative [Neosartorya fischeri]. |  |  |  | -2.607 |  |  |  | -4.127 |  |  |  | -0.481 |
|  | 1170200 - 1186500 | GLEAN_04211 | XP_775062 | 2.00E-42 | 1182351 - 1183092 | Hypothetical protein |  |  |  | -2.607 |  |  |  | -4.127 |  |  |  | -0.481 |
|  | 1170200 - 1186500 | GLEAN_04212 | XP_571065 | 4.10E-02 | 1184099 - 1184272 | Hypothetical protein |  |  |  | -2.607 |  |  |  | -4.127 |  |  |  | -0.481 |
|  | 1170200 - 1186500 | GLEAN_04213 | XP_775062 | 4.00E-49 | 1184512 - 1184954 | Hypothetical protein |  |  |  | -2.607 |  |  |  | -4.127 |  |  |  | -0.481 |
|  |  |  |  |  |  |  |  |  |  |  |  |  |  |  |  |  |  |  |
| 10 | 1700 - 4000 | GLEAN_06977 | XP_001260919 | 5.00E-97 | 1908 - 3411 | MFS monosaccharide transporter, putative [Neosartorya fischeri |  | -2.517 |  |  |  | -4.695 |  |  |  |  |  |  |
|  | 18800 - 20200 | GLEAN_06972 | XP_569289 | 1.00E-72 | 19344 - 20375 | Hypothetical protein |  |  | 0.531 |  |  |  | 0.03 |  |  |  | 1.403 |  |
|  | 24800 - 26300 | NO GENE | NO GENE |  |  |  | -2.591 |  |  |  | -3.788 |  |  |  | 0.056 | 0.717 |  |  |
|  | 83100 - 84000 | GLEAN_06960 | XP_570319 | 5.00E-130 | 82310 - 84327 | MFS transporter | -1.958 |  |  |  | -3.366 |  |  |  | 0.686 |  |  |  |
|  | 581700 - 587000 | GLEAN_07088 | P10978 | 3.00E-86 | 582143 - 586513 | Retrovirus-related Pol polyprotein from transposon TNT 1-94 |  |  |  | -2.921 |  |  |  | -4.509 |  |  |  | -0.212 |
|  | 581600 - 587000 | GLEAN_07088 | P10978 | 3.00E-86 | 582143 - 586513 | Retrovirus-related Pol polyprotein from transposon TNT 1-94 |  |  | -3.198 |  |  |  | -4.967 |  |  |  | -0.11 |  |
|  | 806800 - 808000 | GLEAN_06825 | XP_567457 | 2.00E-154 | 808170 - 810351 | Hypothetical protein |  | -2.201 |  |  |  | -3.714 |  |  |  |  |  |  |
|  | 823800 - 824400 | NO GENE | NO GENE |  |  |  |  |  |  | -0.691 |  |  |  | -1.266 |  |  |  | -0.237 |
|  | 961700 - 963200 | NO GENE | NO GENE |  |  |  | -1.959 |  |  |  | -3.968 |  |  |  | 0.221 |  |  |  |
|  | 1031700 - 1048700 | GLEAN_06784 | YP_705633 | 4.00E-81 | 1029797 - 1032095 | Probable dimethylaniline monooxygenase (N-oxide-forming) [Rhodococcus sp. RHA1]. |  |  | -3.151 |  |  |  | -4.988 |  |  |  | -0.026 |  |
|  | 1031700 - 1048700 | GLEAN_07172 | XP_772566 | 4.00E-66 | 1034330 - 1037705 | Hypothetical protein |  |  | -3.151 |  |  |  | -4.988 |  |  |  | -0.026 |  |
|  | 1031700 - 1048700 | GLEAN_07174 | XP_572946 | 3.00E-09 | 1039203 - 1039490 | Polyadenylation factor 64 kDa subunit |  |  | -3.151 |  |  |  | -4.988 |  |  |  | -0.026 |  |
|  | 1031700 - 1048700 | GLEAN_06783 | XP_572773 | 7.00E-72 | 1041534 - 1043168 | Endoplasmic reticulum receptor |  |  | -3.151 |  |  |  | -4.988 |  |  |  | -0.026 |  |
|  | 1031700 - 1048700 | GLEAN_07176 | YP_001135576 | 3.00E-05 | 1046465 - 1047590 | Short-chain dehydrogenase/reductase SDR [Mycobacterium gilvum]. |  |  | -3.151 |  |  |  | -4.988 |  |  |  | -0.026 |  |
|  | 1053500 - 1059200 | GLEAN_07178 | XP_572474 | 3.00E-03 | 1053017 - 1053523 | Hypothetical protein |  | -0.754 |  |  |  | -2.149 |  |  |  | 1.178 |  |  |
|  | 1053500 - 1059200 | GLEAN_07179 | XP_772644 | 2.00E-58 | 1054504 - 1057596 | Hypothetical protein |  | -0.754 |  |  |  | -2.149 |  |  |  | 1.178 |  |  |
|  | 1055100 - 1059200 | GLEAN_07167 | XP_567269 | 1.00E-147 | 1004892 - 1006231 | rRNA (adenine-N6,N6-)-dimethyltransferase | -1.191 |  |  |  | -3.982 |  |  |  | 1.347 |  |  |  |
|  |  |  |  |  |  |  |  |  |  |  |  |  |  |  |  |  |  |  |
| 11 | 3000 - 9900 | GLEAN_01511 | XP_772644 | 1.00E-55 | 3630 - 5205 | Hypothetical protein |  |  | -3.086 |  |  |  | -4.668 |  |  |  | -0.136 |  |
|  | 3000 - 9900 | GLEAN_01510 | XP_775062 | 2.00E-42 | 6729 - 7470 | Hypothetical protein |  |  | -3.086 |  |  |  | -4.668 |  |  |  | -0.136 |  |
|  | 3000 - 9900 | GLEAN_01509 | XP_776387 | 2.00E-04 | 8577 - 9286 | Hypothetical protein |  |  | -3.086 |  |  |  | -4.668 |  |  |  | -0.136 |  |
|  | 3000 - 13900 | GLEAN_01511 | XP_772644 | 1.00E-55 | 3630 - 5205 | Hypothetical protein |  |  |  | -2.835 |  |  |  | -4.696 |  |  |  | 0.614 |
|  | 3000 - 13900 | GLEAN_01510 | XP_775062 | 2.00E-42 | 6729 - 7470 | Hypothetical protein |  |  |  | -2.835 |  |  |  | -4.696 |  |  |  | 0.614 |
|  | 3000 - 13900 | GLEAN_01509 | XP_776387 | 2.00E-04 | 8577 - 9286 | Hypothetical protein |  |  |  | -2.835 |  |  |  | -4.696 |  |  |  | 0.614 |
|  | 3000 - 13900 | GLEAN_01508 | XP_001261163 | 2.00E-37 | 11099 - 13034 | Sodium bile acid transporter family protein, putative [Neosartorya fischeri]. |  |  |  | -2.835 |  |  |  | -4.696 |  |  |  | 0.614 |
|  | 3000 - 13900 | GLEAN_01507 | XP_748091 | 5.00E-24 | 13611 - 17959 | GARP complex component (Vps54), putative [Aspergillus fumigatus]. |  |  |  | -2.835 |  |  |  | -4.696 |  |  |  | 0.614 |
|  | 8400 - 10000 | GLEAN_01509 | XP_776387 | 2.00E-04 | 8577 - 9286 | Hypothetical protein |  | 1.538 |  |  |  | 0.113 |  |  |  | 2.359 |  |  |
|  | 94700 - 96600 | GLEAN_01523 | ABE73177 | 9.00E-101 | 95209 - 95983 | Putative O-acetyl transferase [Cryptococcus neoformans var. grubii]. |  |  |  | -1.995 |  |  |  | -4.198 |  |  |  | 1.645 |
|  | 804000 - 805900 | GLEAN_01353 | XP_569551 | 9.00E-84 | 803478 - 804198 | Import inner membrane translocase subunit tim22 | -1.69 |  |  |  | -3.632 |  |  |  | 0.724 | 2.359 |  |  |
|  | 926800 - 931800 | GLEAN_01677 | AAZ28935 | 7.00E-03 | 926825 - 927363 | Polyprotein [Phanerochaete chrysosporium]. |  |  |  | -1.08 |  |  |  | -2.993 |  |  |  | 1.315 |
|  | 966600 - 968600 | GLEAN_01689 | XP_568729 | 9.00E-74 | 966140 - 966876 | Retrotransposable element slacs 132 kda protein | -1.233 |  |  |  | -3.374 |  |  |  | 0.135 |  |  |  |
|  | 966600 - 968600 | GLEAN_01690 | XP_572149 | 2.00E-69 | 967322 - 967825 | Hypothetical protein | -1.233 |  |  |  | -3.374 |  |  |  | 0.135 |  |  |  |
|  | 1040800 - 1042400 | GLEAN_01309 | XP_569994 | 2.00E-93 | 1038260 - 1041361 | Receptor, putative | -1.076 |  |  |  | -2.515 |  |  |  | 0.659 |  |  |  |
|  | 1040800 - 1042400 | GLEAN_01706 | XP_569644 | 7.00E-166 | 1042367 - 1044262 | Long-chain acyl-CoA synthetase | -1.076 |  |  |  | -2.515 |  |  |  | 0.659 |  |  |  |
|  | 1169300 - 1172500 | GLEAN_01285 | XP_567697 | 0.00E+00 | 1167426 - 1169655 | Protein-lysine N-methyltransferase |  |  |  | -2.221 |  |  |  | -4.159 |  |  |  | 0.253 |
|  | 1169300 - 1172500 | GLEAN_01284 | XP_772756 | 1.00E-45 | 1171247 - 1171820 | Hypothetical protein |  |  |  | -2.221 |  |  |  | -4.159 |  |  |  | 0.253 |
|  | 1281700 - 1282500 | NO GENE | NO GENE |  |  |  | -2.739 |  |  |  | -4.487 |  |  |  | -1.29 |  |  |  |
|  | 1386600 - 1390000 | GLEAN_01771 | XP_771821 | 2.00E-129 | 1387178 - 1388221 | Hypothetical protein | -2.615 |  |  |  | -3.395 |  |  |  | -0.619 |  |  |  |
|  | 1386600 - 1390000 | GLEAN_01244 | XP_771822 | 4.00E-21 | 1388488 - 1388664 | Hypothetical protein | -2.615 |  |  |  | -3.395 |  |  |  | -0.619 |  |  |  |
|  | 1386600 - 1390000 | GLEAN_01243 | XP_771822 | 2.00E-27 | 1389040 - 1389583 | Hypothetical protein | -2.615 |  |  |  | -3.395 |  |  |  | -0.619 |  |  |  |
|  | 1387800 - 1392200 | GLEAN_01771 | XP_771821 | 2.00E-129 | 1387178 - 1388221 | Hypothetical protein |  | -1.956 |  |  |  | -2.801 |  |  |  |  |  |  |
|  | 1387800 - 1392200 | GLEAN_01244 | XP_771822 | 4.00E-21 | 1388488 - 1388664 | Hypothetical protein |  | -1.956 |  |  |  | -2.801 |  |  |  | -0.814 |  |  |
|  | 1387800 - 1392200 | GLEAN_01243 | XP_771822 | 2.00E-27 | 1389040 - 1389583 | Hypothetical protein |  | -1.956 |  |  |  | -2.801 |  |  |  | -0.814 |  |  |
|  | 1387900 - 1394200 | GLEAN_01771 | XP_771821 | 2.00E-129 | 1387178 - 1388221 | Hypothetical protein |  |  |  | -2.987 |  |  |  | -3.916 |  |  |  | -0.509 |
|  | 1405200 - 1411700 | GLEAN_01240 | XP_001344503 | 2.90E-01 | 1404859 - 1405737 | hypothetical protein [Danio rerio]. | -2.858 |  |  |  | -4.834 |  |  |  | 1.003 |  |  |  |
|  | 1405200 - 1411700 | GLEAN_01239 | XP_759004 | 2.00E-24 | 1407095 - 1410234 | Hypothetical protein [Ustilago maydis]. | -2.858 |  |  |  | -4.834 |  |  |  | 1.003 |  |  |  |
|  | 1405600 - 1411400 | GLEAN_01240 | XP_001344503 | 2.90E-01 | 1404859 - 1405737 | hypothetical protein [Danio rerio]. |  |  |  | -3.225 |  |  |  | -4.7 |  |  |  | 0.049 |
|  | 1405600 - 1411400 | GLEAN_01239 | XP_759004 | 2.00E-24 | 1407095 - 1410234 | Hypothetical protein [Ustilago maydis]. |  |  |  | -3.225 |  |  |  | -4.7 |  |  |  | 0.049 |
|  | 1531200 - 1562000 | GLEAN_01219 | XP_567753 | 3.00E-18 | 1531328 - 1531639 | Hypothetical protein |  |  |  | -2.408 |  |  |  | -4.792 |  |  |  | 1.522 |
|  | 1531200 - 1562000 | GLEAN_01795 | XP_568995 | 8.00E-132 | 1532812 - 1535621 | Xenobiotic-transporting ATPase |  |  |  | -2.408 |  |  |  | -4.792 |  |  |  | 1.522 |
|  | 1531200 - 1562000 | GLEAN_01796 | XP_753711 | 5.00E-66 | 1539120 - 1541018 | DUF1479 domain protein [Aspergillus fumigatus]. |  |  |  | -2.408 |  |  |  | -4.792 |  |  |  | 1.522 |
|  | 1531200 - 1562000 | GLEAN_01797 | XP_776387 | 1.00E-62 | 1541783 - 1546004 | Hypothetical protein |  |  |  | -2.408 |  |  |  | -4.792 |  |  |  | 1.522 |
|  | 1531200 - 1562000 | GLEAN_01798 | XP_771818 | 9.00E-15 | 1549079 - 1549589 | Hypothetical protein |  |  |  | -2.408 |  |  |  | -4.792 |  |  |  | 1.522 |
|  | 1531200 - 1562000 | GLEAN_01800 | XP_772644 | 2.00E-120 | 1553482 - 1555314 | Hypothetical protein |  |  |  | -2.408 |  |  |  | -4.792 |  |  |  | 1.522 |
|  | 1531200 - 1562000 | GLEAN_01801 | XP_001257273 | 2.00E-09 | 1556056 - 1561225 | DEAD/DEAH box helicase, putative [Neosartorya fischeri]. |  |  |  | -2.408 |  |  |  | -4.792 |  |  |  | 1.522 |
|  |  |  |  |  |  |  |  |  |  |  |  |  |  |  |  |  |  |  |
| 12 | 0 - 12800 | GLEAN_03603 | XP_772644 | 2.00E-32 | 962 - 2141 | Hypothetical protein | -0.815 |  |  |  | -2.207 |  |  |  | 0.978 |  |  |  |
|  | 0 - 12800 | GLEAN_03602 | XP_773252 | 2.00E-122 | 7475 - 8494 | Hypothetical protein | -0.815 |  |  |  | -2.207 |  |  |  | 0.978 |  |  |  |
|  | 0 - 12800 | GLEAN_03601 | XP_776074 | 8.00E-36 | 12447 - 13498 | Hypothetical protein | -0.815 |  |  |  | -2.207 |  |  |  | 0.978 |  |  |  |
|  | 2900 - 12500 | GLEAN_03602 | XP_773252 | 2.00E-122 | 7475 - 8494 | Hypothetical protein |  |  |  | -1.85 |  |  |  | -3.817 |  |  |  | 1.791 |
|  | 2900 - 12500 | GLEAN_03601 | XP_776074 | 8.00E-36 | 12447 - 13498 | Hypothetical protein |  |  |  | -1.85 |  |  |  | -3.817 |  |  |  | 1.791 |
|  | 7300 - 12600 | GLEAN_03602 | XP_773252 | 2.00E-122 | 7475 - 8494 | Hypothetical protein |  |  | -0.563 |  |  |  | -1.108 |  |  |  | 0.099 |  |
|  | 7300 - 12600 | GLEAN_03601 | XP_776074 | 8.00E-36 | 12447 - 13498 | Hypothetical protein |  |  | -0.563 |  |  |  | -1.108 |  |  |  | 0.099 |  |
|  | 12800 - 16300 | GLEAN_03601 | XP_776074 | 8.00E-36 | 12447 - 13498 | Hypothetical protein |  |  | 0.618 |  |  |  | 0.175 |  |  |  | 1.532 |  |
|  | 12800 - 16300 | GLEAN_03600 | XP_771818 | 1.00E-83 | 13917 - 15485 | Hypothetical protein |  |  | 0.618 |  |  |  | 0.175 |  |  |  | 1.532 |  |
|  | 12800 - 16300 | GLEAN_03599 | XP_567148 | 7.00E-64 | 15532 - 16111 | Hypothetical protein |  |  | 0.618 |  |  |  | 0.175 |  |  |  | 1.532 |  |
|  | 15600 - 20000 | GLEAN_03599 | XP_567148 | 7.00E-64 | 15532 - 16111 | Hypothetical protein |  | -0.933 |  |  |  | -2.008 |  |  |  |  |  |  |
|  | 15600 - 20000 | GLEAN_03604 | NP_923271 | 4.30E+00 | 17145 - 19082 | Crp family transcriptional regulatory protein [Gloeobacter violaceus]. |  | -0.933 |  |  |  | -2.008 |  |  |  | 1.824 |  |  |
|  | 37000 - 40900 | GLEAN_03608 | XP_568372 | 0.00E+00 | 38318 - 41392 | Amino acid transporter |  | -2.974 |  | -3.164 |  | -5.037 |  | -4.568 |  | 1.824 |  | 0.004 |
|  | 102600 - 103800 | NO GENE | NO GENE |  |  |  |  | 1.29 |  |  |  | 0.536 |  |  |  | 0.375 |  |  |
|  | 160000 - 165600 | GLEAN_03574 | XP_567971 | 4.00E-85 | 159542 - 160129 | Retrotransposon nucleocapsid protein | -1.032 |  |  |  | -2.349 |  |  |  | 1.079 | 2.186 |  |  |
|  | 160000 - 165600 | GLEAN_03573 | XP_567971 | 0.00E+00 | 160470 - 161894 | Retrotransposon nucleocapsid protein | -1.032 |  |  |  | -2.349 |  |  |  | 1.079 |  |  |  |
|  | 160000 - 165600 | GLEAN_03572 | ABE82053 | 4.00E-32 | 163221 - 165320 | Integrase [Medicago truncatula]. | -1.032 |  |  |  | -2.349 |  |  |  | 1.079 |  |  |  |
|  | 160000 - 168300 | GLEAN_03574 | XP_567971 | 4.00E-85 | 159542 - 160129 | Retrotransposon nucleocapsid protein |  |  |  | -0.739 |  |  |  | -2.742 |  |  |  | 1.102 |
|  | 160000 - 168300 | GLEAN_03573 | XP_567971 | 0.00E+00 | 160470 - 161894 | Retrotransposon nucleocapsid protein |  |  |  | -0.739 |  |  |  | -2.742 |  |  |  | 1.102 |
|  | 160000 - 168300 | GLEAN_03572 | ABE82053 | 4.00E-32 | 163221 - 165320 | Integrase [Medicago truncatula]. |  |  |  | -0.739 |  |  |  | -2.742 |  |  |  | 1.102 |
|  | 160000 - 168300 | GLEAN_03571 | XP_569055 | 5.00E-109 | 165669 - 166418 | Hypothetical protein |  |  |  | -0.739 |  |  |  | -2.742 |  |  |  | 1.102 |
|  | 160000 - 168300 | GLEAN_03570 | XP_750151 | 4.00E-12 | 167371 - 171903 | Yip1 domain family [Aspergillus fumigatus]. |  |  |  | -0.739 |  |  |  | -2.742 |  |  |  | 1.102 |
|  | 188600 - 189800 | NO GENE | NO GENE |  |  |  | -1.659 |  |  |  | -3.617 |  |  |  | 0.513 |  |  |  |
|  | 196400 - 197800 | GLEAN_03566 | XP_568448 | 0.00E+00 | 194388 - 196624 | Hypothetical protein |  |  |  | -1.547 |  |  |  | -2.843 |  |  |  | -0.489 |
|  | 196400 - 197800 | GLEAN_03640 | XP_568339 | 2.00E-116 | 196765 - 197842 | Cytoplasmic protein |  |  |  | -1.547 |  |  |  | -2.843 |  |  |  | -0.489 |
|  | 254600 - 256400 | GLEAN_03650 | XP_568398 | 3.00E-130 | 256216 - 257443 | Delayed-type hypersensitivity antigen - related protein |  | -0.716 |  |  |  | -2.263 |  |  |  |  |  |  |
|  | 254700 - 257300 | GLEAN_03650 | XP_568398 | 3.00E-130 | 256216 - 257443 | Delayed-type hypersensitivity antigen - related protein | -2.905 |  |  |  | -4.475 |  |  |  | -0.711 | 0.328 |  |  |
|  | 258700 - 260400 | GLEAN_03552 | XP_568729 | 3.00E-104 | 258356 - 259020 | Retrotransposable element slacs 132 kda protein |  |  |  | 0.78 |  |  |  | -0.384 |  |  |  | 1.953 |
|  | 362200 - 365700 | GLEAN_03669 | XP_568435 | 3.00E-56 | 365657 - 366909 | Cardiolipin synthase | -2.681 |  |  |  | -4.773 |  |  |  | 0.304 |  |  |  |
|  | 403700 - 405100 | GLEAN_03526 | XP_001263452 | 6.00E-02 | 404657 - 405192 | Cyclin, N-terminal domain protein, putative [Neosartorya fischeri]. |  |  |  | -1.544 |  |  |  | -3.064 |  |  |  | 0.322 |
|  | 536700 - 537500 | GLEAN_03699 | XP_568308 | 2.00E-167 | 535169 - 537300 | Cytoplasmic protein | -1.609 |  |  |  | -2.2 |  |  |  | -0.896 |  |  |  |
|  | 544200 - 546900 | GLEAN_03500 | XP_568304 | 0.00E+00 | 540944 - 544319 | DNA unwinding-related protein | -2.486 |  |  |  | -4.317 |  |  |  | -0.274 |  |  |  |
|  | 544200 - 546900 | GLEAN_03499 | XP_568303 | 0.00E+00 | 546566 - 549439 | Origin recognition complex subunit 4 | -2.486 |  |  |  | -4.317 |  |  |  | -0.274 |  |  |  |
|  | 605600 - 607800 | GLEAN_03711 | XP_568501 | 1.00E-154 | 604177 - 605546 | Hypothetical protein |  |  | -3.177 |  |  |  | -4.205 |  |  |  | -2.033 |  |
|  | 605600 - 607800 | GLEAN_03493 | XP_772030 | 4.00E-71 | 605702 - 607346 | Hypothetical protein |  |  | -3.177 |  |  |  | -4.205 |  |  |  | -2.033 |  |
|  | 757300 - 774000 | GLEAN_03469 | XP_001265924 | 2.00E-05 | 756670 - 757367 | Conserved hypothetical protein [Neosartorya fischeri]. |  |  |  | -2.867 |  |  |  | -4.753 |  |  |  | 0.688 |
|  | 757300 - 774000 | GLEAN_03742 | XP_568421 | 0.00E+00 | 757763 - 759398 | Hypothetical protein |  |  |  | -2.867 |  |  |  | -4.753 |  |  |  | 0.688 |
|  | 757300 - 774000 | GLEAN_03744 | XP_775243 | 1.00E-43 | 761411 - 764308 | Hypothetical protein |  |  |  | -2.867 |  |  |  | -4.753 |  |  |  | 0.688 |
|  | 757300 - 774000 | GLEAN_03468 | XP_571044 | 8.00E-113 | 764509 - 768109 | Beta-glucosidase |  |  |  | -2.867 |  |  |  | -4.753 |  |  |  | 0.688 |
|  | 757300 - 774000 | GLEAN_03745 | XP_571045 | 1.00E-176 | 768794 - 770908 | Hexose transport-related protein |  |  |  | -2.867 |  |  |  | -4.753 |  |  |  | 0.688 |
|  | 757300 - 774000 | GLEAN_03467 | XP_572478 | 5.00E-53 | 773214 - 773909 | Hypothetical protein |  |  |  | -2.867 |  |  |  | -4.753 |  |  |  | 0.688 |
|  | 762100 - 774000 | GLEAN_03744 | XP_571043 | 2.00E-44 | 761411 - 764308 | Hypothetical protein |  | -1.022 |  |  |  | -3.584 |  |  |  |  |  |  |
|  | 762100 - 774000 | GLEAN_03468 | XP_571044 | 8.00E-113 | 764509 - 768109 | Beta-glucosidase |  | -1.022 |  |  |  | -3.584 |  |  |  | 0.135 |  |  |
|  | 762100 - 774000 | GLEAN_03745 | XP_571045 | 1.00E-176 | 768794 - 770908 | Hexose transport-related protein |  | -1.022 |  |  |  | -3.584 |  |  |  | 0.135 |  |  |
|  | 762100 - 774000 | GLEAN_03467 | XP_572478 | 5.00E-53 | 773214 - 773909 | Hypothetical protein |  | -1.022 |  |  |  | -3.584 |  |  |  | 0.135 |  |  |
|  | 770900 - 773900 | GLEAN_03745 | XP_571045 | 1.00E-176 | 768794 - 770908 | Hexose transport-related protein | -1.242 |  |  |  | -3.364 |  |  |  | 0.454 | 0.135 |  |  |
|  | 770900 - 773900 | GLEAN_03467 | XP_572478 | 5.00E-53 | 773214 - 773909 | Hypothetical protein | -1.242 |  |  |  | -3.364 |  |  |  | 0.454 |  |  |  |
|  |  |  |  |  |  |  |  |  |  |  |  |  |  |  |  |  |  |  |
| 13 | 0 - 12000 | GLEAN_01076 | XP_775062 | 5e-68 | 2118 - 2973 | Hypothetical protein CNBE5380 |  |  |  | -0.208 |  |  |  | -2.843 |  |  |  | 1.105 |
|  | 0 - 12000 | GLEAN_01075 | XP_772644 | 6e-39 | 5154 - 6579 | Hypothetical protein CNBK0180 |  |  |  | -0.208 |  |  |  | -2.843 |  |  |  | 1.105 |
|  | 0 - 12000 | GLEAN_01074 | XP_569289 | 7e-45 | 7582 - 8308 | Hypothetical protein CNB05440 |  |  |  | -0.208 |  |  |  | -2.843 |  |  |  | 1.105 |
|  | 0 - 12000 | GLEAN_01073 | XP_567148 | 2e-65 | 8355 - 8934 | Hypothetical protein CNA02120 |  |  |  | -0.208 |  |  |  | -2.843 |  |  |  | 1.105 |
|  | 0 - 12000 | GLEAN_01077 | EAU87769 | 2e-10 | 10061 - 10883 | Predicted protein [Coprinopsis cinerea okayama7#130]. |  |  |  | -0.208 |  |  |  | -2.843 |  |  |  | 1.105 |
|  | 12300 - 25300 | GLEAN_01078 | XP_568523 | 1.00E-91 | 12544 - 14859 | Alpha-glucoside:hydrogen symporter |  |  |  | -2.783 |  |  |  | -4.668 |  |  |  | 0.877 |
|  | 12300 - 25300 | GLEAN_01072 | XP_568522 | 0.00E+00 | 17420 - 19828 | Hydrolase |  |  |  | -2.783 |  |  |  | -4.668 |  |  |  | 0.877 |
|  | 12300 - 25300 | GLEAN_01079 | XP_568521 | 2.00E-17 | 20420 - 20693 | Hypothetical protein |  |  |  | -2.783 |  |  |  | -4.668 |  |  |  | 0.877 |
|  | 12300 - 25300 | GLEAN_01080 | XP_771829 | 1.00E-72 | 20791 - 21291 | Hypothetical protein |  |  |  | -2.783 |  |  |  | -4.668 |  |  |  | 0.877 |
|  | 12300 - 25300 | GLEAN_01081 | CAB91097 | 5.00E-08 | 21726 - 23579 | Dextranase [Penicillium funiculosum]. |  |  |  | -2.783 |  |  |  | -4.668 |  |  |  | 0.877 |
|  | 12300 - 25300 | GLEAN_01082 | XP_771855 | 3.00E-14 | 24667 - 25110 | Hypothetical protein |  |  |  | -2.783 |  |  |  | -4.668 |  |  |  | 0.877 |
|  | 52000 - 54300 | GLEAN_01066 | NP_105753 | 7.00E-18 | 52924 - 53790 | Arginase [Mesorhizobium loti]. |  |  |  | -2.069 |  |  |  | -3.824 |  |  |  | 1.794 |
|  | 125900 - 128300 | GLEAN_01098 | XP_776539 | 2.00E-35 | 126395 - 128221 | Hypothetical protein |  |  | -0.086 | -1.319 |  |  | -0.011 | -2.45 |  |  | 0.24 | -0.139 |
|  | 239500 - 240800 | GLEAN_01031 | XP_568604 | 0 | 234722 - 239916 | ABC transporter PMR5 |  |  |  | -0.306 |  |  |  | -1.322 |  |  |  | 0.925 |
|  | 366400 - 367600 | GLEAN_01001 | XP_771936 | 2.00E-67 | 366611 - 367074 | Hypothetical protein | -1.162 |  |  |  | -3.383 |  |  |  | -0.256 |  |  |  |
|  | 366400 - 367600 | GLEAN_01000 | XP_568561 | 2.00E-141 | 367556 - 368812 | Hypothetical protein | -1.162 |  |  |  | -3.383 |  |  |  | -0.256 |  |  |  |
|  | 367700 - 368800 | GLEAN_01000 | XP_568561 | 2.00E-141 | 367556 - 368812 | Hypothetical protein | 2.696 |  |  |  | -0.072 |  |  |  | 5.158 |  |  |  |
|  | 367200 - 368400 | GLEAN_01000 | XP_568561 | 2.00E-141 | 367556 - 368812 | Hypothetical protein |  | 1.763 |  |  |  | -0.7 |  |  |  |  |  |  |
|  | 367000 - 368400 | GLEAN_01001 | XP_771936 | 2.00E-67 | 366611 - 367074 | Hypothetical protein |  |  | 3.673 |  |  |  | 2.239 |  |  |  | 4.863 |  |
|  | 367000 - 368400 | GLEAN_01000 | XP_568561 | 2.00E-141 | 367556 - 368812 | Hypothetical protein |  |  | 3.673 |  |  |  | 2.239 |  |  |  | 4.863 |  |
|  | 367600 - 368600 | GLEAN_01001 | XP_771936 | 2.00E-67 | 366611 - 367074 | Hypothetical protein |  |  |  | 3.57 |  |  |  | 0.892 |  |  |  | 5.345 |
|  | 367600 - 368600 | GLEAN_01000 | XP_568561 | 2.00E-141 | 367556 - 368812 | Hypothetical protein |  |  |  | 3.57 |  |  |  | 0.892 |  |  |  | 5.345 |
|  | 742400 - 751800 | GLEAN_01215 | XP_572246 | 0 | 740312 - 742468 | Galactose transporter |  |  |  | -0.306 |  |  |  | -1.322 |  |  |  | 0.925 |
|  | 742400 - 751800 | GLEAN_00928 | EAU93688 | 6e-10 | 743693 - 744577 | Predicted protein [Coprinopsis cinerea okayama7#130]. |  |  |  | -0.306 |  |  |  | -1.322 |  |  |  | 0.925 |
|  | 742400 - 751800 | GLEAN_01216 | XP_567148 | 9e-51 | 745538 - 746088 | Hypothetical protein CNA02120 |  |  |  | -0.306 |  |  |  | -1.322 |  |  |  | 0.925 |
|  | 742400 - 751800 | GLEAN_01217 | XP_771818 | 2e-79 | 746620 - 747691 | Hypothetical protein CNBN1960 |  |  |  | -0.306 |  |  |  | -1.322 |  |  |  | 0.925 |
|  | 751900 - 755900 | GLEAN_01218 | XP_773252 | 3.00E-126 | 753303 - 754322 | Hypothetical protein |  |  |  | -2.528 |  |  |  | -3.838 |  |  |  | 0.085 |
|  |  |  |  |  |  |  |  |  |  |  |  |  |  |  |  |  |  |  |
| 14 | 500 - 3700 | GLEAN_03307 | XP_568523 | 5.00E-64 | 569 - 1671 | Alpha-glucoside:hydrogen symporter |  |  |  | -2.783 |  |  |  | -4.352 |  |  |  | 0.012 |
|  | 500 - 3700 | GLEAN_03308 | XP_001271197 | 3.00E-08 | 2566 - 5152 | C6 transcription factor (Mut3), putative [Aspergillus clavatus]. |  |  |  | -2.783 |  |  |  | -4.352 |  |  |  | 0.012 |
|  | 112400 - 113300 |  | NO GENE |  |  |  |  |  |  | -2.199 |  |  |  | -3.685 |  |  |  | -0.14 |
|  | 140300 - 149500 | GLEAN_03286 | XP_572237 | 6.00E-157 | 138145 - 140359 | Myo-inositol transporter 1 |  |  |  | -0.977 |  |  |  | -3.38 |  |  |  | 1.044 |
|  | 140300 - 149500 | GLEAN_03285 | ZP_01225340 | 2.00E-10 | 141431 - 142546 | 3-hydroxyacyl-CoA dehydrogenase, putative [marine gamma proteobacterium HTCC2207]. |  |  |  | -0.977 |  |  |  | -3.38 |  |  |  | 1.044 |
|  | 140300 - 149500 | GLEAN_03333 | YP_001103866 | 1.00E-20 | 143093 - 144297 | Transcriptional regulator, IclR family/regucalcin [Saccharopolyspora erythraea]. |  |  |  | -0.977 |  |  |  | -3.38 |  |  |  | 1.044 |
|  | 140300 - 149500 | GLEAN_03334 | XP_772566 | 0.00E+00 | 144801 - 148667 | Hypothetical protein |  |  |  | -0.977 |  |  |  | -3.38 |  |  |  | 1.044 |
|  | 202400 - 204500 | GLEAN_03275 | XP_572256 | 0.00E+00 | 198991 - 202766 | Rab guanyl-nucleotide exchange factor | -0.689 |  |  |  | -2.934 |  |  |  | 0.402 |  |  |  |
|  | 208500 - 209300 | NO GENE | NO GENE |  |  |  | -2.387 |  |  |  | -3.881 |  |  |  | -0.255 |  |  |  |
|  | 280400 - 286400 | GLEAN_03255 | XP_572287 | 0.00E+00 | 278537 - 280728 | Asparagine synthase (glutamine-hydrolyzing) | -0.978 |  |  |  | -4.071 |  |  |  | 1.304 |  |  |  |
|  | 280400 - 286400 | GLEAN_03351 | NP_862599 | 5.00E+00 | 283364 - 283840 | Mob protein [Sinorhizobium meliloti]. | -0.978 |  |  |  | -4.071 |  |  |  | 1.304 |  |  |  |
|  | 280400 - 286400 | GLEAN_03352 | XP_772473 | 2.00E-107 | 286201 - 287028 | Hypothetical protein | -0.978 |  |  |  | -4.071 |  |  |  | 1.304 |  |  |  |
|  | 280800 - 286000 | GLEAN_03255 | XP_572287 | 0.00E+00 | 278537 - 280728 | Asparagine synthase |  | -0.535 |  |  |  | -2.29 |  |  |  |  |  |  |
|  | 280800 - 286000 | GLEAN_03351 | NP_862599 | 5.00E+00 | 283364 - 283840 | Mob protein [Sinorhizobium meliloti]. |  | -0.535 |  |  |  | -2.29 |  |  |  | 0.57 |  |  |
|  | 281000 - 289100 | GLEAN_03351 | NP_862599 | 5.00E+00 | 283364 - 283840 | Mob protein [Sinorhizobium meliloti]. |  |  |  | -1.295 |  |  |  | -4.895 |  |  |  | 0.906 |
|  | 281000 - 289100 | GLEAN_03334 | XP_772566 | 0.00E+00 | 144801 - 148667 | Hypothetical protein |  |  |  | -1.295 |  |  |  | -4.895 |  |  |  | 0.906 |
|  | 281000 - 289100 | GLEAN_03353 | XP_572288 | 0.00E+00 | 288275 - 291148 | Transcription initiation factor TFIID |  |  |  | -1.295 |  |  |  | -4.895 |  |  |  | 0.906 |
|  | 307500 - 309300 | GLEAN_03356 | XP_775020 | 0.00E+00 | 307532 - 308923 | Hypothetical protein | 1.293 |  |  |  | 0.1 |  |  |  | 1.997 | 0.57 |  |  |
|  | 307700 - 309200 | GLEAN_03356 | XP_775020 | 0.00E+00 | 307532 - 308923 | Hypothetical protein |  | 0.351 |  |  |  | -0.313 |  |  |  | 0.952 |  |  |
|  | 351600 - 354800 | GLEAN_03364 | XP_772494 | 0.00E+00 | 351626 - 353014 | Hypothetical protein | -1.229 |  |  |  | -2.176 |  |  |  | 0.075 | 0.952 |  |  |
|  | 427300 - 430900 | GLEAN_03376 | XP_774642 | 7.00E-05 | 428908 - 429103 | Hypothetical protein | -0.783 |  |  |  | -2.014 |  |  |  | 0.844 |  |  |  |
|  | 445100 - 460400 | GLEAN_03379 | XP_572333 | 2.00E-148 | 437892 - 441787 | MMS2 | -2.163 |  |  |  | -3.852 |  |  |  | -0.138 |  |  |  |
|  | 445100 - 460400 | GLEAN_03380 | XP_569289 | 2.00E-75 | 442473 - 445023 | Hypothetical protein | -2.163 |  |  |  | -3.852 |  |  |  | -0.138 |  |  |  |
|  | 445100 - 460400 | GLEAN_03220 | ABE94506 | 3.00E-19 | 447918 - 448957 | CENP-B protein; Homeodomain-like [Medicago truncatula]. | -2.163 |  |  |  | -3.852 |  |  |  | -0.138 |  |  |  |
|  | 445100 - 460400 | GLEAN_03381 | XP_567971 | 3.00E-113 | 450536 - 453340 | Retrotransposon nucleocapsid protein | -2.163 |  |  |  | -3.852 |  |  |  | -0.138 |  |  |  |
|  | 445100 - 460400 | GLEAN_03382 | ABA99612 | 3.00E-20 | 455778 - 457770 | Retrotransposon protein [Oryza sativa]. | -2.163 |  |  |  | -3.852 |  |  |  | -0.138 |  |  |  |
|  | 445100 - 460400 | GLEAN_03383 | P10978 | 3.00E-57 | 458257 - 459256 | Retrovirus-related Pol polyprotein from transposon TNT 1-94 | -2.163 |  |  |  | -3.852 |  |  |  | -0.138 |  |  |  |
|  | 445500 - 460500 | GLEAN_03220 | ABE94506 | 3.00E-19 | 447918 - 448957 | CENP-B protein; Homeodomain-like [Medicago truncatula]. |  | -0.538 |  |  |  | -2.001 |  |  |  |  |  |  |
|  | 445500 - 460500 | GLEAN_03381 | XP_567971 | 3.00E-113 | 450536 - 453340 | Retrotransposon nucleocapsid protein |  | -0.538 |  |  |  | -2.001 |  |  |  | 0.072 |  |  |
|  | 445500 - 460500 | GLEAN_03382 | ABA99612 | 3.00E-20 | 455778 - 457770 | Retrotransposon protein, putative, unclassified [Oryza sativa]. |  | -0.538 |  |  |  | -2.001 |  |  |  | 0.072 |  |  |
|  | 445500 - 460500 | GLEAN_03383 | P10978 | 3.00E-57 | 458257 - 459256 | Retrovirus-related Pol polyprotein from transposon TNT 1-94 |  | -0.538 |  |  |  | -2.001 |  |  |  | 0.072 |  |  |
|  | 587300 - 591300 | GLEAN_03404 | XP_568361 | 1.00E-05 | 587004 - 587437 | Hypothetical protein |  |  |  | -0.716 |  |  |  | -2.475 |  |  |  | 0.417 |
|  | 587300 - 591300 | GLEAN_03191 | XP_776539 | 2.00E-33 | 588928 - 590733 | Hypothetical protein |  |  |  | -0.716 |  |  |  | -2.475 |  |  |  | 0.417 |
|  | 833300 - 834900 | GLEAN_03449 | XP_572452 | 0.00E+00 | 829500 - 833428 | Protein tyrosine/threonine phosphatase |  |  |  | -0.844 |  |  |  | -2.387 |  |  |  | 0.641 |
|  | 833300 - 834900 | GLEAN_03450 | XP_001271965 | 1.00E-19 | 834615 - 837025 | Transporter protein smf2 [Aspergillus clavatus]. |  |  |  | -0.844 |  |  |  | -2.387 |  |  |  | 0.641 |
|  | 913000 - 925200 | GLEAN_03464 | XP_001481488 | 3.80E+00 | 913868 - 914840 | Class V chitinase, putative [Aspergillus fumigatus]. | -1.616 |  |  | -1.7 | -4.113 |  |  | -4.351 | 0.695 |  |  | 1.045 |
|  | 913000 - 925200 | GLEAN_03465 | XP_776539 | 1.00E-27 | 916876 - 918702 | Hypothetical protein | -1.616 |  |  | -1.7 | -4.113 |  |  | -4.351 | 0.695 |  |  | 1.045 |
|  | 913000 - 925200 | GLEAN_03466 | XP_776387 | 5.00E-49 | 920609 - 923050 | Hypothetical protein | -1.616 |  |  | -1.7 | -4.113 |  |  | -4.351 | 0.695 |  |  | 1.045 |

^a^ Nucleotide coordinates of the segment identified by CGH.

^b^ Glean number for the H99 gene in the region based on the annotation at the Broad Institute.

^c^ GenBank ID of top BLAST (what type of BLAST)). The e value of the BLAST result is included in the following column.

^d^ Coordinates of the specific gene in the segment identified by CGH.

^e^ Functional information about the top BLAST hit. Note that the functional information is from the *C. neoformans* annotation of JEC21, if no organism name is given.
