# Supplementary material for: Comparative hybridization reveals extensive genome variation in the AIDS-associated pathogen Cryptococcus neoformans
Source: Genome Biol. 2008 Feb 22;9(2):R41. doi: 10.1186/gb-2008-9-2-r41 (PMC2374700; doi:10.1186/gb-2008-9-2-r41)
Supplement: Additional data file 5 — Presented is a table of the quantitative RT-PCR analysis of gene copy number relative to the H99 genome. [file gb-2008-9-2-r41-S5.doc]

| Additional data file 5. Quantitative RT-PCR analysis of gene copy number relative to the H99 genome | | | | | | | |
| --- | --- | --- | --- | --- | --- | --- | --- |
| Gene | Strain | Ct(Avg.) test gene  (SD) | Ct (Avg.) Actin  (SD) | ΔCt_gene_ (Avg. test^a^ gene – Avg. Actin) | ΔCt_ref_. (Avg. H99 gene– Avg. Actin) | ΔΔCt (Avg. ΔCt_gene_ –Avg. ΔCt_ref_. (H99) | Normalized gene copy number relative to H99  (2^-ΔΔCt^) |
| CNN00820 | H99 | 18.53 (0.03) | 19.41 (0.20) | -0.88 | -0.88 | 0.00 | 1.00 |
|  | JEC21 | 17.65 (0.03) | 18.49 (0.03) | -.84 | -0.88 | 0.04 | 0.97 |
|  | WM626 | 17.57 (0.01) | 19.28 (0.08) | -1.71 | -0.88 | -0.83 | 1.78 |
|  | CBS7779 | 17.43 (0.04) | 18.65 (0.02) | -1.22 | -0.88 | -0.34 | 1.27 |
|  |  |  |  |  |  |  |  |
| CNN01890 | H99 | 18.69 (0.06) | 19.41 (0.20) | -0.72 | -0.72 | 0.00 | 1.00 |
|  | JEC21 | 18.03 (0.03) | 18.49 (0.03) | -0.46 | -0.72 | 0.26 | 0.84 |
|  | WM626 | 17.77 (0.01) | 19.28 (0.08) | -1.52 | -0.72 | -0.80 | 1.74 |
|  | CBS7779 | 17.50 (0.04) | 18.65 (0.02) | -1.15 | -0.72 | -0.43 | 1.35 |
|  |  |  |  |  |  |  |  |
| SMG1^b^ | H99 | 19.05 (0.02) | 19.41 (0.20) | -0.36 | -0.36 | 0.00 | 1.00 |
|  | JEC21 | 17.24 (0.19) | 18.49 (0.03) | -1.25 | -0.36 | -0.89 | 1.85 |
|  | WM626 | 18.98 (0.03) | 19.28 (0.08) | -0.30 | -0.36 | 0.06 | 0.96 |
|  | CBS7779 | 18.37 (0.03) | 18.65 (0.02) | -0.28 | -0.36 | 0.08 | 0.95 |
|  |  |  |  |  |  |  |  |
| CNN02400 | H99 | 18.81 (0.04) | 19.41 (0.20) | -0.6 | -0.6 | 0.00 | 1.00 |
|  | JEC21 | 18.28 (0.04) | 18.49 (0.03) | -0.21 | -0.6 | 0.39 | 0.76 |
|  | WM626 | 17.79 (0.04) | 19.28 (0.08) | -1.49 | -0.6 | -0.89 | 1.85 |
|  | CBS7779 | 17.51 (0.05) | 18.65 (0.02) | -1.14 | -0.6 | -0.54 | 1.45 |
|  |  |  |  |  |  |  |  |

^a^Test is the strain shown on the left

^b^ *SMG1* is present in two copies in the genome of JEC21 and one copy in the genome of H99. This gene is present on chromosomes 8 and 12, and serves as a positive control for the detection of more than one copy of a gene.
