# Supplementary material for: Comparative hybridization reveals extensive genome variation in the AIDS-associated pathogen Cryptococcus neoformans
Source: Genome Biol. 2008 Feb 22;9(2):R41. doi: 10.1186/gb-2008-9-2-r41 (PMC2374700; doi:10.1186/gb-2008-9-2-r41)
Supplement: Additional data file 6 — Presented is a table of the quantitative RT-PCR analysis of gene copy number relative to the JEC21 genome. [file gb-2008-9-2-r41-S6.doc]

| Additional data file 6. Quantitative RT-PCR analysis of gene copy number relative to the JEC21 genome | | | | | | | |
| --- | --- | --- | --- | --- | --- | --- | --- |
| Gene | Strain | Ct(Avg) test gene  (SD) | Ct (Avg) Actin  (SD) | ΔCt_gene_ (Avg. test^a^ gene – Avg. Actin) | ΔCt_ref_. (Avg. JEC21 gene– Avg. Actin) | ΔΔCt (Avg. ΔCt_gene_ –Avg. ΔCt_ref_. (JEC21) | Normalized gene copy number relative to JEC21  (2^-ΔΔCt^) |
| CNN00820 | JEC21 | 19.32 (0.02) | 19.02 (0.09) | 0.29 | 0.29 | 0.00 | 1.00 |
|  | WM626 | 18.93 (0.07) | 19.57 (0.05) | -0.63 | 0.29 | -0.94 | 1.92 |
|  | WM626* | 18.74 (0.10) | 19.46 (0.06) | -0.72 | 0.29 | -1.02 | 2.02 |
|  | CBS7779 | 18.96 (0.04) | 19.30 (0.06) | -0.34 | 0.29 | -0.64 | 1.56 |
|  | CBS7779* | 19.43 (0.03) | 19.07 (0.08) | 0.36 | 0.29 | 0.06 | 0.96 |
|  |  |  |  |  |  |  |  |
| CNN01890 | JEC21 | 17.98 (0.05) | 19.02 (0.09) | -1.05 | -1.05 | 0.00 | 1.00 |
|  | WM626 | 17.33 (0.02) | 19.57 (0.05) | -2.24 | -1.05 | -1.19 | 2.28 |
|  | WM626* | 18.22 (0.08) | 19.46 (0.06) | -1.24 | -1.05 | -0.19 | 1.14 (one copy deleted) |
|  | CBS7779 | 17.24 (0.08) | 19.30 (0.06) | -2.06 | -1.05 | -1.01 | 2.01 |
|  | CBS7779* | 35.17 (0.88) | 19.07 (0.08) | 16.10 | -1.05 | 17.15 | 0.00 (deleted gene) |
|  |  |  |  |  |  |  |  |
| SMG1^b^ | JEC21 | 17.57 (0.04) | 19.02 (0.09) | -1.46 | -1.46 | 0.00 | 1.00 (two copies in JEC21) |
|  | WM626 | 19.33 (0.17) | 19.57 (0.05) | -0.24 | -1.46 | 1.22 | 0.43 |
|  | WM626* | 19.15 (0.02) | 19.46 (0.06) | -0.31 | -1.46 | 1.15 | 0.45 |
|  | CBS7779 | 18.96 (0.05) | 19.30 (0.06) | -0.34 | -1.46 | 1.12 | 0.46 |
|  | CBS7779* | 18.75 (0.03) | 19.07 (0.08) | -0.32 | -1.46 | 1.14 | 0.45 |
|  |  |  |  |  |  |  |  |
| CNN02400 | JEC21 | 16.74 (0.11) | 19.02 (0.09) | -2.29 | -2.29 | 0.00 | 1.00 |
|  | WM626 | 16.09 (0.01) | 19.57 (0.05) | -3.48 | -2.29 | -1.19 | 2.28 |
|  | WM626* | 16.00 (0.10) | 19.46 (0.06) | -3.46 | -2.29 | -1.17 | 2.25 |
|  | CBS7779 | 16.15 (0.02) | 19.30 (0.06) | -3.15 | -2.29 | -0.86 | 1.82 |
|  | CBS7779* | 16.58 (0.10) | 19.07 (0.08) | -2.49 | -2.29 | -0.20 | 1.15 |
|  |  |  |  |  |  |  |  |

^a^Test is the strain shown on the left

^b^ *SMG1* serves as a control for detection of two-fold differences in gene copy number. WM626* and CBS7779* indicate the gene replacement transformants; note that the latter strain appears to be monosomic for Chr13.
