# Supplementary material for: Comparative hybridization reveals extensive genome variation in the AIDS-associated pathogen Cryptococcus neoformans
Source: Genome Biol. 2008 Feb 22;9(2):R41. doi: 10.1186/gb-2008-9-2-r41 (PMC2374700; doi:10.1186/gb-2008-9-2-r41)
Supplement: Additional data file 8 — Presented is a document describing the genomic hybridization analysis of transformants carrying a replacement at the APT1 gene on chromosome 13 in strains H99, CBS7779, and WM626. This file also presents an estimation of relative copy number by quantitative real-time PCR of selected markers on chromosome 13 in strains H99, JEC21, CBS7779, and WM626. [file gb-2008-9-2-r41-S8.doc]

## Additional data file 8

**Genomic hybridization analysis of transformants carrying a replacement at the *APT1* gene on Chr 13 in strains H99, CBS7779 and WM626.**

A gene replacement approach was used to confirm the CGH result that more than one copy of Chr 13 was present in strains CBS7779 and WM626 (Table 1). Specifically, the *APT1* gene on Chr 13 was targeted for replacement with a neomycin resistance marker in H99, CBS7779 and WM626 with the goal of obtaining a deletion in the single copy in H99 and in one of the (hypothesized) two copies in the other strains. *APT1* encodes a putative P-type ATPase in strain H99 based on the annotation from the Broad database (designated CNAG06469 in strain H99 and CNN01890 in strain JEC21). The constructs used for the deletion were made by a modified overlapping PCR strategy [92], and the primers used for the construct preparation and for transformant screening by colony-PCR are listed in Additional data file 14. The primer combinations were used with genomic DNA from each strain. As shown in Additional data file 7, transformation of the deletion construct into the strains with subsequent analysis of transformants by colony PCR and genomic hybridization identified strains with insertions at *APT1*. The H99 transformants had the genotype expected for replacement of the single copy of the gene to yield a disruption mutant. For WM626, transformants were identified that had one wild-type copy of the *APT1* gene and one copy that was replaced with the neomycin marker, thus supporting the hypothesis that more than one copy of Chr 13 is present in this strain. For CBS7779, we found that the hybridization patterns of the transformants with the neomycin marker were identical to those of the H99 mutant (data not shown) indicating either that *APT1* was not present in more than one copy in the original strain, that strain CBS7779 is a mixed population in which only some cells have more than one copy of Chr 13, or that both copies were replaced. As described below, estimates of relative copy number by quantitative real-time PCR of the neomycin marker and three other genes on Chr 13 in selected CBS7779 transformants revealed the presence of a single copy at all of the loci. In contrast, parallel analysis of the original CBS7779 strain supported the CGH result that more than one copy of Chr 13 is present. We hypothesize that copy number instability for Chr 13 resulted in the loss of one copy during the transformation procedure. These results support the conclusion that the population of cells for strain CBS7779 is heterogeneous in that some have one copy and some have two copies of Chr 13.

The insertion of the neomycin marker at the *APT1* locus presented an opportunity to assess whether loss of the gene resulted in specific phenotypes. We found that the H99 replacement mutant showed increased sensitivity to nitrosative stress at 37^0^C (poor growth on medium with 4 mM NaNO_2_) and that this phenotype was also seen for the replacement mutants of CBS7779. This is consistent with loss of the *APT1* gene upon transformation of these strains. The WM626 transformants displayed the wild-type phenotype, a result that is consistent with replacement of only one of the two copies of *AGT1* (data not shown). The phenotypic analysis also revealed differences between the wild-type strains of H99, WM626 and CBS7779 [Additional data file 9]. Specifically, WM626 shows relatively poor growth compared with the other strains except in the presence of miconazole or amphotericin B (amph B) where greater resistance is observed. Stain CBS7779 was similar to H99 except that it showed enhanced growth on raffinose (2%) as the carbon source. Both WM626 and CBS7779 showed reduced melanin production when compared with H99 on medium containing L-Dopa [93]. These differences could potentially result from the many regions of differences between the strains that were detected by CGH [Additional data file 4], the copy number differences for Chr 13 and/or undetected polymorphisms in specific genes.

**Estimation of relative copy number by quantitative real-time PCR of selected markers on Chr 13** **in strains H99, JEC21, CBS7779 and WM626.**

Based on the genome sequences of strains H99 and JEC21, five loci were chosen for evaluating the copy number of Chr 13 in strains CBS7779 and WM626. The loci included three from Chr 13 (CNN00820, CNN001890, CNN02400) and the actin gene (CNA04650) for use as a single copy control to normalize the PCR cycle numbers. The actin gene is located on Chr 1 and the genome sequences of H99 and JEC21 indicate the presence of a single copy. The CGH data also indicate a single copy of this gene in CBS7779 and WM626. Additionally, the gene *SMG1* was selected as a control for copy number detection because this gene is duplicated in strain JEC21 but not in strain H99 [67]. Each sample was analyzed in triplicate and cycle thresholds (Ct) were determined with standard deviations. Genomic DNA from strains H99 (Additional data file 5) and JEC21 [Additional data file 6] was used as a reference to calculate copy number. Based on the methods of Livak and Schmittgen [94] and Ferreira *et al.* [95], the average Ct for each gene and strain was determined along with the average Ct for the actin gene for each strain. The ΔCt for each gene relative to the actin gene (ΔCt_gene_) in the test strain was determined along with the ΔCt for the same gene in the reference genome relative to the actin gene (ΔCt_ref._). The ΔΔCt was calculated by subtracting the ΔCt of each test gene from the reference ΔCt in the same experiment (i.e. ΔΔCt = ΔCt_gene_ - (ΔCt_ref._). The ΔΔCt was then used to determine relative copy number with the equation 2^-ΔΔCt^.

As shown in Additional data file 5, the copy number for the *SMG1* gene*,* which is known to be duplicated in JEC21, was 1.85 when normalized to the single copy in the H99 genome. This gene was also present in single copy in WM626 and CBS7779 as indicated by the CGH data. In contrast, genes on Chr 13 (CNN00820, CNN01890, CNN02400) showed copy numbers between one and two in WM626 and CBS7779. These results suggest that the copy number of Chr 13 is variable in the population of cells for each strain with a greater copy number seen for WM626. This is consistent with the gene replacement experiment described above [Additional data file 7]. The transformants of WM626 and CBS7779 were also analyzed by quantitative real-time PCR to confirm the gene replacements detected by genomic hybridization and to confirm differences in Chr 13 copy number between strains [Additional data file 6]. As shown in Additional data file 6, the *SMG1* gene was present in one copy in WM626 and CBS7779, and representative gene replacement transformants of these strains. The analysis of genes CNN0820 and CNN02400 again confirmed that Chr 13 was present in more than one copy in WM626 and CBS7779, and the WM626 transformant. However, these genes were present in apparent single copy in the CBS7779 transformant as expected from the genomic hybridization analysis. The gene CNN01890 was the site of the gene replacement with the neomycin marker. In agreement with the hybridization data, one copy of this gene remained in the transformant of WM626 and no copies were detected in the CBS7779 transformant [Additional data file 6]. Quantitative real-time PCR analyses with the primers for the neomycin marker confirmed one copy of the marker in the WM626 and CBS7779 transformants (data not shown).
