# Supplementary material for: Comparative hybridization reveals extensive genome variation in the AIDS-associated pathogen Cryptococcus neoformans
Source: Genome Biol. 2008 Feb 22;9(2):R41. doi: 10.1186/gb-2008-9-2-r41 (PMC2374700; doi:10.1186/gb-2008-9-2-r41)
Supplement: Additional data file 10 — Presented is a table of the average Log2 ratios of the chromosomes in three AD hybrid strains upon hybridization to the tiling arrays of the JEC21 and H99 genomes. [file gb-2008-9-2-r41-S10.doc]

| **Additional data file 10. Average Log2 ratios of the chromosomes in three AD hybrid strains upon hybridization to the tiling arrays of the JEC21 and H99 genomes.^a^** | | | | | | |
| --- | --- | --- | --- | --- | --- | --- |
|  | **CDC228** | | **KW5** | | **CDC304** | |
|  | JEC21  array | H99  array | JEC21  array | H99  array | JEC21  array | H99  array |
| Chr 1 ✓^c^ | -0.837^b^ | 0.388 | -0.642 | 0.386 | -0.888 | 0.171 |
| Chr 2 ✓ | 0.296 | 0.099 | 0.271 | -0.055 | 0.462 | 0.109 |
| Chr 3 ✓ | 0.120 | -0.034 | 0.281 | -0.085 | 0.061 | 0.085 |
| Chr 4 | 0.069 | -0.075 | 0.234 | -0.098 | 0.033 | 0.088 |
| Chr 5 ✓ | 0.128 | -0.095 | 0.288 | -0.091 | 0.480 | -0.983 |
| Chr 6 ✓^d^ | 0.113 | -0.057 | -0.741 | 0.389 | 0.077 | 0.083 |
| Chr 7 | 0.115 | -0.046 | -0.752 | 0.403 | 0.079 | 0.087 |
| Chr 8 | 0.077 | -0.196 | 0.575 | -1.361 | 0.034 | -0.073 |
| Chr 9 ✓ | 0.108 | -0.038 | 0.271 | 0.087 | 0.068 | 0.075 |
| Chr 10 ✓ | 0.086 | -0.036 | -0.076 | 0.321 | 0.052 | 0.100 |
| Chr 11 | 0.117 | 0.055 | 0.253 | -0.072 | -0.016 | 0.022 |
| Chr 12 | 0.087 | -0.065 | 0.230 | -0.151 | 0.039 | 0.041 |
| Chr 13 | 0.118 | -0.097 | 0.285 | -0.126 | 0.038 | 0.022 |
| Chr 14 | 0.052 | -0.097 | 0.263 | -0.112 | 0.011 | 0.020 |

^a^ In most cases, each chromosome had both a serotype D copy and a serotype A copy as detected by Log2 ratios between –0.151 and 0.462. Variability for specific chromosomes may reflect divergence between the genomes of the parental strains that formed the AD hybrids and the reference genomes from JEC21 and H99.

^b^ The Log2 ratios suggested the loss of specific chromosome sequences from only one serotype (missing serotype is highlighted in yellow).

^c^ Red check marks represent chromosomes that have been confirmed by PCR-RFLP in this study.

^d^ Blue check marks represent chromosomes that have been analyzed by PCR analysis (Lengeler et al. 2001).
