# Supplementary material for: Comparative hybridization reveals extensive genome variation in the AIDS-associated pathogen Cryptococcus neoformans
Source: Genome Biol. 2008 Feb 22;9(2):R41. doi: 10.1186/gb-2008-9-2-r41 (PMC2374700; doi:10.1186/gb-2008-9-2-r41)
Supplement: Additional data file 12 — Presented is a tabulated list of Cryptococcus neoformans and C. gattii strains. [file gb-2008-9-2-r41-S12.doc]

| **Additional data file 12. List of *Cryptococcus neoformans* and *C. gattii* strains** | | | | | |
| --- | --- | --- | --- | --- | --- |
|  |  |  |  |  |  |
| Strain | Serotype | Mating type | Molecular subtype | Source | Reference |
| JEC21 | D | α | VNIV (AFLP 2) | J. Heitman | Heitman et al. 1999 |
| NIH12 | D | α | VNIV (AFLP 2) | J. Kwon-Chung | Heitman et al. 1999 |
| NIH433 | D | a | VNIV (AFLP 2) | J. Kwon-Chung | Heitman et al. 1999 |
| H99 | A | α | VNI (AFLP 1) | J. Heitman | Heitman et al. 1999 |
| CBS7779 | A | α | VNI (AFLP 1) | T. Boekhout | Boekhout et al. 1997 |
| Bt63 | A | a | VNB | J. Heitman | Lintvintseva et al., 2006 |
| 125.91 | A | a | VNI (AFLP 1) | J. Heitman | Lengeler et al., 2000 |
| WM626 | A | α | VNII (AFLP 1A) | W. Meyer | Meyer et al. 1999 |
| CDC228 | AD | a (A), α (D) | VNIII (AFLP 3) | J. Heitman | Lengeler et al. 2001 |
| KW5 | AD | α (D), a (A) | VNIII (AFLP 3) | J. Heitman | Lengeler et al. 2001 |
| CDC304 | AD | a (A), α (D) | VNIII (AFLP 3) | J. Heitman | Lengeler et al. 2001 |
| E566 | B | a | VG? | J. Heitman | Fraser et al. 2004 |
| WM276 | B | α | VGI (AFLP 4) | J. Heitman | Fraser et al. 2004 |
