# Supplementary material for: Comparative hybridization reveals extensive genome variation in the AIDS-associated pathogen Cryptococcus neoformans
Source: Genome Biol. 2008 Feb 22;9(2):R41. doi: 10.1186/gb-2008-9-2-r41 (PMC2374700; doi:10.1186/gb-2008-9-2-r41)
Supplement: Additional data file 13 — Presented is a sample key for the CGH data. [file gb-2008-9-2-r41-S13.doc]

# Additional data file 13. Sample key for the CGH data

# Serotype D

ORD_ID CHIP_ID IMAGE_NAME DYE DESIGN_NAME DESIGN_ID/SAMPLE_LABEL SAMPLE_SPECIES SAMPLE_DESCRIPTION

1497 45287 45287_532.tif Cy3 2005-04-28_JEC21_WG_CGH 1954 SOM009YP Cryptococcus neoformans NIH433 Serotype D

1497 45287 45287_635.tif Cy5 2005-04-28_JEC21_WG_CGH 1954 SOM009YL Cryptococcus neoformans JEC21 Serotype D

1859 61662 61662_532.tif Cy3 2005-04-28_JEC21_WG_CGH 1954 SOM00H5H Cryptococcus neoformans NIH12 Serotype D

1859 61662 61662_635.tif Cy5 2005-04-28_JEC21_WG_CGH 1954 SOM00H5G Cryptococcus neoformans JEC21 Serotype D

**Serotype A**

ORD_ID CHIP_ID IMAGE_NAME DYE DESIGN_NAME DESIGN_ID/SAMPLE_LABEL SAMPLE_SPECIES SAMPLE_DESCRIPTION

1860 56071 56071_532.tif Cy3 2005-10-06_H99_WG_CGH 2661 SOM00H51 Cryptococcus neoformans 125.91 Serotype A

1860 56071 56071_635.tif Cy5 2005-10-06_H99_WG_CGH 2661 SOM00H50 Cryptococcus neoformans H99 Serotype A

1860 61626 61626_532.tif Cy3 2005-10-06_H99_WG_CGH 2661 SOM00H5C Cryptococcus neoformans WM626 Serotype A

1860 61626 61626_635.tif Cy5 2005-10-06_H99_WG_CGH 2661 SOM00H5E Cryptococcus neoformans H99 Serotype A

1860 61655 61655_532.tif Cy3 2005-10-06_H99_WG_CGH 2661 SOM00H5F Cryptococcus neoformans CBS7779 Serotype A

1860 61655 61655_635.tif Cy5 2005-10-06_H99_WG_CGH 2661 SOM00H5D Cryptococcus neoformans H99 Serotype A

1860 55716 55716_532.tif Cy3 2005-10-06_H99_WG_CGH 2661 SOM00H4Z Cryptococcus neoformans BT63 Serotype A

1860 55716 55716_635.tif Cy5 2005-10-06_H99_WG_CGH 2661 SOM00H4Y Cryptococcus neoformans H99 Serotype A

**Serotype AD**

ORD_ID CHIP_ID IMAGE_NAME DYE DESIGN_NAME DESIGN_ID/SAMPLE_LABEL SAMPLE_SPECIES SAMPLE_DESCRIPTION

1860 59091 59091_532.tif Cy3 2005-10-06_H99_WG_CGH 2661 SOM00H55 Cryptococcus neoformans KW5 Serotype AD

1860 59091 59091_635.tif Cy5 2005-10-06_H99_WG_CGH 2661 SOM00H54 Cryptococcus neoformans H99 Serotype A

1497 44150 44150_532.tif Cy3 2005-04-28_JEC21_WG_CGH 1954 SOM009YM Cryptococcus neoformans KW5 Serotype AD

1497 44150 44150_635.tif Cy5 2005-04-28_JEC21_WG_CGH 1954 SOM009YL Cryptococcus neoformans JEC21 Serotype D

1860 69001 69001_532.tif Cy3 2005-10-06_H99_WG_CGH 2661 SOM00H57 Cryptococcus neoformans CDC228 Serotype AD

1860 69001 69001_635.tif Cy5 2005-10-06_H99_WG_CGH 2661 SOM00H56 Cryptococcus neoformans H99 Serotype A

1497 44099 44099_532.tif Cy3 2005-04-28_JEC21_WG_CGH 1954 SOM009YK Cryptococcus neoformans CDC228 Serotype AD

1497 44099 44099_635.tif Cy5 2005-04-28_JEC21_WG_CGH 1954 SOM009YL Cryptococcus neoformans JEC21 Serotype D

1860 61194 61194_532.tif Cy3 2005-10-06_H99_WG_CGH 2661 SOM00H59 Cryptococcus neoformans CDC304 Serotype AD

1860 61194 61194_635.tif Cy5 2005-10-06_H99_WG_CGH 2661 SOM00H58 Cryptococcus neoformans H99 Serotype A

1497 44479 44479_532.tif Cy3 2005-04-28_JEC21_WG_CGH 1954 SOM009YN Cryptococcus neoformans CDC304 Serotype AD

1497 44479 44479_635.tif Cy5 2005-04-28_JEC21_WG_CGH 1954 SOM009YL Cryptococcus neoformans JEC21 Serotype D
