# Supplementary material for: Comparative hybridization reveals extensive genome variation in the AIDS-associated pathogen Cryptococcus neoformans
Source: Genome Biol. 2008 Feb 22;9(2):R41. doi: 10.1186/gb-2008-9-2-r41 (PMC2374700; doi:10.1186/gb-2008-9-2-r41)
Supplement: Additional data file 14 — Presented is a tabulated list of primer sequences. [file gb-2008-9-2-r41-S14.doc]

| **Additional data file 14. List of primer sequences** | | |
| --- | --- | --- |
| Primer Name | Sequence Forward (5’-3’) | Sequence Reverse (5’-3’) |
| acidphos F / R | CCAGCTTTTACGGCCTCAAC | GCCACTTGTCTCCACCATGA |
| ino3phos F / R | CGCTGACATGGGCTACTACA | AGATGGGCACAAAGACATCC |
| thiolox F / R | CTACCCGCTCCAGGAGAATG | CGCGACTAAGGATGGTAGGG |
| CNA01230 F / R | GGTGTCAAGACTGTCACCATCTAC | AACGACCTGAGTATTGGCCTTG |
| CNA01240 F / R | CGCAGAACCAGACAGTGTAGAAGTAT | AGTGGCTATACAAATCCAGAAGACAAT |
| CNA04650 F / R | CGTCACAAACTGGGACGACAT | GCGACACGGAGCTCATTGTA |
| CNA04800 F / R | GTAGGGCAGGGGACAGATAGGAC | CTCCAGACTGATATCCGTGTACGTC |
| CNA06230 F / R | CAAGAAATACCTCTAACTGCAGACAC | CAGAATATAATCATCTTGCTGATCTGA |
| CNB01970 F / R | GTTCATGAGAGACAACGTGCAG | CGATATCCTCACTAATTGACGAAC |
| CNC03930 F / R | TAGTGTATATTCTATGTGTTCCGCCTGAT | ACATTGTGAGATATAACCCAGAGGCAGTTA |
| CNC06920 F / R | TTTCTTGACATCGATCCCTTCT | AAAGCTACGGGACCTTTACTCC |
| CNC06920 F2 / R2 | GATCCCTTCTCCGTTCGTTTGA | CCTTTACTCCCATGTCGCTGCT |
| CNE04380 F / R | GAACGACGCTCCTTTGATTACC | CTCCTCTTCGATCTCATCTCTTCC |
| CNG00780 F / R | TCGCTACCATCGAGAGTGTCATAG | GTCTTGCCTTACCCTCCTATCAAG |
| CNI01750 F / R | TAGATATGAGACACCCACCACACC | AACCTCGGTACTAGTGACCACCAT |
| CNJ02400 F / R | AAGAGGTCAGAGAAAGTCGGTGAG | GTCTGCAGAGTGTGTCAGGAGTTT |
| 0040H99 F / R | GTCGACAGTCTCACTTCCTCTACAC | GAGAGACTATACACGACTGACCG |
| CNN00820 F / R | AGAGGCCAAAAGACCCCATA | AGCTTTGACAAGAGCAGCAAAG |
| CNN01890 F / R | GCGTCACGAGAGGGAAAATACT | TGGTAGTGACATAAGTGTAGTTGAGGAA |
| CNN02400 F / R | GGCCCCTACGCCAGCTT | CTGACCTTTAGCGAACCAAGATC |
| 03356H99 F / R | ACCCTCGCACTTGAGCAC | CTCGCTATCAGCTGCTCACT |
| 04239H99 F / R | GCCGTCATACGCCTGTATC | GGGAAGACGATCACCGAAT |
| 05782H99 F / R | ACGGACGGTGTTTTTGAGA | CGAGCCTAGGGGTAGGTAG |
| 05191H99 F / R | CTACCAGCATCTCGAGTAATAGTGAAGACA | TGTAAGAGTCTAATCGTTCTTCATGTCAGC |
| SMG1 | CCCCGTTAAGGGCCTGAT | TGGGCCAGAGTCTCGATGAG |
| APT1-1/3 | GATTCTTCAGAAATGTTTCCACCTACCAAT | AGCTCACATCCTCGCAGCGGTGATAATGTTTTTG  CGTTAGCGCGGT |
| APT1-2/5 | ACCGCGCTAACGCAAAAACATTATCACCGC  TGCGAGGATGTGAGCT | ACAAAACAGCCTACAAGCCTCCTATAGCAGAAGA  GATGTAGAAACTA |
| APT1-4/6 | TAGTTTCTACATCTCTTCTGCTATAGGAGGC  TTGTAGGCTGTTTTGT | TGTACTGACTCAATTTTGCAGAGAGCT |
| APT1-7NE/8NE | CCATCCATCCTATCACATTGGAAACGAC | CGTGACACCATCCTTCTTGTACTTGATATC |
| APT1-9PO/NEO-PO | CCGAGGTAGCCAAATTTCGACGTTTTGTGC | GCCACTCGAATCCTGCATGCTTATGTGAGT |
