# Supplementary material for: Comparative hybridization reveals extensive genome variation in the AIDS-associated pathogen Cryptococcus neoformans
Source: Genome Biol. 2008 Feb 22;9(2):R41. doi: 10.1186/gb-2008-9-2-r41 (PMC2374700; doi:10.1186/gb-2008-9-2-r41)
Supplement: Additional data file 15 — Presented are the figure legends for the figures presented in Additional data files 3, 7, 9 and 11. [file gb-2008-9-2-r41-S15.doc]

**Figure Legends**

**Figure legend – Additional data file 3. Variation in the genomes of strains representing the three molecular subtypes of the A serotype of *C. neoformans*.**

The Log2 ratios for the regions of similarity and difference are shown for all of the chromosomes in the VNI strains 125.91 and CBS7779, the VNII strain WM626 and the VNB strain Bt63. The tiling array for the analysis was based of the genome of strain H99.

**Figure legend – Additional data file 7. Integration of a marker at the *APT1* gene to confirm disomy of Chr 13.**

(A). Genotype analysis by PCR (**a**) and confirmation by Southern blot analysis (**b**) of the wild-type strain, H99, and the *apt1* mutant strains. In **a**, a band of ~1 kb in the screen with the primer pair APT1-9PO and NEO-PO (left photograph) indicates replacement of the target gene with the neomycin marker, and a band of 460 bp in the screen using primers APT1-7NE and APT1-8NE (right photograph) indicates the presence of the wild-type genotype. The analysis revealed a single allele of *APT1* in H99 wild-type and the *apt1* mutant strains. For the Southern blot analysis (**b**), genomic DNA (10 μg) of the wild-type and four representative mutant strains was digested with *ClaI* and hybridized with a 1 kb fragment upstream of the *APT1* gene. The wild-type strain shows two bands: one of 7.5 kb and another of 3.4 kb. Replacement of the wild-type locus with the neomycin marker construct resulted in a shift of the larger band to 4.5 kb and retention of the 3.4 kb band. (B). Genotype analysis by PCR (**a**) and confirmation by Southern blot analysis (**b**) of the WM626 strain and the corresponding *apt1* mutants. A band of ~1 kb in the screen using primer pair APT1-9PO and NEO-PO (left photograph) indicated a replacement of the target gene, and a band of 460 bp in the screen with primers APT1-7NE and APT1-8NE (right photograph) indicates the presence of wild-type genotype. The PCR analysis demonstrated the presence of a mutated *APT1* allele and a wild-type allele in the mutant strains. For Southern blot analysis (**b**), genomic DNA (10 μg) of the wild-type and four representative mutant strains was digested with *ClaI* and hybridized with a 1 kb fragment upstream to *APT1* gene (left arm for the deletion construct in WM626). The wild-type strain showed bands of 7.5 kb and 3.4 kb, as seen with H99. The mutants (lanes 1, 3, and 4), on the other hand, showed three bands, two bands in the same sizes as those in the wild-type strain, and an additional band of 4.5 kb. The latter band is the size expected for replacement of one of the two alleles of *APT1* in WM626. Lane 2 indicates an ectopic insertion of the *APT1* deletion construct in the strain. The primer sequences used for this analysis are listed in Additional data file 14.

**Figure legend – Additional data file 9. Phenotypic variation for strains H99, CBS7779 and WM626.**

Cultures of the three strains were spotted at 10^5^ cells on the left with 10-fold dilutions from left to right on each plate. The plates were incubated at the two temperatures indicated for three days. With the exception of the top panel, yeast nitrogen base (YNB) medium was used for each of the assays. The medium with raffinose contained 2%.

**Figure legend – Additional data file 11. RFLP-PCR analysis of the origin of Chr 1 in 16 AD hybrid strains.**

The CNA01230 gene on Chr 1 was amplified with primers CNA01230 F / R and the PCR product was digested with Ava1. DNA from JEC21 was used to represent the serotype D genome and DNA from H99 represented the serotype A genome. Lanes 4 to 11 in each panel show the results for the 16 AD isolates (MAS92- strains) that were obtained from clinical specimens by Yan *et al*., [96].
